# Genomic analysis identifies candidate SOS-associated and EPS/envelope-remodeling islands in a drinking-water *Stenotrophomonas maltophilia* complex isolate

**DOI:** 10.1038/s41598-026-58581-0

**Published:** 2026-06-28

**Authors:** Farouk Hassan, Süheyla Türkyılmaz, Taghrid S. El-Mahdy

**Affiliations:** 1 Department of Microbiology and Immunology, Faculty of Pharmacy, Modern University for Technology and Information, Cairo, Egypt; 2 Department of Microbiology, Faculty of Veterinary Medicine, Aydın Adnan Menderes University, Aydın, Türkiye; 3 Department of Microbiology and Immunology, Faculty of Pharmacy, Capital (formerly Helwan) University, Cairo, Egypt

**Keywords:** *Stenotrophomonas maltophilia* complex, Drinking-water distribution system (DWDS), Genomic islands, Persistence, Biofilm adaptation, Environmental surveillance, Computational biology and bioinformatics, Microbiology

## Abstract

Drinking-water distribution systems (DWDS) impose ecological pressures shaped by oligotrophy, surface attachment, hydraulic fluctuation, and residual disinfectants. These conditions may favor stress-tolerant and biofilm-capable microorganisms, including members of the *Stenotrophomonas maltophilia* complex. Although this complex is clinically relevant because of intrinsic multidrug resistance and opportunistic pathogenic potential, the strain-level genomic features that may support persistence in disinfectant-managed water systems remain incompletely characterized. We applied an integrated genome-resolved framework to *Stenotrophomonas* sp. NG-SM01, a drinking-water isolate assigned to the *S. maltophilia* complex genomospecies Sgn4. Hybrid sequencing was used to generate a closed genome assembly, followed by phylogenomics and MLST for taxonomic placement, pangenome analysis for gene-content context, and genomic-island mapping to identify candidate accessory regions. Functional profiling included BacMet screening for metal/biocide tolerance-associated loci and genome-scale metabolic reconstruction using gapseq. Phenotypic assays assessed crystal-violet biofilm biomass and acute hydrogen peroxide (H₂O₂) survival as a general oxidative-stress proxy. NG-SM01 clustered within the Sgn4 genomospecies of the *S. maltophilia* complex and formed a strongly supported sister lineage to its closest available reference genome, GCF_025642255.1 (UFboot = 100). MLST confirmed a newly curated *guaA* allele (*guaA*-909), and the allelic profile was assigned ST1409. Disk diffusion showed limited inhibition by several antimicrobial agents; however, categorical interpretation using *S. maltophilia*-specific CLSI criteria was applied only to levofloxacin and trimethoprim–sulfamethoxazole, both of which were susceptible. Genomic-island analysis highlighted two candidate loci potentially relevant to stress adaptation: GI_6, carrying an EPS/envelope-remodeling cassette including *algL* and a GT26-family glycosyltransferase within a panel-restricted architecture, and GI_4, an IME-associated region located near SOS-response genes including *recA*, *recX*, and *lexA*. NG-SM01 formed reproducible moderate biofilm biomass under static microtiter conditions and showed plateau-like survival dynamics during acute H₂O₂ exposure. BacMet screening prioritized Tier-1 metal/biocide tolerance-associated signals, including CzcR-like and AdeL-like regulators and a YfeB-like transport component, while gapseq reconstruction predicted broad transport capacity, including 1,920 transporter entries, of which 23% were metal-related. This single-isolate study identifies genomic and phenotypic features potentially relevant to persistence in a drinking-water *S. maltophilia* complex Sgn4 isolate. The combined evidence supports a hypothesis-generating “Switch–Shield” framework, in which stress-response regulation and mobilome-associated plasticity may represent a candidate “switch,” while EPS/envelope remodeling may represent a candidate protective “shield.” However, direct causality between GI_4/GI_6 and the observed phenotypes was not demonstrated. Future validation using transcriptomics, mutant-based assays, and DWDS-mimetic free-chlorine or chloramine exposure models, including flow/pipe reactors with detachment and shedding measurements, will be required to test this model.

**Supplementary Information:**

The online version contains supplementary material available at 10.1038/s41598-026-58581-0.

## Introduction

Drinking-water distribution systems (DWDS) are engineered to suppress microbial growth, but they also create ecological conditions that can favor microorganisms able to tolerate low nutrient availability, surface-associated growth, hydraulic fluctuation, and residual disinfectant exposure. After water leaves the treatment plant, microbial cells encounter oligotrophy, flow–stagnation cycles, extensive pipe surface area, and repeated opportunities for attachment. Biofilms are therefore an important ecological state in DWDS, where they can increase disinfectant demand, contribute to residual decay, and intermittently release cells into bulk water, including at distal points of the network^1–3^. Residual disinfectants, particularly free chlorine, remain central to secondary disinfection, and operational guidance commonly emphasizes maintaining a measurable residual throughout the distribution network; minimum residual targets around 0.2 mg/L at distal points and operational ranges such as 0.2–0.5 mg/L are frequently cited, although these values are context- and jurisdiction-dependent^4–6^. However, chlorination does not sterilize DWDS. Instead, residual disinfectant exposure can reshape microbial community composition and may favor organisms with stress-tolerance and surface-associated survival traits. Within biofilms, cells embedded in extracellular polymeric substance (EPS) matrices may experience reduced effective exposure to biocides because of matrix-associated protection and local microenvironmental heterogeneity^2,3,7^. This persistence challenge is increasingly relevant from a One Health perspective because disinfectant-associated oxidative stress may intersect with antimicrobial resistance (AMR) ecology. Previous studies and reviews have suggested that chlorination can contribute to selective and mutational pressures and may influence horizontal gene transfer through processes such as reactive oxygen species generation, altered membrane permeability, SOS-response induction, and efflux-system activation^8^. Accordingly, traits related to stress buffering, surface or EPS remodeling, regulatory plasticity, and nutrient acquisition may be important components of DWDS persistence, although their contribution requires strain-level and functional validation^1–3,8^. Among organisms positioned at this environmental–clinical interface is the *Stenotrophomonas maltophilia* complex. Members of this complex are environmentally widespread, biofilm-capable, and clinically relevant opportunistic pathogens, particularly in vulnerable hosts such as immunocompromised patients, where they can cause respiratory, bloodstream, and other healthcare-associated infections^9–12^. Their intrinsic multidrug resistance and limited therapeutic options increase clinical concern, while environmental traits such as biofilm formation, oxidative-stress tolerance, and metabolic flexibility may be relevant to persistence in low-nutrient engineered water environments^9–12,14^. However, most DWDS studies emphasize engineering aspects of residual decay, community structure, or biofilm control, whereas fewer studies combine strain-resolved genomics with targeted phenotyping to examine how specific environmental lineages may persist in disinfectant-managed systems. Resistome-centered analyses alone may also underrepresent broader ecological fitness traits, including genomic-island architecture, mobilome-associated plasticity, envelope or EPS remodeling, regulatory capacity, and nutrient-acquisition potential. These features may help generate hypotheses about persistence in oligotrophic and oxidative environments, but they do not by themselves establish causality. To address this gap, we characterized NG-SM01, a *Stenotrophomonas maltophilia* complex isolate recovered during routine drinking-water quality monitoring. We combined targeted phenotyping relevant to surface-associated growth and oxidative-stress survival with genome-resolved analyses of taxonomic placement, pangenome context, genomic islands, mobilome structure, metal/biocide tolerance-associated loci, and genome-scale metabolic potential using gapseq^13^. Together, these analyses provide a single-isolate, hypothesis-generating framework for identifying candidate genomic and phenotypic features potentially relevant to DWDS-associated persistence and for prioritizing targets for future validation under drinking-water-mimetic disinfectant conditions.

## Materials and methods

### Study design and sample source

NG-SM01 was recovered from routine drinking-water quality-control samples submitted to the microbiology laboratory as part of standard environmental surveillance. Sampling was performed exclusively for monitoring purposes and did not involve human or animal procedures; therefore, ethical approval was not required. NG-SM01 is the internal laboratory identifier assigned to this *Stenotrophomonas* isolate. Because the isolate was recovered through routine quality-control monitoring rather than a dedicated epidemiological sampling campaign, only limited non-identifying contextual metadata were available. Detailed information on residual disinfectant concentration, exact sampling-point category, hydraulic status, and collection-period metadata was not available for analysis. The isolate was archived as glycerol stocks at − 80 °C and revived from frozen stocks for all experiments.

### Isolation and purification by chromogenic membrane filtration

Water samples were processed by chromogenic membrane filtration using CHROMagar™ ECC membranes and CHROMagar™ ECC agar, followed by aerobic incubation at 37 °C for 18–24 h. CHROMagar™ ECC is designed primarily for the detection and differentiation of coliforms and *Escherichia coli* in water samples. During routine plate examination, a pale, atypical colony that did not show the expected chromogenic appearance of target coliform/*E. coli* colonies was selected for further characterization. The colony was repeatedly subcultured to purity and subsequently identified as a *Stenotrophomonas* isolate. The purified isolate was stored at − 80 °C in Trypticase Soy Broth (Oxoid, UK) containing 20% (v/v) glycerol (Sigma-Aldrich, USA).

### Phenotypic characterization

Gram staining and routine biochemical tests were performed, including catalase, oxidase, coagulase, urease, indole, bile esculin hydrolysis, nitrate reduction, motility, and oxidation–fermentation testing. Growth and colony morphology were assessed on Columbia blood agar, Mueller–Hinton agar, MacConkey agar, EMB agar, and TSI agar (all Oxoid, UK) after aerobic incubation at 37 °C.

### Antimicrobial susceptibility testing

Antimicrobial susceptibility testing was performed by disk diffusion on Mueller–Hinton agar (Oxoid, UK) following CLSI methodology and interpretive guidance (CLSI M02/M100)^15^. Inocula were standardized to 0.5 McFarland, plates were incubated aerobically at 37 °C for 18–24 h, and inhibition zones were measured in millimeters. Categorical susceptible/intermediate/resistant interpretation was applied only where CLSI provides *Stenotrophomonas maltophilia*-specific disk-diffusion breakpoints, including levofloxacin and trimethoprim–sulfamethoxazole^15^. Breakpoints from other taxa were not extrapolated to *Stenotrophomonas*, because such extrapolation may generate unsupported categorical interpretations. For antimicrobial agents without *S. maltophilia*-specific CLSI breakpoints, inhibition-zone diameters were reported descriptively only. Complete absence of inhibition was reported as a 0-mm zone, indicating no measurable inhibition under the test conditions, but was not assigned a categorical resistance interpretation unless species-specific CLSI criteria were available.

### High-molecular-weight DNA extraction

High-molecular-weight genomic DNA was extracted from overnight culture using the MagAttract HMW DNA Kit (Qiagen, Germany) with lysozyme-assisted lysis at 20 mg/mL and 37 °C for 90 min. DNA concentration was measured using Qubit™ dsDNA HS assays (Thermo Fisher Scientific, USA), purity was assessed by spectrophotometry, and integrity was evaluated by 0.6% agarose gel electrophoresis.

### Hybrid sequencing, assembly, and polishing

Raw ONT and Illumina FASTQ reads underwent standard quality control, trimming, and read-summary procedures, with software and parameters listed in Supplementary Table S1. Long-read de novo assembly was performed using Flye^16^, followed by consensus improvement and short-read polishing using an established workflow including Polypolish^17^ (Supplementary Table S1).

### Assembly quality assessment and distance-based comparison

Assembly statistics were computed using QUAST^18^. Genome completeness and contamination were estimated using CheckM2^19^. Rapid distance-based comparison and preliminary placement were performed using Mash^20^ (Supplementary Table S1).

### Genome annotation and functional refinement

The final assembly was annotated using Bakta^21^ and re-annotated with Prokka^22^ to provide a standardized feature set for comparative analyses. Functional annotation was complemented by eggNOG-mapper v2^23^ (Supplementary Table S2). Protein-domain evidence was generated using InterProScan^24^ to support refinement of hypothetical proteins and loci within predicted genomic islands.

### Multilocus sequence typing (MLST) using PubMLST

MLST allele calling was performed using the PubMLST/BIGSdb framework^25^. The *Stenotrophomonas maltophilia* seven-locus scheme, comprising *atpD*, *gapA*, *guaA*, *mutM*, *nuoD*, *ppsA*, and *recA*, was applied by matching NG-SM01 loci to curated allele definitions. The NG-SM01 *guaA* locus initially lacked an exact match to existing PubMLST allele definitions and was therefore aligned across the full-length sequence to identify nucleotide differences relative to the closest curated allele. Following curator review and PubMLST acceptance, this allele was assigned as *guaA*-909, and the complete allelic profile of NG-SM01 was assigned sequence type ST1409.

### Comparative genome panels, pangenomics, and phylogenomics

Two comparative datasets were used. A 20-genome pangenome panel, comprising NG-SM01 and 19 reference genomes, was used for gene presence/absence analysis and panel-restricted gene calls. A broader phylogenomic context set was used for genomospecies placement. Pangenome reconstruction was performed using Panaroo v1.5.2 in strict mode^26^. Gene–trait association testing was performed using Scoary with Benjamini–Hochberg false-discovery-rate correction^27^. For phylogenomics, the Panaroo core-gene alignment was used to infer a maximum-likelihood tree with IQ-TREE, with ModelFinder enabled and node support assessed using 1,000 ultrafast bootstrap replicates and 1,000 SH-aLRT replicates. Trees were visualized and annotated in iTOL.

### CAZyme annotation

Carbohydrate-active enzymes (CAZymes) were annotated using dbCAN3 and retained when supported by at least two independent detection strategies (Supplementary Table S1)^28^. CAZyme classes and abbreviations are provided in Supplementary Table S3.

### Mobilome, genomic islands, and prophage profiling

Genomic islands were predicted using IslandViewer 4, including IslandPick, IslandPath-DIMOB, and SIGI-HMM^29^. Integrative and conjugative element screening was performed using ICEberg 3.0 resources^30^. Prophage regions were detected using PHASTER^31^, and predicted prophage sequences were annotated using Pharokka within Galaxy^32^. Genomic-island boundary support was evaluated by read mapping and inspection of coverage continuity and junction-spanning reads (Supplementary Table S1).

### Resistance/virulence screening, BacMet profiling, and metabolic reconstruction

Acquired AMR determinants were identified using AMRFinderPlus^33^. Additional resistome and virulence screening was performed using ABRicate^34^ against VFDB^35^ and CARD^36^ (Supplementary Table S1). Genome-scale metabolic reconstruction was performed using gapseq, extracting transporter predictions, pathway presence calls (TRUE/FALSE/UNK), minimal-medium inference under aerobic conditions, and reaction evidence tiers (rxnWeights)^13^. Metal/biocide tolerance screening using BacMet and database/tool versions are listed in Supplementary Table S1.

### Crystal violet microtiter biofilm assay

Biofilm formation was quantified using a static 96-well crystal violet (CV) microtiter assay adapted from established microtiter-dish protocols^37^. Overnight cultures of *E. coli* ATCC 25,922, *K. pneumoniae* ATCC 13,883, *P. aeruginosa* ATCC 27,853, and NG-SM01 were inoculated into flat-bottom polystyrene plates and incubated under static conditions, with uninoculated medium wells included as blanks. Biofilm measurements were obtained from five independent biological replicates, prepared from independent overnight cultures on separate days for each strain. Within each biological replicate, wells were run in triplicate and averaged to yield a single value per day, thereby reducing technical well-to-well variability without inflating the biological sample size. Planktonic growth was recorded at 600 nm (OD_600_). Wells were gently emptied, washed with PBS, fixed with methanol, stained with CV, rinsed thoroughly, air-dried, and solubilized with acetic acid before measurement at 595 nm (OD_595_). Biofilm biomass was expressed as blank-corrected OD_595_: OD_595_corr = OD_595_ sample − OD_595_ blank. A growth-normalized biofilm index was calculated as: OD_595_corr/OD_600_corr, where OD_600_corr = OD_600_ sample − OD_600_ blank.

### Hydrogen peroxide (H₂O₂) oxidative-stress survival assay

Oxidative-stress tolerance was assessed by acute hydrogen peroxide (H₂O₂) challenge followed by recovery plating, adapted from established hydrogen-peroxide sensitivity assays^38^. This assay was used as a general oxidative-stress proxy and was not intended to reproduce the chemistry of free chlorine or chloramine exposure in DWDS. NG-SM01 was tested alongside *K. pneumoniae* ATCC 13,883 and *P. aeruginosa* ATCC 27,853, while *E. coli* ATCC 25,922 served as a more H₂O₂-sensitive laboratory comparator. Three independent biological replicates were performed from fresh colonies on separate days for each strain, and within each replicate, triplicate technical spot counts were averaged to yield one value per day (*n* = 3). Overnight LB cultures incubated at 37 °C with shaking were subcultured 1:100 to mid-log phase and standardized to OD_600_ = 0.5 in PBS. A 1:10 pre-dilution was prepared using 100 µL culture and 900 µL PBS, and freshly prepared H₂O₂ (Merck, Germany; from 30% stock) was added to final concentrations of 0, 0.25, 0.5, 0.75, 1.0, 1.5, 2.0, 2.5, and 3.0 mM. Suspensions were incubated for 5 min at room temperature, then reactions were quenched with catalase (Sigma-Aldrich, Germany; final concentration 1,000 U/mL)^38^. Samples were serially 10-fold diluted in PBS to 10⁻³, and 10 µL aliquots were spot-plated on LB agar using a Miles–Misra-type drop-plate viable-count approach^39^. Plates were incubated for 18–24 h at 37 °C. Colonies were counted from spots yielding 10–30 CFU, while confluent growth was excluded. CFU/mL was calculated as CFU/mL = N × (1/V) × (1/D), where N is the number of colonies counted in the 10-µL spot, V = 0.01 mL, and D is the total dilution factor, including the initial 1:10 pre-dilution and subsequent serial dilutions. Killing was summarized as log10 reduction relative to the matched 0 mM control for each strain.

### Oxidative-stress time-kill kinetics using H₂O₂ as a proxy assay

Time-kill kinetics were assessed using a sampling-over-time design aligned with standardized bactericidal/time-kill methodology^40^. This assay was used to compare survival dynamics under acute H₂O₂-mediated oxidative stress and was not interpreted as direct evidence of free-chlorine or chloramine tolerance. For the kinetic assay, a final H₂O₂ concentration of 2.0 mM was selected based on the dose–response assay because it produced a measurable oxidative-stress challenge while still allowing recovery of countable colonies across strains, enabling comparison of survival trajectories over time. Mid-log cultures of *P. aeruginosa* ATCC 27,853, *K. pneumoniae* ATCC 13,883, NG-SM01, and *E. coli* ATCC 25,922 were standardized to OD_600_ = 0.5 in PBS and exposed to H₂O₂ under identical conditions. Aliquots were collected at 0, 5, 15, 30, and 60 min; the 0-min baseline was sampled from the paired suspension before H₂O₂ addition. At each time point, samples were diluted 1:10 in PBS, immediately neutralized with catalase at a final concentration of 1,000 U/mL^38^, then diluted 1:100 and serially diluted to generate 10⁰–10⁻³ dilutions. For each dilution, 10 µL was spot-plated in triplicate technical spots using a Miles–Misra-type drop-plate approach^39^ and incubated until colonies were countable. Colonies were counted from spots yielding 10–30 CFU, and confluent growth was excluded. Triplicate counts were averaged per dilution and time point. CFU/mL was calculated as CFU/mL = N × (1/V) × (1/D), where N is the mean colony count across triplicate 10-µL spots, V = 0.01 mL, and D is the total dilution factor, calculated as the product of all dilution steps before plating. Three independent biological replicates were analyzed on separate days from fresh colonies, with technical replicates averaged within each day (*n* = 3). Survival was reported as log_10_(CFU/mL), and kinetics were summarized as within-replicate log_10_ reduction relative to baseline: Δlog_10_ = log_10_(CFU/mL)0 min − log_10_(CFU/mL)t^40^.

### Statistical analysis and visualization

Statistical analyses were performed on Ubuntu Linux 22.04.5 LTS. Biofilm comparisons between NG-SM01 and each reference strain used two-sided Welch’s t-test^41^, with Holm correction for multiple testing^42^. Effect sizes were reported as Hedges’ g^43^ from replicate-level blank-corrected, growth-normalized biofilm indices. Acute H₂O₂ survival, expressed as log_10_ reduction, was analyzed by two-way ANOVA using strain and dose as factors, with linear dose-trend testing across 1.0–3.0 mM using regression/contrast on dose. Time-kill kinetics, expressed as Δlog_10_ reductions, were analyzed by two-way ANOVA using strain and time as factors, followed by Tukey HSD post-hoc testing. Block-level enrichment of non-hypothetical genes among NG-SM01 panel-restricted gene blocks was tested using one-sided Fisher’s exact test with Benjamini–Hochberg false-discovery-rate correction. Pangenome gene–trait associations were tested using Scoary with Benjamini–Hochberg false-discovery-rate correction^27^. Figures were generated using Python plotting libraries (Supplementary Table S1). Synteny was visualized with clinker/clustermap.js^44^, Jaccard similarity was computed from reciprocal-best-hit-based gene-content overlap across extracted windows, and circular genome maps were generated using CGView within Proksee^45^.

## Results

### Phenotypic characterization and antimicrobial susceptibility of NG-SM01

Microscopy showed Gram-negative rods (Supplementary Fig. S1). Biochemical testing supported an oxidative, non-fermentative phenotype: NG-SM01 was catalase- and oxidase-positive, motile, and nitrate-reduction positive, and negative for coagulase, urease, indole, and bile esculin hydrolysis (Supplementary Table S4). The isolate grew on MacConkey and EMB as a lactose non-fermenter, failed to grow on mannitol salt agar, produced a yellow/golden pigment on Mueller–Hinton and trypticase soy agar, showed glucose oxidation without fermentation (TSI K/K), and exhibited γ-hemolysis on blood agar (Supplementary Table S4). Disk diffusion showed limited or absent inhibition by several tested agents, including 0-mm zones for multiple β-lactams, such as ampicillin, amoxicillin–clavulanate, ampicillin–sulbactam, aztreonam, several cephalosporins, and carbapenems (Supplementary Table S5). Categorical S/I/R interpretation was applied only where CLSI provides *Stenotrophomonas maltophilia*-specific breakpoints, including levofloxacin and trimethoprim–sulfamethoxazole. For all other agents, inhibition-zone diameters, including 0-mm zones, were reported descriptively without categorical interpretation (Supplementary Table S5).

### Genome sequencing, assembly, and quality assessment of NG-SM01

De novo assembly yielded a closed, gap-free genome of 4,111,737 bp with 66.9% GC content, represented by a single contig (Table 1). Assembly integrity was supported by chromosome-scale Nx profiles, cumulative-length curves, and a unimodal GC distribution within the expected high-GC range for the lineage (Supplementary Figs. S2–S4). Raw ONT and Illumina reads were used for hybrid polishing, and quality was assessed using complementary completeness/contamination and taxonomic-screening approaches. BUSCO v5.7.1 (bacteria_odb10; *n* = 124) recovered 100.0% complete markers, all single-copy, with 0.0% duplicated, fragmented, or missing BUSCOs. CheckM2 independently estimated 100.0% completeness and 0.0% contamination (Table 1). Kraken2 classified the final assembly within the *Stenotrophomonas maltophilia* group (Xanthomonadaceae); any non-target contigs or fragments detected during screening, such as *Psychrobacter*, were excluded before annotation. Prokka predicted 3,636 CDS, 13 rRNA genes, 77 tRNA genes, and 1 tmRNA, while Tandem Repeat Finder detected 185 tandem repeats totaling 23,138 bp, corresponding to 0.563% of the genome (Table 1).

**Table 1:** Genome assembly, annotation, and quality metrics for the *Stenotrophomonas maltophilia* complex (Sgn4) isolate NG-SM01.

Feature	Value
Genome size (bp)	4,111,737
GC content (%)	66.9
Number of contigs (≥ 500 bp)	1
Largest contig (bp)	4,111,737
N50 (bp)	4,111,737
L50	1
Percent gaps (%)	0
Protein-coding sequences (CDS)	3,636
rRNA genes	13
tRNA genes	77
tmRNA genes	1
TRF repeats (count)	185
Total repeat length (bp)	23,138
Repeat density (% genome)	0.563
BUSCO completeness (%)	100.0 (S: 100.0; D: 0.0; F: 0.0; M: 0.0)
CheckM2 (completeness/contamination, %)	100.0 / 0.0

### Genome architecture and COG functional profile of NG-SM01

**Fig. 1:**
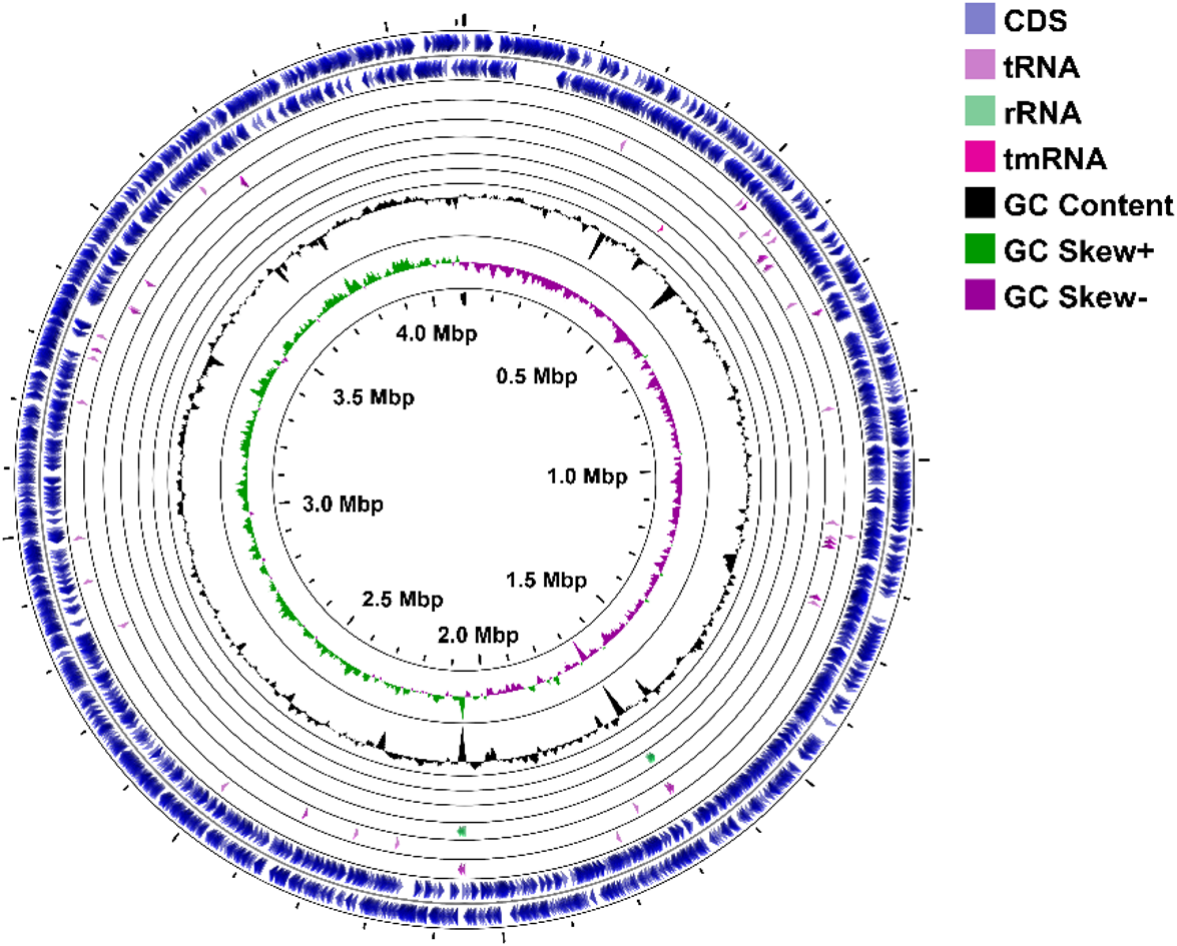
Circular genome map and architectural features of the * Stenotrophomonas maltophilia* complex Sgn4 isolate NG-SM01. Circular representation of the NG-SM01 chromosome showing annotated coding sequences (CDS) on the forward and reverse strands, tRNA, rRNA, and tmRNA genes, together with genome-wide GC content and GC skew. Coordinate ticks indicate chromosomal position in Mb.

The whole-genome circular map summarizes CDS/RNA feature distribution together with GC content and GC skew (Fig. 1). EggNOG-based COG profiling showed that a substantial fraction of the genome remained poorly characterized: category S (“function unknown”) comprised 738 genes (21.38%), and 228 genes (6.60%) lacked any COG assignment (Supplementary Fig. S5A). Among classified functions excluding category S, the most represented categories were amino acid transport and metabolism (E), cell wall/membrane/envelope biogenesis (M), transcription (K), and inorganic ion transport and metabolism (P), followed by signal transduction mechanisms (T), energy production and conversion (C), and translation/ribosomal structure and biogenesis (J) (Supplementary Fig. S5B). Overall, 1,581 proteins (43.47%) were annotated as hypothetical, whereas 2,055 (56.53%) received non-hypothetical functional annotations (Table 1).

### Phylogenomics and type-strain benchmarking place NG-SM01 within the *Stenotrophomonas maltophilia* complex

**Fig. 2:**
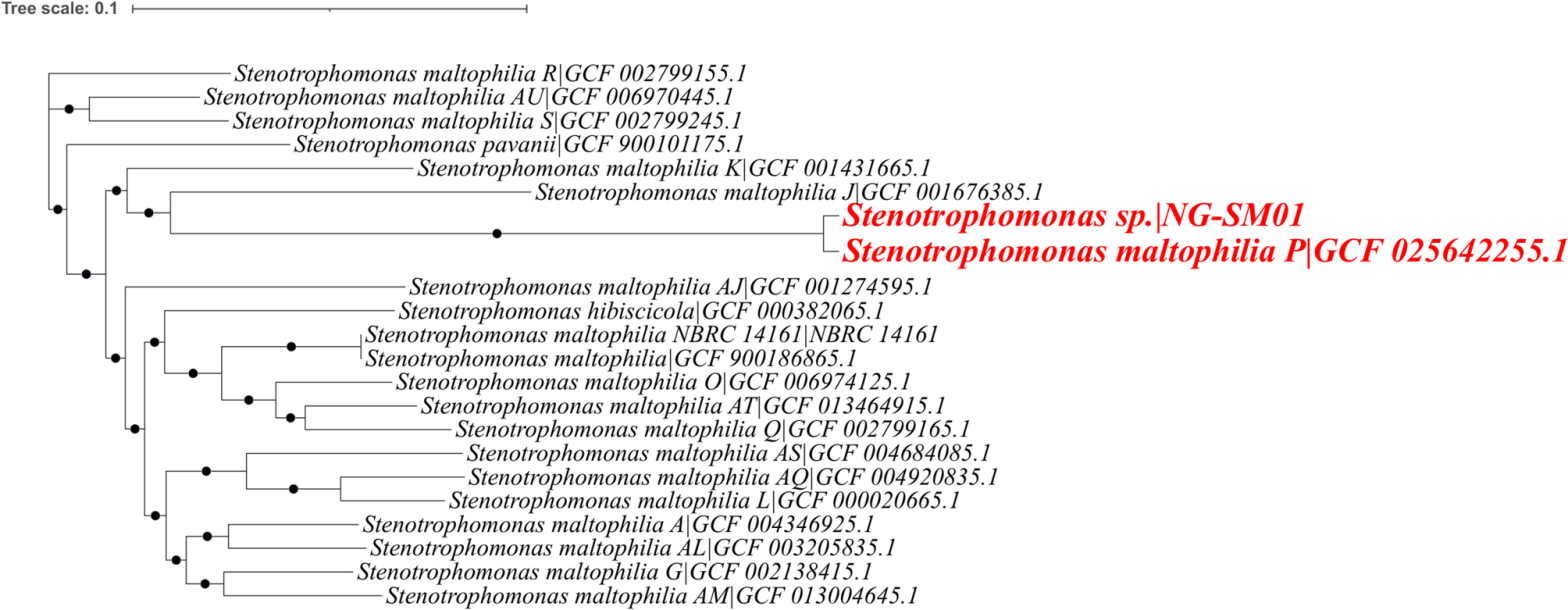
Core-genome phylogeny supports placement of NG-SM01 within the *Stenotrophomonas maltophilia* complex and separation from the NBRC 14,161 type-strain anchor. Maximum-likelihood tree inferred from the Panaroo core-gene alignment using IQ-TREE2. NG-SM01 formed a strongly supported sister lineage to the closest GTDB reference genome, GCF_025642255.1, whereas the *S. maltophilia* sensu stricto type strain NBRC 14,161 was positioned distantly relative to this clade. Small black circles on internal branches indicate nodes with 100% bootstrap support. The scale bar indicates substitutions per site; detailed node-support values are summarized in Supplementary Table S10.

For genome-resolved placement, we curated a comparative panel of the closest GTDB-linked *Stenotrophomonas* genomes (*n* = 18), together with two outgroups (*S. hibiscicola* and *S. pavanii*) and the *Stenotrophomonas maltophilia* type strain NBRC 14,161 as a nomenclatural anchor (Supplementary Table S6). All retained genomes met basic assembly-quality expectations based on genome length, GC content, and N50 (Supplementary Table S6), and showed high BUSCO completeness with minimal missing markers (Supplementary Table S7). FastANI source annotation indicated an environmentally dominated comparison set (14/19, 74%), with a smaller clinical subset (4/19) (Supplementary Table S8). Genome-wide nucleotide similarity placed NG-SM01 closest to GCF_025642255.1 (ANI 98.858%; alignment fraction 1309/1370, 95.55%) (Supplementary Table S8). In contrast, similarity to the type-strain anchor was markedly lower: NG-SM01 vs. NBRC 14,161 showed ANI 88.254% and alignment fraction 1086/1370 (79.27%), while GCF_025642255.1 vs. NBRC 14,161 showed ANI 88.270% (Supplementary Table S8). TYGS genome-to-genome comparisons were consistent with this separation, showing dDDH values below the 70% species threshold relative to NBRC 14,161: d4, 33.5% (CI 31.1–36.0); d0, 56.7% (CI 53.1–60.2); and d6, 50.6% (CI 47.5–53.6). These results support placement of NG-SM01 within the *S. maltophilia* complex but outside *S. maltophilia* sensu stricto as represented by the NBRC 14,161 type strain. As lower-resolution context, 16 S rRNA BLASTn matched multiple records annotated as *S. maltophilia* at approximately 99.1% identity, including records linked to NBRC 14,161 and ATCC 13,637 (Supplementary Table S9), highlighting the limited resolution of 16 S rRNA for this complex. Core-genome phylogeny based on the Panaroo core alignment and IQ-TREE2 recovered NG-SM01 as a sister lineage to GCF_025642255.1 with maximal support (UFboot = 100), with deep nodes supported by bootstrap values ≥ 99 (Fig. 2; Supplementary Table S10). Placement remained stable when the type-strain anchor was excluded (Supplementary Fig. S6). To position NG-SM01 within the established population structure of the *S. maltophilia* complex, we reconstructed a lineage-anchored core-genome phylogeny spanning the 23 defined lineages, including Sm1–Sm18, Sgn1–Sgn4, and Sm4a/Sm4b. In this framework, NG-SM01 clustered within the Sgn4 genomospecies and formed a strongly supported sister relationship with the Sgn4 reference (UFboot = 100) (Supplementary Fig. S7). Together, ANI, dDDH, and lineage-anchored phylogenomics support assignment of NG-SM01 to Sgn4 within the *S. maltophilia* complex, as a strongly supported sister lineage to GCF_025642255.1 and clearly separated from *S. maltophilia* sensu stricto anchored by NBRC 14,161 (Fig. 2; Supplementary Table S8; Supplementary Fig. S7).

### Multilocus sequence typing of NG-SM01 (PubMLST *Stenotrophomonas maltophilia* scheme)

**Fig. 3:**
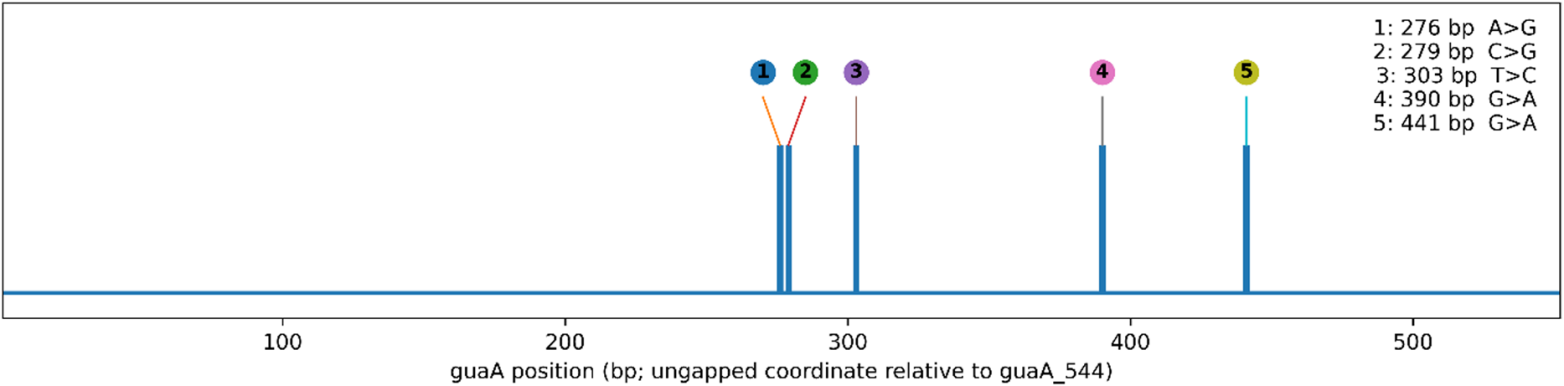
SNP-position map of the newly curated *guaA* MLST allele in NG-SM01 relative to PubMLST *guaA*(544). Vertical bars indicate the five SNPs that distinguish NG-SM01 from the closest PubMLST allele, *guaA*(544), across the 552-bp locus, using ungapped coordinates relative to *guaA*(544). Numbered markers correspond to the substitutions shown in the inset: 1, 276 bp (A > G); 2, 279 bp (C > G); 3, 303 bp (T > C); 4, 390 bp (G > A); and 5, 441 bp (G > A). Following PubMLST curator review, the NG-SM01 sequence was accepted as *guaA* allele 909, and the allelic profile was assigned ST1409.

MLST of the drinking-water isolate NG-SM01 using the PubMLST *Stenotrophomonas maltophilia* seven-locus scheme (*atpD*, *gapA*, *guaA*, *mutM*, *nuoD*, *ppsA*, and *recA*) yielded complete, unambiguous allele calls for six loci based on exact genome matches: *atpD*(14), *gapA*(108), *mutM*(96), *nuoD*(291), *ppsA*(250), and *recA*(222). In contrast, *guaA* initially showed no exact match in PubMLST; the closest allele was *guaA*(544), from which NG-SM01 differed by five SNPs across the 552-bp locus: 276 A > G, 279 C > G, 303 T > C, 390 G > A, and 441 G > A relative to the *guaA*(544) coordinate system (Fig. 3; Supplementary Table S11). Following PubMLST curator review, this sequence was accepted as the new *guaA* allele 909. The resulting allelic profile was assigned ST1409, and NG-SM01 was registered under PubMLST isolate ID 2048.

### Pangenome architecture and NG-SM01 panel-restricted gene blocks

Pangenome reconstruction across the 20-genome comparison set resolved 11,082 gene clusters and was accessory-dominated (77.95%). The pangenome comprised 2,444 core clusters (22.05%), 312 soft-core clusters (2.82%), 2,176 shell clusters (19.64%), and 6,150 cloud clusters (55.50%) (Table 2). Within this selected panel, 164 clusters were detected only in NG-SM01 (1/20 genomes; 1.48% of the pangenome), and these were concentrated in the cloud fraction. Most of these NG-SM01 panel-restricted clusters encoded hypothetical proteins (137/164; 83.5%), whereas 27/164 (16.5%) had non-hypothetical functional assignments (Table 2). Because these calls depend on the composition of the 20-genome comparison set, they are interpreted here as panel-restricted rather than globally unique features. Mapping the panel-restricted genes onto the NG-SM01 chromosome (edge_1) revealed a modular organization into 27 discrete blocks, spanning 117–46,832 bp and containing 1–54 panel-restricted genes per block (Supplementary Table S12). Blocks with the highest number of panel-restricted genes included block_23 (54 genes; all hypothetical), block_16 (17 genes), block_27 (16 genes), and block_4 (16 genes) (Supplementary Tables S12–S13). Non-hypothetical functions were concentrated mainly in block_27 (8 annotated genes), block_16 (6), and block_26 (4), with smaller contributions from block_14 and block_2 (2 annotated genes each), while all remaining blocks contained one or no annotated non-hypothetical gene (Supplementary Table S14). Functionally, block_14 contained carbohydrate/envelope-associated genes, including *algL* and a glycosyltransferase with COG G/M assignments. Block_26 included a transport–response module with MacAB-like efflux and signaling/regulatory proteins assigned to COG V/M/T. Block_27 contained an integrated regulatory–transport–metabolic cassette, including transcriptional regulators (COG K), MFS transport-associated functions (COG E/G/P), and catabolic enzymes such as an isochorismatase (COG Q), whereas block_23 remained entirely hypothetical (Supplementary Tables S15–S16). Block-level enrichment testing for non-hypothetical genes using one-sided Fisher’s exact test with Benjamini–Hochberg correction identified block_27 as the only statistically significant functional hotspot among the panel-restricted blocks: 8 non-hypothetical vs. 8 hypothetical genes; OR = 6.79; *P* = 0.00103; BH-FDR = 0.0279. Block_26 (4 vs. 3; OR = 7.77; *P* = 0.01466; BH-FDR = 0.1979) and block_16 (6 vs. 11; OR = 3.27; *P* = 0.03863; BH-FDR = 0.3477) showed enrichment trends but were not significant after FDR correction (Supplementary Tables S17–S18). Together, these results identify block_27 as the most functionally interpretable panel-restricted block within the selected comparison set, with block_16 and block_26 retained as additional candidate regions requiring cautious interpretation.

**Table 2:** Pangenome composition and NG-SM01 panel-restricted clusters within the 20-genome panel. Distribution of gene clusters across pangenome frequency classes, including core, soft-core, shell, and cloud fractions, in the 20-genome comparative panel. The table also summarizes the subset detected only in NG-SM01 within this panel (1/20 genomes), with annotation breakdown into hypothetical and non-hypothetical proteins.

Category	Presence frequency across genomes	Gene clusters (*n*)	% of total pangenome
Core	99–100%	2444	22.05
Soft-core	95–<99%	312	2.82
Shell	15–<95%	2176	19.64
Cloud	0–<15%	6150	55.5
Accessory genome (Soft-core + Shell + Cloud)	< 99%	8638	77.95
Total pangenome	0–100%	11,082	100
Panel-restricted to NG-SM01 (subset of Cloud)	Present in 1/20 genomes (5.0%)	164	1.48
└─ Hypothetical proteins (panel-restricted)	Present in 1/20 genomes (5.0%)	137	1.24
└─ Non-hypothetical/annotated (panel-restricted)	Present in 1/20 genomes (5.0%)	27	0.24

### Pangenome dynamics in the NG-SM01–centered panel

We evaluated pangenome expansion within the 20-genome NG-SM01-centered comparison set. Rarefaction across 500 random genome-order permutations showed continued pangenome growth without saturation, reaching a mean of 11,082 gene clusters at 20 genomes (Supplementary Fig. S8; Supplementary Table S19). The gene-discovery curve remained non-zero throughout, with the final genome addition introducing an average of 229.65 ± 121.80 clusters, consistent with a persistent low-frequency accessory fraction in this dataset (Supplementary Fig. S9; Supplementary Table S19). Heaps’ law modeling supported an open-pangenome pattern within the sampled panel, with α = 0.3479 at 20 genomes (95% CI 0.3081–0.3814), below the α = 1 closure threshold (Supplementary Table S20). This pattern was stable across sampling depths from 8 to 20 genomes, with α values remaining below 1 (Supplementary Fig. S10; Supplementary Table S20). Together, these analyses support an open-pangenome pattern within this selected *Stenotrophomonas maltophilia* complex comparison set, while recognizing that the estimate is conditional on panel size, genome selection, and taxonomic composition.

### Carbohydrate-active enzyme landscape and locus-level remodeling in the NG-SM01–centered *Stenotrophomonas maltophilia* complex panel

**Fig. 4:**
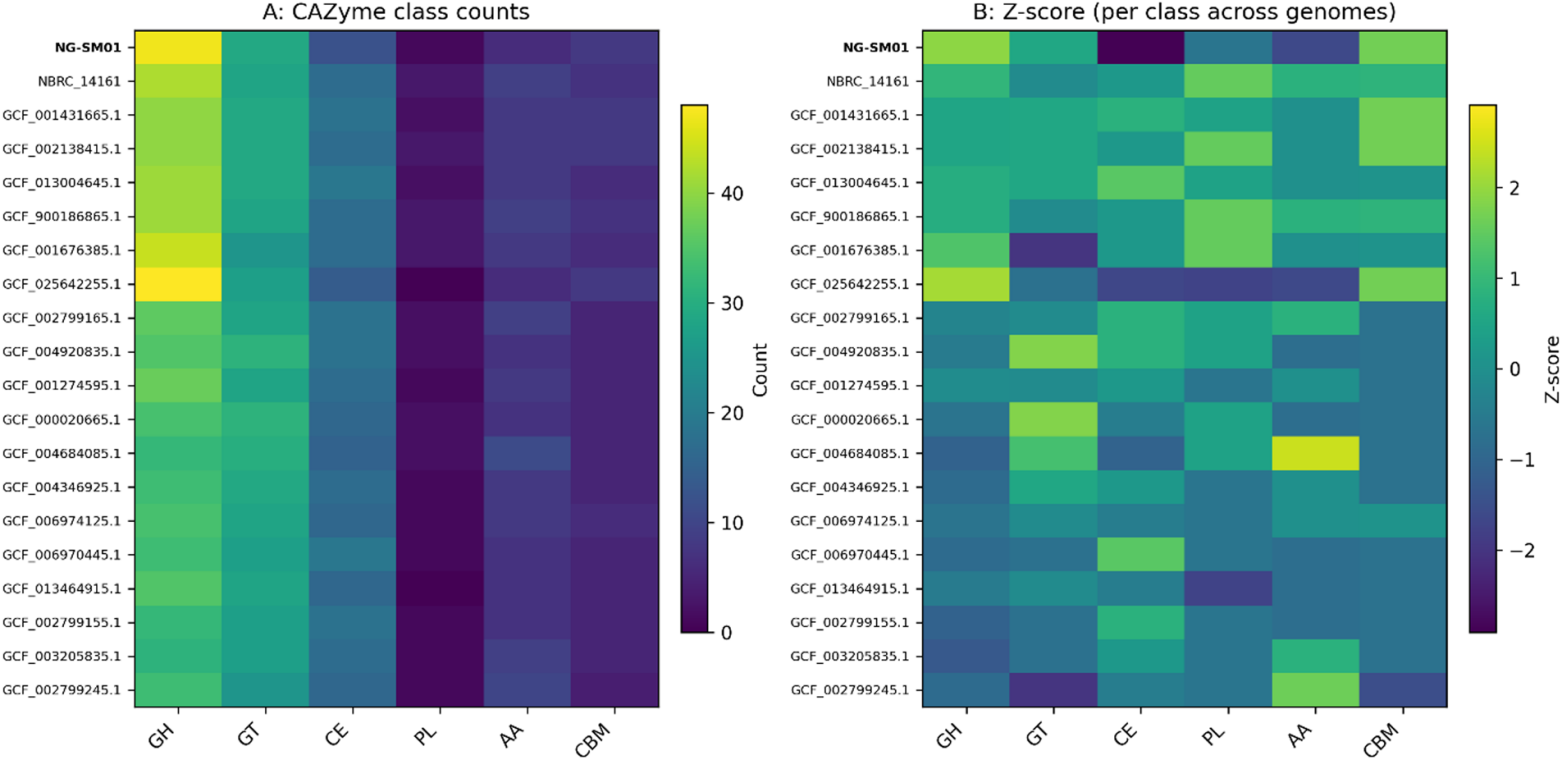
CAZyme class profiles across the NG-SM01-centered Stenotrophomonas maltophilia complex panel. (**A**) Heatmap of absolute CAZyme copy numbers per genome for the six CAZy classes: GH, GT, CE, PL, AA, and CBM, summarizing class-level abundance across the 20-genome panel. (**B**) Z-score-normalized heatmap per class across genomes, highlighting relative over- or under-representation independent of absolute magnitude. Together, the panels show a broadly conserved CAZyme class architecture with panel-relative shifts in NG-SM01, including GH/CBM-weighted composition and lower CE/AA representation.

Across the 20-genome panel, carbohydrate-active enzyme (CAZyme) profiles were broadly conserved but varied in class balance (Supplementary Table S21). Total CAZyme counts ranged from 89 to 106 per genome, with glycoside hydrolases (GH) and glycosyltransferases (GT) forming the dominant classes, followed by carbohydrate esterases (CE) (Supplementary Tables S21–S22). These class-level patterns are summarized as absolute counts and Z-score-normalized profiles in Fig. 4 A–B. Within this panel, NG-SM01 carried 103 predicted CAZymes and showed a relative shift toward GH and carbohydrate-binding module (CBM) representation, together with lower CE and auxiliary activity (AA) fractions compared with several reference genomes (Supplementary Table S21; Fig. 4 A–B). This pattern suggests panel-relative class reweighting rather than an increased total CAZyme load. Family-level variation was driven mainly by low-abundance, patchily distributed CAZyme families, including selected CBM, PL, and AA subfamilies (Supplementary Fig. S11). Among the 27 non-hypothetical genes detected only in NG-SM01 within the 20-genome panel, two encoded CAZy-domain proteins: NG_SM01_02052 (*algL*), with a partial-coverage PL5 match, and NG_SM01_02055, with an intact GT26 glycosyltransferase domain. GT26 was infrequent within this panel, occurring in NG-SM01 and GCF_004920835.1. Both genomes shared a conserved glycan-associated backbone, including *wzc* and *ugd*; however, within the corresponding locus, NG-SM01 carried *algL*/PL5 adjacent to the GT26-associated region, whereas the comparator carried a GDP-sugar modification submodule (Supplementary Table S23). Because the comparator also encoded an alginate lyase and a short-chain fatty-acid transporter elsewhere in the genome, these differences are best interpreted as locus-level reconfiguration within the sampled panel rather than simple gain or loss. Overall, NG-SM01 showed panel-relative CAZyme class reweighting and a remodeled GT26-linked glycan locus containing the only CAZy-annotated genes among the 27 non-hypothetical panel-restricted features (Supplementary Tables S21–S23; Fig. 4; Supplementary Fig. S11).

#### Genome-wide genomic-island architecture and validation

Genomic-island (GI) prediction on edge_1 returned 20 raw intervals supported by at least one caller, which were merged into 18 non-redundant GIs ranging from 4.2 to 26.0 kb (Supplementary Table S24). Composition profiling of five prioritized islands, GI_3, GI_4, GI_6, GI_16, and GI_17, revealed variable discordance relative to the chromosome-wide GC content of 66.9%. GI_4 and GI_6 showed the greatest GC depletion, with GI_4 measuring 25,999 bp, 59.58% GC, and ΔGC − 7.32, and GI_6 measuring 11,900 bp, 60.36% GC, and ΔGC − 6.54. GI_3 and GI_17 showed intermediate GC depletion, with ΔGC values of − 4.20 and − 3.99, respectively, whereas GI_16 was comparatively host-concordant, with 66.58% GC and ΔGC − 0.32 (Supplementary Table S25). Tetranucleotide/codon-usage deviations and CDS-level amelioration metrics, including dinucleotide signature distance, codon-usage JSD, CpG/ApT odds, and ENC, showed a similar ranking, identifying GI_4 as the most compositionally discordant region and GI_16 as the most host-like among the prioritized islands. Sliding-window GC% and GC-skew profiles across each GI and ± 10 kb flanking regions showed transitions corresponding to the predicted GI coordinates (Supplementary Tables S26–S28; Supplementary Fig. S12). Boundary-context analyses provided additional support for insertion-like organization at several loci. Border-gene mapping defined discrete flanking regions and coherent internal gene sets (Supplementary Tables S29–S30). GI_3 showed the strongest mobility-associated footprint, with tmRNA/ssrA in the left neighborhood, a prophage-type integrase IntA (NG_SM01_00373) near the island, and a compact stabilization/mobility-associated cassette centered on PasT and IntA (Supplementary Tables S31–S32). Repeat-based breakpoint searches detected short target-site-duplication-like signals at GI_6, consisting of a 10-bp motif with one mismatch, and at GI_17, consisting of an exact 9-bp repeat with an additional nearby near-repeat. A focused 200-bp flank scan yielded stronger TSD-like support for GI_17, weaker motif-consistent support for GI_6, and no detectable direct repeats for GI_3, GI_4, or GI_16 (Supplementary Tables S33 and S36). Although GI_4 lacked strict edge hallmarks within the merged interval, a broader neighborhood scan placed it in a dual tRNA-rich context, with tRNA-Arg(acg) upstream and tRNA-Ser(gct) near the right-side region, consistent with an integration-permissive genomic neighborhood rather than direct evidence of active mobility (Supplementary Tables S28–S29). Illumina read mapping supported assembly continuity across the boundaries of all five prioritized islands. Coverage was comparable to flanking regions, with GI-to-flank mean-depth ratios ranging from 0.986 to 1.174, and no boundary-associated coverage collapses were observed (Supplementary Table S37A). Depth traces across ± 10 kb regions were stable (Supplementary Fig. S13), and junction-spanning reads were detected at both boundaries, with 272–451 reads spanning left boundaries and 307–488 reads spanning right boundaries (Supplementary Table S37B). These results support the structural integrity of the predicted regions in the final assembly, although they do not by themselves demonstrate recent mobility, excision, or functional activity. Comparative conservation separated the prioritized loci into different classes. At the nucleotide level, only GI_16 and GI_3 matched the curated panel above the defined threshold: GI_16 showed a full-length, high-identity hit to GCF_025642255.1 (pid 99.17%, qcov 100%), whereas GI_3 matched GCF_001676385.1 with high identity but partial coverage (pid 96.3%, qcov 65%) (Supplementary Table S38). GI_4, GI_6, and GI_17 had no curated-panel nucleotide hits above threshold. Protein-level conservation supported this distinction: GI_16 showed complete reciprocal-best-hit recovery (12/12 CDS) and preserved adjacency (11/11; rate 1.0), whereas GI_3 retained reciprocal-best-hit matches for most CDS (7/9) but lacked conserved adjacency (0/4; rate 0.0), consistent with structural remodeling of a mobile-element-associated cassette (Supplementary Table S39). Panel-wide prevalence was assessed using a three-state scheme: PRESENT, identity ≥ 90% and qcov ≥ 70%; PARTIAL, identity ≥ 90% and qcov 10–69%; and ABSENT otherwise. GI_16 was the only prioritized island recovered as a full-length block in the reference panel (PRESENT 1/19; 5.3%). GI_6 and GI_17 were absent from all 19 reference genomes under this scheme (0/19 PRESENT; 0/19 PARTIAL). GI_3 and GI_4 were never fully present (0/19 PRESENT), but showed fragment sharing, with GI_3 classified as PARTIAL in 5/19 genomes (26.3%) and GI_4 classified as PARTIAL in 17/19 genomes (89.5%) (Supplementary Table S40; Supplementary Fig. S14). Synteny comparisons against best-matching reference windows supported local conservation of shared segments within divergent genomic neighborhoods (Supplementary Figs. S15A–E). Collectively, compositional discordance, boundary-context evidence, read-mapping support, and panel-wide conservation patterns prioritized GI_4, GI_6, and GI_17 as candidate panel-restricted acquired regions. GI_4 and GI_6 showed the strongest compositional discordance, GI_6 and GI_17 were absent from the selected reference panel under the applied thresholds, GI_16 appeared comparatively host-concordant, and GI_3 was consistent with a stabilized but structurally remodeled mobile-element-associated cassette. These classifications should be interpreted as genomic evidence for candidate acquired architecture, not as direct evidence of current mobility, excision, or experimentally confirmed function.

### Functional resolution of the two most host-discordant islands identifies candidate EPS-remodeling and SOS-associated stress-response loci

**Fig. 5:**
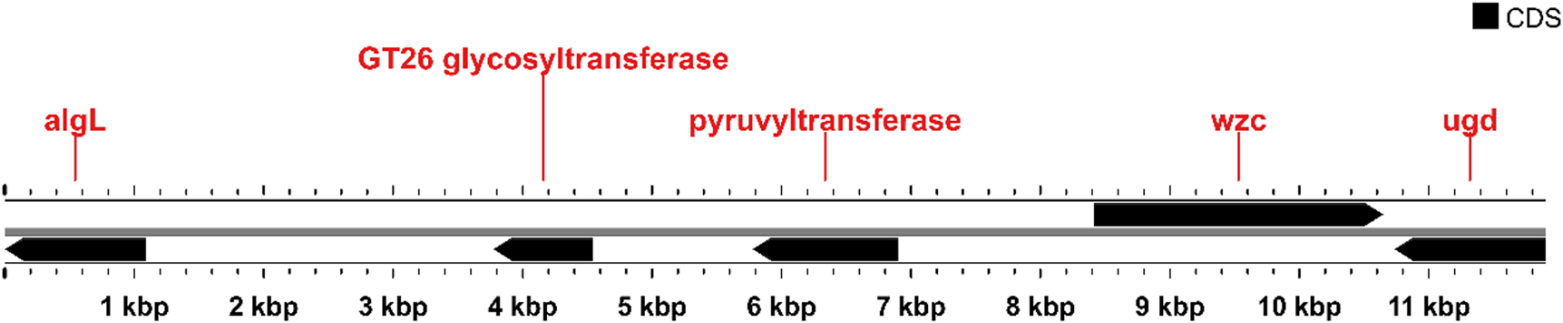
Gene organization of GI_6, a candidate EPS/envelope-remodeling locus. Focused gene-neighborhood map of GI_6 showing the panel-restricted *algL* locus, a GT26-family glycosyltransferase, a putative pyruvyltransferase, and capsule/EPS-associated backbone genes including *wzc* and *ugd*. Coding sequences are shown as arrows indicating transcriptional orientation.

**Fig. 6:**
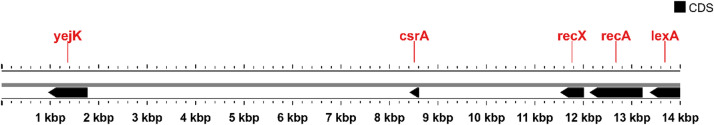
Gene organization of GI_4, a candidate SOS/IME-associated locus. Focused gene-neighborhood map of the highlighted GI_4 region showing the NG-SM01 panel-restricted gene *yejK*, the stress-linked regulator *csrA*, and the adjacent *recX–recA–lexA* SOS-associated regulatory/repair neighborhood. Coding sequences are shown as arrows indicating transcriptional orientation.

To explore the functional content of the two most compositionally discordant islands, GI_6 (11.9 kb) and GI_4 (26.0 kb) were resolved using orthology-supported annotation and protein-domain evidence from EggNOG and InterProScan. This analysis identified two candidate modules relevant to surface-associated growth and oxidative-stress response hypotheses: an EPS/envelope-remodeling locus in GI_6 and an SOS-associated regulatory/repair neighborhood in GI_4. GI_6 encoded a compact polysaccharide-remodeling locus containing AlgL (PL5 alginate lyase; NG_SM01_02052), a WecB/TagA/CpsF-family glycosyltransferase with a GT26 domain (NG_SM01_02055), conserved capsule/EPS-associated backbone genes including *wzc* and *ugd*, and a putative pyruvyltransferase consistent with possible polymer modification (Fig. 5). Within the 19-genome comparative panel, *algL* and NG_SM01_02055 were detected only in NG-SM01 and are therefore interpreted as panel-restricted accessory genes. Remote BLASTp searches against NCBI nr recovered three near-identical full-length homologs of NG_SM01_02055 within the *S. maltophilia* complex, indicating that the relevant distinction is the island-localized cassette architecture rather than global rarity of the enzyme sequence. Across the selected comparative panel, GI_6 was absent under the applied thresholds (0/19 PRESENT; 0/19 PARTIAL), supporting its classification as a panel-restricted EPS/envelope-associated candidate region. GI_4 contained SOS-associated regulatory and repair genes, including *recA*, *recX*, and *lexA*, together with *yejK*, which was panel-restricted in this comparison, and the stress-linked regulator *csrA* (Fig. 6). GI_4 was never recovered as a full-length island in the reference panel (0/19 PRESENT), but it showed extensive fragment sharing (17/19 PARTIAL; 89.5%). This pattern suggests that some SOS-associated submodules are broadly shared, whereas the local GI_4 architecture in NG-SM01 is structurally divergent. COG profiles further supported functional contrast between the two islands: GI_4 was enriched for replication/recombination/repair and information-processing categories (L/J/K), with a minor defense component (V), whereas GI_6 was enriched for envelope biogenesis (M), carbohydrate-linked functions (G), and trafficking/transport signatures (U) (Supplementary Table S41). Together, GI_4 and GI_6 provide genomic evidence for two candidate functional themes in NG-SM01: SOS-associated regulation/repair and EPS/envelope remodeling. These findings support a hypothesis-generating model in which stress-response circuitry and surface-matrix remodeling may be relevant to persistence-related traits. However, direct functional causality between GI_4 or GI_6 and the observed phenotypes was not demonstrated, and confirmation will require targeted approaches such as transcriptional analysis, mutant-based validation, and exposure assays under DWDS-mimetic disinfectant conditions.

### Mobilome architecture of NG-SM01

#### Prophage landscape

Prophage screening detected three loci in NG-SM01: R2 and R3, classified as intact or near-intact, and R1, classified as an incomplete remnant. R1 overlapped genomic island 3 (GI_3), suggesting a prophage-associated component within this region’s architecture. R2 (26.5 kb; edge_1:3,343,826–3,370,334) was annotated by Pharokka as a tailed double-stranded DNA (dsDNA) phage cassette containing morphogenesis, lysis, DNA-processing, and regulatory functions; however, no lysogeny-control module, such as an integrase, repressor, or excisionase, was annotated within the extracted boundaries despite structural completeness (Supplementary Table S42). R3 (10.5 kb; edge_1:4,088,887–4,099,432) formed a compact prophage-derived unit enriched for replication and head-associated functions but lacked a complete tail/lysis complement (Supplementary Table S43). R1 (10.7 kb; edge_1:426,303–437,064) showed a weak phage signal among mostly hypothetical CDS and overlapped GI_3; it encoded a prophage-type integrase, IntA (NG_SM01_00373; 435,823–437,064), consistent with a phage-associated insertion signature in this locus (Supplementary Tables S42–S43).

### IME-like element in GI_4

ICEberg 3.0 detected no complete integrative and conjugative element (ICE), as no evident mating-pair formation or type IV secretion system (T4SS) module was identified. However, ICEberg identified a high-confidence integrative and mobilizable element (IME)-like region within GI_4. This region spans edge_1:533,895–553,050 (19,156 bp; 57.9% GC), is bounded by attL/attR direct repeats, and is located adjacent to tRNA-Ser (NG_SM01_00484). It encodes a MOBQ-family relaxase (NG_SM01_00466), consistent with a potentially mobilizable but not self-transmissible element, and carries phage-type integrases (NG_SM01_00473 and NG_SM01_00483), supporting an IME-like architectural assignment (Supplementary Table S44). Most CDS within the region were hypothetical. The presence of *yejK* (NG_SM01_00475) and *csrA* (NG_SM01_00485), together with proximity to the *recX–recA–lexA* neighborhood (NG_SM01_00487–NG_SM01_00489), indicates that this IME-like region lies near SOS-associated regulatory and repair genes (Supplementary Table S44). However, active excision, stress-induced mobilization, or transcriptional coupling to the SOS response was not experimentally demonstrated.

### IS remnants

ISfinder detected 33 insertion sequence (IS) family matches spanning Tn3, IS110, IS481, ISNCY, IS1634, IS21, IS630, IS91, IS3, and IS4 families. Most hits were very short high-identity fragments, classified as MICROTRACE/TRACE and typically 22–57 bp, with additional fragments up to 177 bp, rather than intact IS elements (Supplementary Table S45). Only a small subset reached ≥ 200 bp, dominated by Tn3-family ISAzs17 signals: 339 bp, bitscore 204, E = 9 × 10⁻⁴⁹; 200 bp, bitscore 87.7, E = 1 × 10⁻¹³; and 290 bp, bitscore 83.8, E = 2 × 10⁻¹² (Supplementary Table S45). Other families were supported only by micro-fragments, such as ISRe46 from the IS481 family (34 bp; E = 0.008) and ISArsp14 from the ISNCY family (41 bp; E = 1 × 10⁻⁴) (Supplementary Table S45). Intersecting IS hits with GI coordinates for GI_3, GI_4, and GI_6 and with ± 200 bp boundary windows yielded no overlaps. No overlaps were observed with prophage regions R1, R2, or R3, indicating that these prioritized islands and prophage regions were not detectably flanked by IS-associated sequences under the current detection criteria (Supplementary Table S45).

### Lack of integrons, plasmids, and CRISPR–Cas

IntegronFinder detected no integrons, and PlasmidFinder detected no plasmid replicons, consistent with a primarily chromosomal mobilome under the applied screening criteria. Conservative CRISPR callers, including CRT and MinCED, detected no CRISPR arrays. Although a web-based scan reported three candidate CRISPR loci at positions 385,689–385,798; 2,208,594–2,208,685; and 2,373,742–2,373,826, each was ultra-short, 84–109 bp, and contained a single spacer with evidence level 1. Sequence inspection was more consistent with simple repeat duplications or short tandem-repeat motifs than with multi-spacer CRISPR arrays, and no *cas* genes were annotated. Collectively, NG-SM01 showed no detectable integrons, plasmid replicons, or functional CRISPR–Cas system under the applied screening criteria, consistent with a predominantly chromosomal mobilome containing candidate prophage- and IME-associated regions.

### Virulence and intrinsic resistome profile of NG-SM01

#### Limited VFDB virulence signal dominated by an adherence/motility locus

Screening against the Virulence Factor Database (VFDB) identified a single adherence-associated match: the type IV pili/twitching motility component PilG (VFG001225) at edge_1:3,017,819–3,018,154 (+), with 80.71% identity and 82.11% coverage (Supplementary Table S39). This result is consistent with surface-associated motility or attachment potential, rather than evidence of an expanded canonical virulence repertoire in this water-derived isolate.

### Efflux-forward intrinsic resistome with structured β-lactamase allele diversity

Resistance profiling identified an intrinsic resistome dominated by efflux-associated systems and aminoglycoside resistance determinants. ResFinder detected *aph(3')-IIc* (99.88% coverage; 84.77% identity), *aac(6')-Iz* (97.19% coverage; 84.63% identity), and the intrinsic metallo-β-lactamase *blaL1* (89.89% coverage; 82.40% identity) (Supplementary Table S39). CARD independently identified the SmeDEF resistance–nodulation–division (RND) efflux complex, including *smeD*, *smeE*, and *smeF* (96.79–99.75% coverage; 87.68–93.18% identity), and supported *aac(6')-Iz* as a chromosomal aminoglycoside acetyltransferase (Supplementary Table S46). AMRFinderPlus further identified an EmrABC major facilitator superfamily (MFS)-type system, including *emrB*, *emrA*, and *emrC*, each with 100% coverage and 92.76–96.59% identity, and corroborated *smeF* and *aac(6')-Iz* at high identity and coverage (Supplementary Table S46). To examine intrinsic β-lactamase diversity, we assessed *blaL1* allelic structure across NG-SM01 and the 19 reference genomes. Although nucleotide screening against the ResFinder *blaL1_3* reference (EF126059) produced partial identity/coverage, protein-level analysis supported an intact 261-aa ORF with 82.76% amino-acid identity to the BlaL1_3 protein, consistent with a divergent *blaL1*-like allele. Comparative proteome screening showed that NG-SM01 BlaL1 was identical to the homolog in GCF_025642255.1 across 261/261 aa, whereas the remaining 18 references formed a more divergent cluster, with approximately 80.84–83.14% identity and 100% query coverage (Supplementary Table S47). In addition, BLASTp identified a *blaL2*-like β-lactamase in NG-SM01, NG_SM01_02683 at edge_1:2,964,480–2,965,391 (+), matching the canonical L2 reference UniProt P96465 at 80.86% identity with 100% coverage (Supplementary Table S39). Collectively, these results support the presence of an efflux-associated intrinsic resistome, aminoglycoside-modifying enzyme homologs, and divergent intrinsic β-lactamase alleles in NG-SM01, including a *blaL1*-like allele shared with its closest reference, GCF_025642255.1.

### BacMet-guided screening identifies candidate metal/biocide tolerance-associated loci among panel-restricted genes

**Fig. 7:**
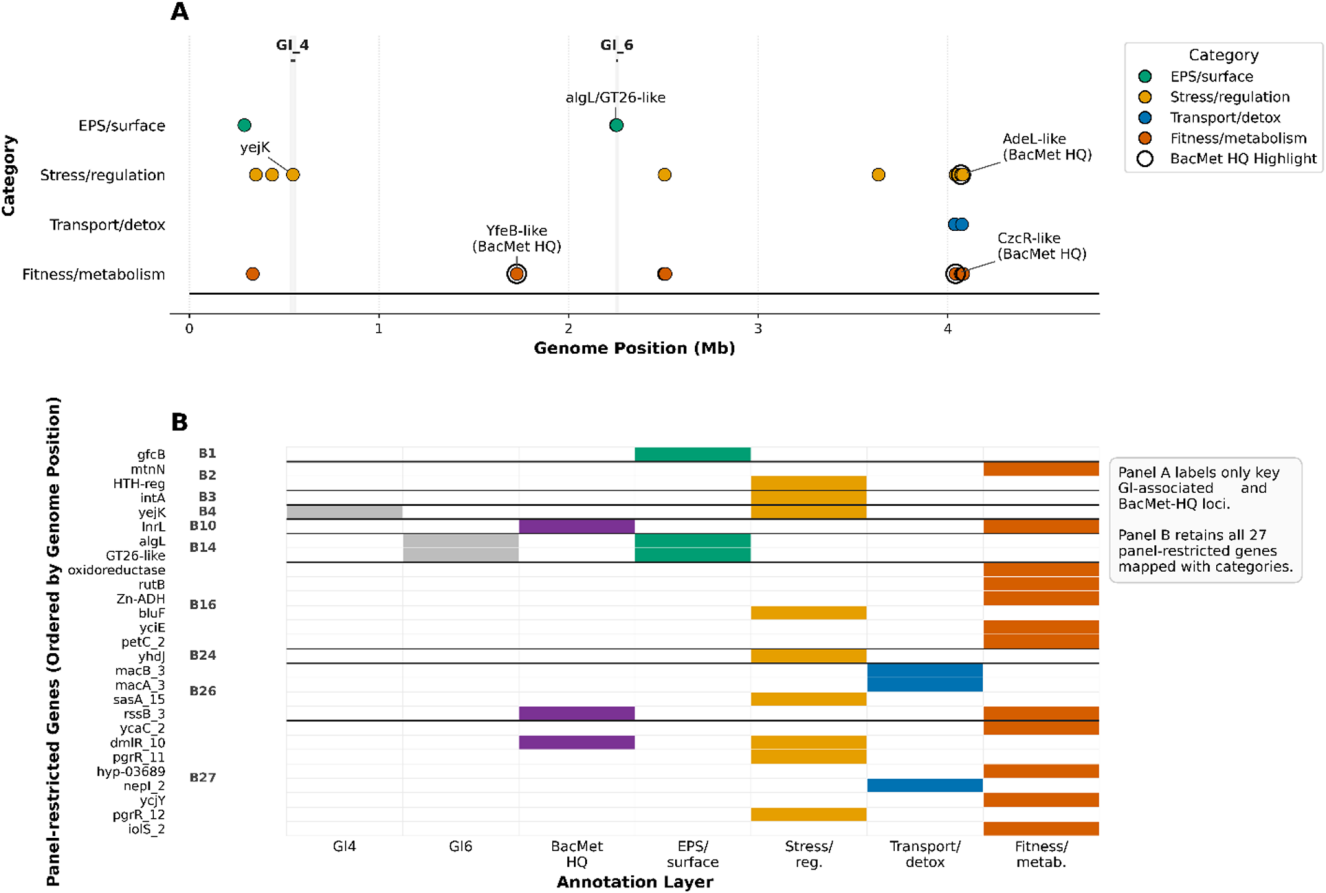
Distribution and supporting evidence for 27 panel-restricted annotated genes in NG-SM01. (**A**) Chromosomal positions of the 27 functionally annotated genes detected only in NG-SM01 within the 19-reference comparative set, colored by functional category. GI_4 and GI_6 are indicated as vertical gray bands. Labeled candidates include *yejK*, *algL*/GT26-like glycosyltransferase, and BacMet high-quality loci with YfeB-like, CzcR-like, and AdeL-like annotations. (**B**) Evidence-layer summary for all 27 annotated panel-restricted genes, ordered by genomic position. The labels B1–B27 on the y-axis indicate the corresponding panel-restricted gene blocks. Colored cells indicate genomic-island association, BacMet high-quality evidence, and functional-category assignment. Panel A labels only key GI-associated and BacMet-supported loci, whereas Panel B retains all 27 annotated panel-restricted genes.

To examine whether panel-restricted annotated genes were associated with tolerance-related functions, we focused on the 27 functionally annotated genes detected only in NG-SM01 within the 19-reference comparison set (Supplementary Tables S15–S16). These loci were concentrated in hotspot blocks, particularly blocks 14, 16, 26, and 27, suggesting modular organization within the selected panel rather than dispersed single-gene differences. EggNOG/COG assignments grouped this panel-restricted annotated set into three broad functional categories: envelope or extracellular polymeric substance (EPS)-associated remodeling, including *algL* and a GT26-like glycosyltransferase within genomic island 6 (GI_6); regulation/stress-associated functions, including HTH/LysR-type regulators and *YejK* within genomic island 4 (GI_4); and transport or maintenance-associated functions, including efflux/transport proteins and metabolic enzymes concentrated in hotspot blocks. These annotations suggest candidate functions that may be relevant to environmental persistence, but they do not establish functional causality. BacMet screening provided additional support for a focused subset of metal/biocide tolerance-associated candidates (Supplementary Tables S48–S49). Three loci met Tier 1 criteria: NG_SM01_03661, encoding a CzcR-like metal-stress regulator with qcov approximately 99% and bitscore 135; NG_SM01_03684, encoding an AdeL-like LysR regulator with qcov approximately 95% and bitscore 125; and NG_SM01_01595, encoding a YfeB-like metal/iron transport component with qcov approximately 93% and bitscore 87.8. NG_SM01_03660, annotated as a PmrB/SilS-like sensor kinase, showed lower coverage of approximately 51% despite a good bitscore of approximately 85 and was therefore retained as a Tier 2 domain-level candidate rather than a full-length orthology call. Together, these screening results associate selected panel-restricted annotated genes with candidate envelope remodeling, stress regulation, transport/homeostasis, and metal/biocide tolerance-related functions. These signals complement the GI_4 and GI_6 genomic observations but should be interpreted as functional predictions requiring experimental validation rather than direct evidence of persistence circuitry (Figs. 7 A–B).

### Genome-scale metabolic reconstruction identifies candidate metabolic features compatible with oligotrophic drinking-water conditions

**Fig. 8:**
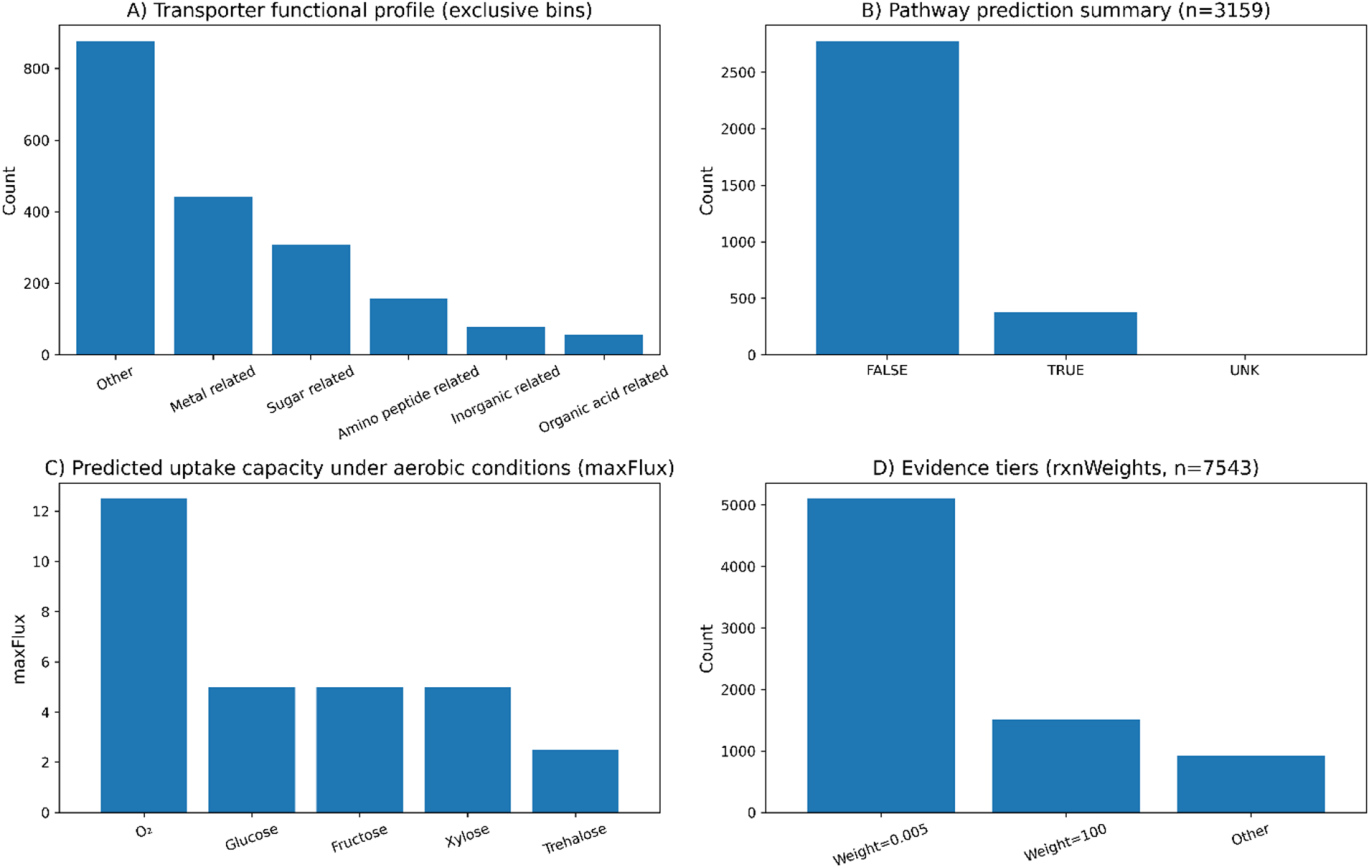
Genome-scale metabolic reconstruction summary for NG-SM01 using gapseq. (**A)** Predicted transporter classes, shown as mutually exclusive priority-assigned categories. (**B**) Distribution of pathway calls classified as TRUE, FALSE, or UNK. (**C**) Aerobic minimal-medium uptake constraints, shown as maxFlux values for O₂ and representative carbohydrates. (**D**) Distribution of reaction-weight evidence tiers, including high-evidence, penalized low-evidence, and intermediate categories.

To summarize the metabolic potential of NG-SM01, we reconstructed a genome-scale network using gapseq and examined transporter breadth, pathway support, minimal-medium requirements, and reaction-level evidence tiers (Fig. 8; Supplementary Tables S50–S52). The reconstruction recovered 3,159 pathway records and 1,920 transporter entries (Supplementary Table S50). Transport predictions spanned several functional classes, with “Other” representing the largest fraction (877/1,920; 45.7%), followed by metal-related transport (442/1,920; 23.0%), sugar transport (308/1,920; 16.0%), amino acid/peptide transport (158/1,920; 8.2%), inorganic ion transport (79/1,920; 4.1%), and organic acid transport (56/1,920; 2.9%) (Fig. 8A). The categorized transporter subset is listed in Supplementary Table S51, whereas “Other” represents uncategorized transporter predictions required to total 1,920 entries. NG-SM01 encoded several predicted nutrient-acquisition systems relevant to low-nutrient conditions, including a complete phosphate uptake module (*pstS/pstC/pstA/pstB* with *phoU*), iron-uptake-associated features such as *fiu* and *feoB*, and TonB-associated components including TonB-dependent receptor(s) and multiple *exbD* copies. Concordantly, the transporter list included 148 iron-associated and 34 phosphate-associated entries (Supplementary Table S50), supporting a broad predicted capacity for metal and nutrient acquisition. Among pathway calls, 380/3,159 (12.0%) were classified as TRUE, 2,776/3,159 (87.9%) as FALSE, and 3/3,159 (0.1%) as UNK (Fig. 8B; Supplementary Table S50). Minimal-medium inference required standard salts, ions, and trace components and supported an aerobic uptake profile, including predicted uptake of glucose, fructose, and xylose (each maxFlux 5.0) and trehalose (maxFlux 2.5) (Fig. 8C; Supplementary Table S52). The model mapped 13,134 reactions, all of which were gene-supported, and reaction evidence was stratified by gapseq weight classes, including 0.005 (5,103/7,543; 67.6%), 100 (1,515/7,543; 20.1%), and intermediate weights (925/7,543; 12.3%) (Fig. 8D; Supplementary Table S50). Together, these results support a broad predicted transporter repertoire and a genome-scale metabolic network compatible with aerobic growth and diverse nutrient uptake under low-nutrient conditions. These findings are consistent with metabolic flexibility that may be relevant in oligotrophic drinking-water environments, but they remain computational predictions and require experimental validation under defined nutrient-limiting conditions.

### Biofilm formation and comparative quantification by crystal violet assay

**Fig. 9:**
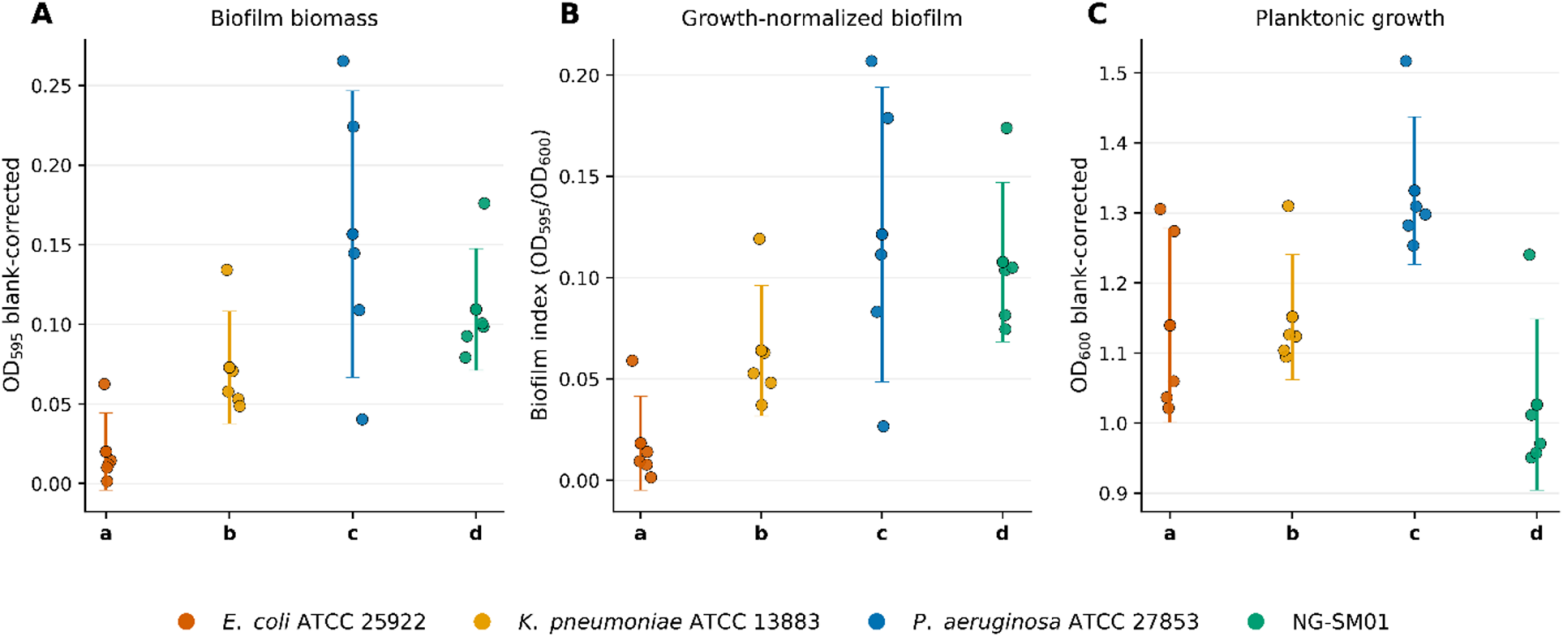
Quantification of biofilm formation by NG-SM01 and reference strains using the crystal violet microtiter assay. Biofilm formation under static 96-well microtiter conditions was assessed by measuring (**A**) blank-corrected crystal violet biomass (OD_595_corr), (**B**) growth-normalized biofilm index (OD_595_corr/OD_600_corr), and (**C**) blank-corrected planktonic growth (OD_600_corr) measured before washing. Blank correction was performed relative to medium-only control wells. Data points represent five independent biological replicates per strain (*n* = 5), with mean ± SD overlaid. Strain assignments: a, *E. coli* ATCC 25,922; b, *K. pneumoniae* ATCC 13,883; c, *P. aeruginosa* ATCC 27,853; d, NG-SM01.

Biofilm formation was quantified using a crystal violet (CV) microtiter assay with parallel measurement of planktonic growth (OD_600_) and CV-stained attached biomass (OD_595_). Across five independent biological replicates per strain, NG-SM01 produced a reproducible moderate biofilm signal after blank correction (OD_595_corr mean ± SD: 0.1094 ± 0.0382). This value was higher than the weak baseline observed for *E. coli* ATCC 25,922 (0.0201 ± 0.0242), but remained within the range observed for *P. aeruginosa* ATCC 27,853 (0.1567 ± 0.0899) and *K. pneumoniae* ATCC 13,883 (0.0729 ± 0.0353) under the same assay conditions (Fig. 9A; Supplementary Tables S53–S54). Growth normalization using OD_595_corr/OD_600_corr preserved the same general pattern, with NG-SM01 (0.1078 ± 0.0393) higher than *E. coli* (0.0184 ± 0.0232) and broadly comparable to *K. pneumoniae* (0.0641 ± 0.0322) and *P. aeruginosa* (0.1214 ± 0.0727) (Figs. 9B–C; Supplementary Tables S53–S54). Pairwise testing using Welch’s t-test with Holm correction confirmed significantly greater biofilm biomass for NG-SM01 than for *E. coli* for both OD_595_corr (Holm-adjusted *P* = 0.010; Hedges’ g = 2.52) and the growth-normalized index (Holm-adjusted *P* = 0.0116). In contrast, differences between NG-SM01 and either *K. pneumoniae* or *P. aeruginosa* were not significant under the tested conditions (Supplementary Tables S55–S56). OD_600_corr values were comparable across strains, indicating that the observed NG-SM01 biofilm signal was not attributable to increased planktonic growth alone (Fig. 9C). Overall, these data support reproducible moderate biofilm formation by NG-SM01 in the static microtiter assay, but do not indicate an exceptionally strong biofilm phenotype relative to all comparator strains.

### Oxidative-stress survival using an H₂O₂ challenge assay

**Fig. 10:**
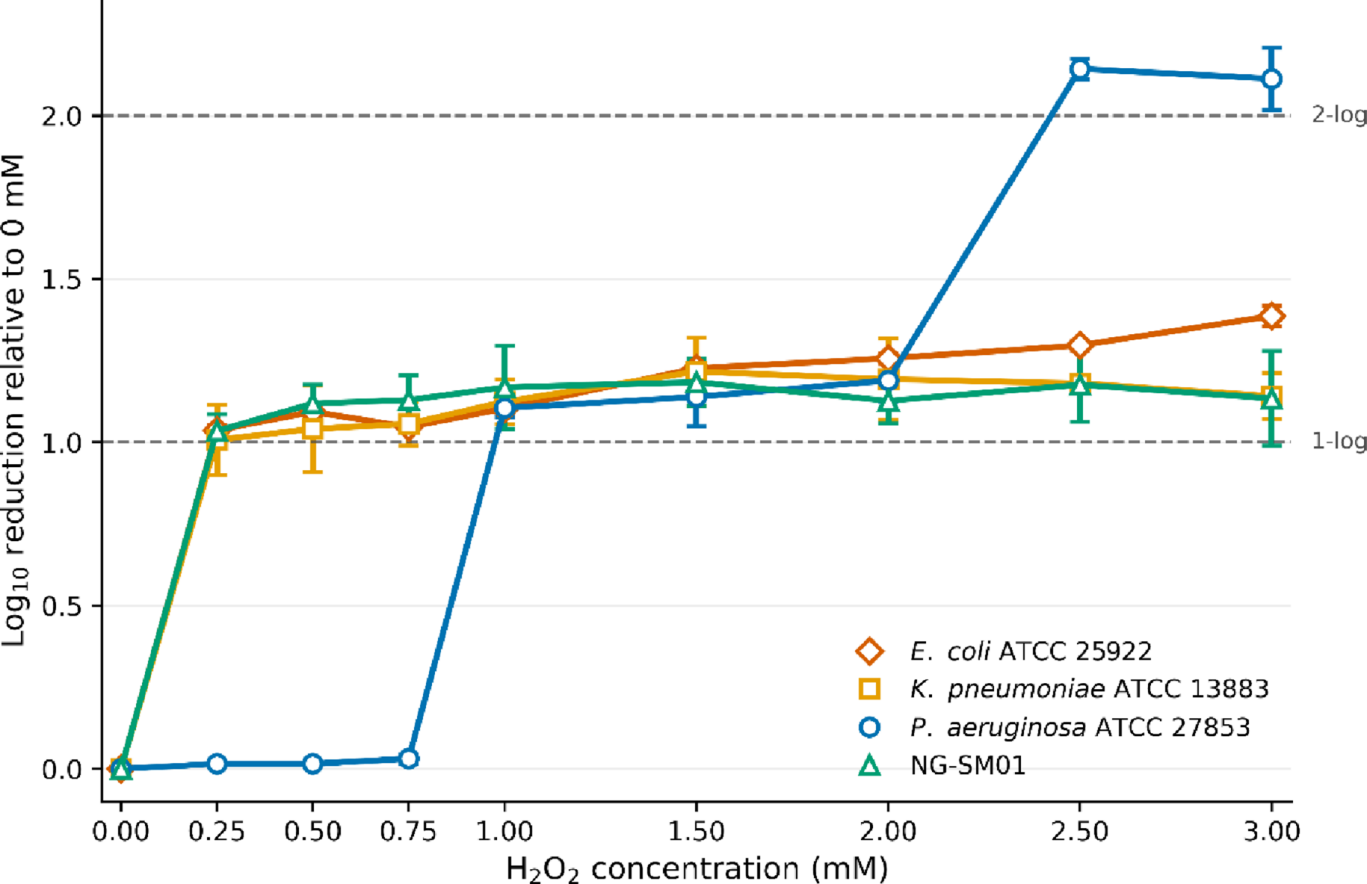
Acute H₂O₂ dose-response survival curves expressed as log_10_ reduction. Mid-log cultures (OD_600_ = 0.5) of NG-SM01, *P. aeruginosa* ATCC 27,853, *K. pneumoniae* ATCC 13,883, and *E. coli* ATCC 25,922 were exposed to H₂O₂ concentrations ranging from 0 to 3.0 mM for 5 min, quenched, and recovery-plated. Values represent log_10_ reduction relative to the matched 0 mM control (mean ± SD; *n* = 3 biological replicates). Dashed horizontal lines indicate 1-log and 2-log reduction reference thresholds. H₂O₂ was used as a laboratory oxidative-stress proxy and should not be interpreted as direct evidence of free-chlorine or chloramine tolerance.

To assess acute oxidative-stress survival, we quantified recovery following H₂O₂ exposure at 0–3.0 mM for 5 min using mid-log cultures standardized to OD_600_ = 0.5, with three biological replicates per condition. This assay was used as a general oxidative-stress proxy and was not intended to reproduce free-chlorine or chloramine exposure in DWDS. NG-SM01 was evaluated alongside *K. pneumoniae* ATCC 13,883 and *P. aeruginosa* ATCC 27,853, with *E. coli* ATCC 25,922 included as a more H₂O₂-sensitive laboratory comparator (Fig. 10; Supplementary Fig. S16). Absolute recoveries, expressed as log10 CFU/mL, are provided in Supplementary Table S57. H₂O₂ exposure produced distinct strain-associated killing patterns under the tested conditions (Fig. 10). *P. aeruginosa* showed a threshold-like profile, with minimal reductions at concentrations ≤ 0.75 mM followed by dose-dependent killing reaching approximately 2.1-log reduction at 2.5–3.0 mM. In contrast, NG-SM01 and *K. pneumoniae* showed an early approximately 1-log reduction by 0.25 mM, followed by a plateau from 1.0 to 3.0 mM, indicating limited additional killing across this concentration range. *E. coli* showed a similar early reduction but retained modest dose dependence at higher concentrations. Two-way ANOVA on log_10_ reductions confirmed significant effects of strain and dose and a significant strain × dose interaction: strain, F(3,72) = 35.17, *P* = 4.16 × 10⁻¹⁴; dose, F(8,72) = 485.30, *P* = 2.03 × 10⁻⁵⁹; interaction, F(24,72) = 90.29, *P* = 2.24 × 10⁻⁴⁴ (Supplementary Table S59). Linear trend analysis across 0–3.0 mM detected significant positive dose slopes for *P. aeruginosa* (+ 0.604 log10/mM, *P* = 1.60 × 10⁻⁵) and *E. coli* (+ 0.123 log10/mM, *P* = 8.45 × 10⁻⁷), whereas NG-SM01 and *K. pneumoniae* showed no significant trend across this range (*P* = 0.68 and *P* = 0.988, respectively; Supplementary Table S58). These results support a plateau-like response pattern for NG-SM01 during acute H₂O₂ exposure, but they should not be interpreted as direct evidence of tolerance to DWDS disinfectants such as free chlorine or chloramine.

### Time-kill kinetics under 2.0 mM H₂O₂ challenge

**Fig. 11:**
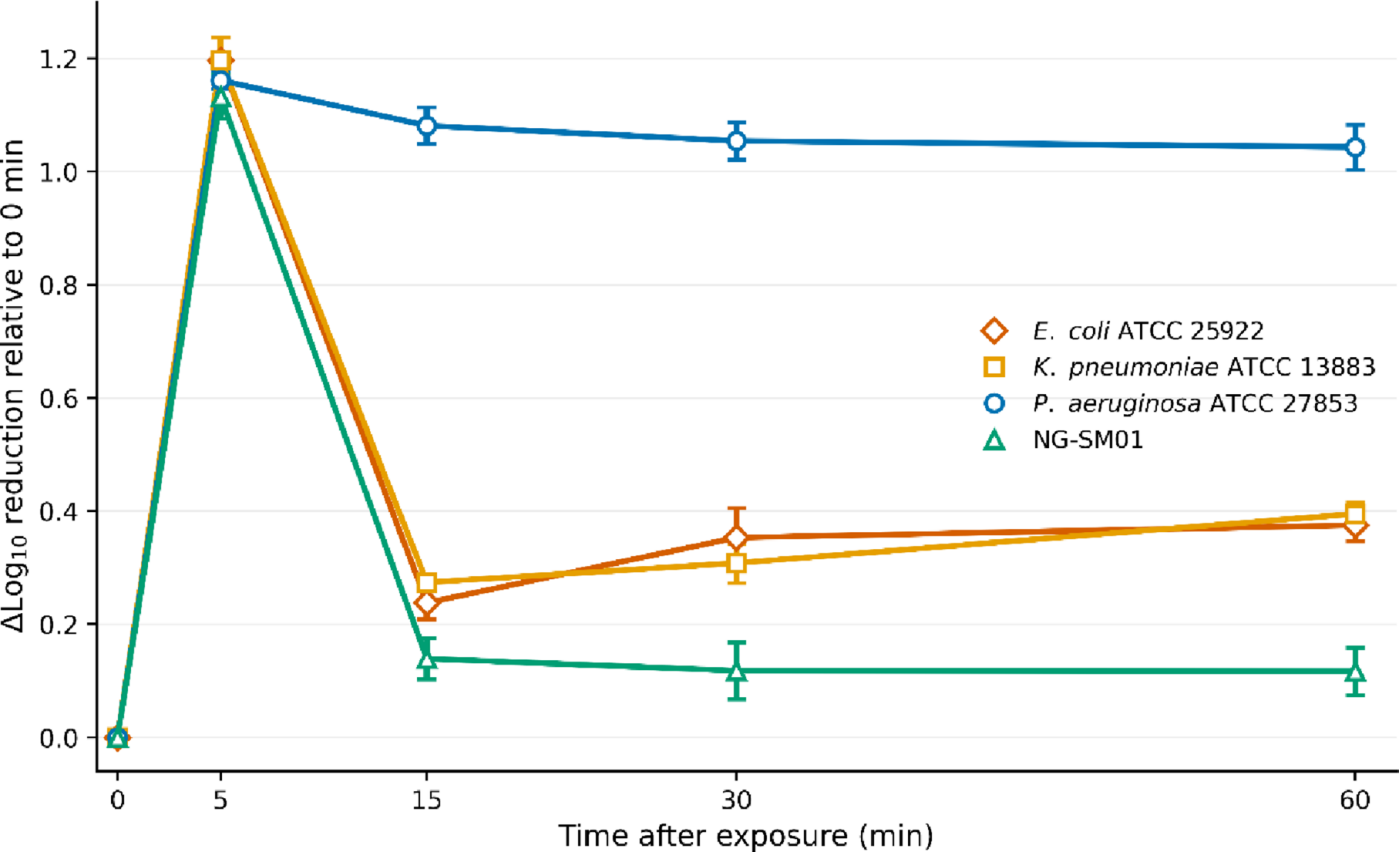
Time-kill kinetics under 2.0 mM H₂O₂ oxidative-stress challenge, expressed as Δlog10 reduction relative to baseline. Mid-log cultures were exposed to 2.0 mM H₂O₂ and sampled at 0, 5, 15, 30, and 60 min, immediately quenched with catalase, and enumerated by CFU plating. The 0-min value represents the paired baseline sampled immediately before H₂O₂ exposure. Δlog_10_ was calculated as log_10_(CFU/mL at baseline before H₂O₂ addition) − log_10_(CFU/mL at time t). Points show mean ± SD from three independent biological replicates (*n* = 3). H₂O₂ was used as a laboratory oxidative-stress proxy and should not be interpreted as direct evidence of free-chlorine or chloramine tolerance.

To complement the dose–response assay, we quantified short-term survival kinetics under acute 2.0 mM H₂O₂ challenge by tracking viable counts over time and expressing outcomes as log_10_ reduction relative to baseline (Δlog_10_). This assay was used to compare strain-associated response patterns under H₂O₂-mediated oxidative stress and was not interpreted as direct evidence of free-chlorine or chloramine tolerance (Fig. 11; Supplementary Fig. S17). Summary kinetics are provided in Supplementary Table S60. All strains showed an early reduction at 5 min, with mean Δlog10 values ranging from 1.132 to 1.197, but trajectories diverged thereafter. *P. aeruginosa* ATCC 27,853 maintained the largest and most sustained reduction, with Δlog_10_ = 1.161 ± 0.015 at 5 min and 1.043 ± 0.040 at 60 min. *K. pneumoniae* ATCC 13,883 and *E. coli* ATCC 25,922 showed partial recovery after the initial 5-min reduction and stabilized at modest reductions by 60 min, with Δlog_10_ values of 0.395 ± 0.021 and 0.375 ± 0.028, respectively. In contrast, NG-SM01 showed a low-reduction plateau after the initial 5-min drop, remaining close to baseline from 15 to 60 min: 0.139 ± 0.036 at 15 min, 0.118 ± 0.050 at 30 min, and 0.117 ± 0.042 at 60 min (Fig. 11Supplementary Table S60). Two-way ANOVA confirmed significant effects of strain and time and a significant strain × time interaction: strain, F(3,32) = 901.07, *P* = 5.66 × 10⁻³¹, partial η² = 0.988; time, *F*(3,32) = 1205.37, *P* = 5.65 × 10⁻³³, partial η² = 0.991; interaction, *F*(9,32) = 104.51, *P* = 3.76 × 10⁻²¹, partial η² = 0.967 (Supplementary Table S61). Tukey HSD showed no significant differences between NG-SM01 and the reference strains at 5 min, with all adjusted P values ≥ 0.181. However, NG-SM01 differed from all reference strains at 15–60 min, most prominently from *P. aeruginosa*, with mean differences ranging from − 0.942 to − 0.926 and all adjusted *P* values = 2.052 × 10⁻¹⁴ (Supplementary Table S62).

## Discussion

In disinfectant-managed drinking-water distribution systems (DWDS), biofilms represent an important ecological interface where microorganisms may experience residual disinfectant exposure, nutrient limitation, surface attachment, and intermittent release into bulk water^1–6,14^. Within this context, NG-SM01 is best interpreted as a genome-resolved single-isolate case study that supports a cautious, hypothesis-generating “Switch–Shield” framework. In this proposed framework, the “switch” component refers to candidate stress-response and mobilome-associated regulatory features, whereas the “shield” component refers to candidate EPS/envelope-remodeling features that may be relevant to surface-associated survival. Lineage context provides the foundation for this interpretation. Phylogenomics placed NG-SM01 within the Sgn4 genomospecies of the *Stenotrophomonas maltophilia* complex, while MLST assigned the isolate to ST1409 with the newly curated *guaA* allele 909, providing a standardized tracking handle for future environmental and clinical comparisons^25,46^. The proposed “shield” component is supported by GI_6, which contains an EPS/envelope-associated locus including *algL*, a WecB/TagA/CpsF-family glycosyltransferase with a GT26 domain (NG_SM01_02055), and capsule/EPS-associated backbone genes. Remote BLASTp recovered a small number of near-identical NG_SM01_02055 homologs within the *S. maltophilia* complex, indicating that the relevant feature is not global sequence rarity but the local island-associated cassette architecture. Within the selected comparison panel, this locus was panel-restricted and co-localized with EPS/envelope-associated genes, suggesting a candidate module related to surface-matrix or envelope remodeling. The phenotype data are consistent with this cautious interpretation. NG-SM01 produced reproducible moderate crystal-violet biofilm biomass, with OD595corr of 0.1094 ± 0.0382 and a growth-normalized index of 0.1078 ± 0.0393. This supports biofilm competence under static microtiter conditions but does not indicate an exceptionally strong biofilm phenotype relative to all comparator strains. Biofilm formation is relevant to DWDS ecology because biofilms can reduce effective biocide exposure and contribute to persistence or release of cells into bulk water^1–3,47,48^. Recent comparative genomic work showing lineage-associated diversity regions in clinical *S. maltophilia* further supports the broader concept that persistence-associated traits may vary across the complex^49^. The proposed “switch” component is supported primarily by GI_4, which was annotated by ICEberg 3.0 as containing an IME-like region spanning edge_1:533,895–553,050. This region is bounded by attL/attR repeats, lies adjacent to tRNA-Ser, and encodes a MOBQ-family relaxase and phage-type integrases^30^. These features support an IME-like architectural assignment. GI_4 also lies near recX–recA–lexA and contains yejK and csrA, placing the region in proximity to SOS-associatedregulatory and repair genes. This genomic organization may provide a candidate link between local genomeplasticity and stress-response circuitry,consistent with evidence that SOS-response activation cancontribute to bacterial tolerance and persister-cell formation^50^. The H₂O₂ assays provide additional phenotypic context for the proposed “switch” component. NG-SM01 showed a plateau-like response during acute H₂O₂ exposure and a low-reduction response pattern during short-term time-kill analysis after the initial 5-min drop. These observations support a distinct response pattern under laboratory H₂O₂-mediated oxidative stress. Because H₂O₂ was used as an oxidative-stress proxy, these results are most appropriately interpreted as evidence of laboratory oxidative-stress response behavior rather than direct evidence of free-chlorine or chloramine tolerance in DWDS. Other genome-screening layers are consistent with this candidate persistence-related framework. NG-SM01 carried an intrinsic defense background typical of the *S. maltophilia* complex, including efflux-associated systems, chromosomal aminoglycoside-modifying enzymes, and intrinsic β-lactamase alleles, which are relevant to the clinical importance of this complex^9–11,51,52^. VFDB screening identified only a limited canonical virulence signal, dominated by a type IV pili/twitching motility-associated PilG match, supporting attachment-related potential rather than expanded virulence. BacMet screening identified candidate metal/biocide tolerance-associated loci among the panel-restricted annotated genes, including CzcR-like, AdeL-like, and YfeB-like components^53^. These signals align with cross-stress tolerance hypotheses, particularly in treated-water environments where metal, nutrient, and oxidative stresses may overlap. Similarly, gapseq reconstruction predicted broad transporter capacity, including 1,920 transporter entries, with 23% categorized as metal-related^13^. NG-SM01 also encoded predicted nutrient-acquisition systems relevant to oligotrophic environments, including a complete phosphate uptake module (*pstS/pstC/pstA/pstB* with *phoU*), iron-uptake-associated genes such as *fiu* and *feoB*, and TonB-associated components. These features are compatible with resource acquisition in low-nutrient engineered-water habitats. However, they remain computational predictions and require experimental validation under defined nutrient-limiting conditions. The broader implication is that DWDS should be viewed as exposure-relevant ecosystems in which biofilms, residual disinfectants, and oligotrophic conditions may shape opportunistic environmental lineages. Treatment-driven shifts can reshape drinking-water microbiomes^7^, chlorination has been associated with resistance-gene dynamics^8^, and opportunistic pathogens may persist in biofilm reservoirs that intermittently seed bulk water^14^. This is clinically relevant because *S. maltophilia* infections can be severe in vulnerable populations, including immunocompromised patients, neonates, the elderly, and individuals with chronic lung disease or indwelling devices^11,54^. Overall, NG-SM01 supports a cautious Switch–Shield working model in which GI_4-associated stress-response/mobilome features and GI_6-associated EPS/envelope-remodeling features may be relevant to persistence-related traits. The strength of this model lies in the convergence of genome architecture, comparative genomics, functional prediction, and laboratory phenotypes, while its interpretation should remain focused on candidate mechanisms rather than proven causality.

## Conclusion

NG-SM01 is a drinking-water distribution system isolate assigned to the *Stenotrophomonas maltophilia* complex, genomospecies Sgn4. The closed genome assembly, together with phylogenomics and MLST, placed NG-SM01 within Sgn4 and defined ST1409 through the newly curated PubMLST allele *guaA*-909, providing a standardized basis for future lineage tracking. Genome analysis identified two host-discordant candidate regions that support a hypothesis-generating Switch–Shield working model: GI_4, an IME-like region located near SOS-associated regulatory and repair genes, and GI_6, an EPS/envelope-associated locus carrying *algL* and a GT26-family glycosyltransferase within a panel-restricted cassette architecture. Additional predictive layers, including BacMet Tier-1 metal/biocide tolerance-associated signals and gapseq-inferred transporter breadth, together with reproducible moderate biofilm formation and plateau-like responses under acute H₂O₂ oxidative stress, are consistent with candidate features potentially relevant to persistence. However, these findings should be interpreted as convergent genomic and phenotypic evidence supporting a testable model rather than direct proof of causal persistence mechanisms. The limited canonical virulence signal, together with an intrinsic resistance background typical of the *S. maltophilia* complex, highlights the relevance of environmental lineage surveillance in drinking-water systems, particularly for exposure assessment in vulnerable populations.

## Limitations and future perspectives

This study has several limitations that are important for interpretation. First, it is based on a single drinking-water isolate; therefore, the findings cannot establish population-level patterns across the *S. maltophilia* complex or across DWDS environments. Second, the comparative analysis is constrained by the size, taxonomic composition, and quality of the available reference panel. Accordingly, genes described as panel-restricted are defined only relative to the genomes analyzed and may be detected more broadly as additional *S. maltophilia* complex genomes become available. Third, H₂O₂ was used as a controlled oxidative-stress proxy and does not fully recapitulate the chemistry, exposure dynamics, or biofilm microenvironments associated with free chlorine or chloramine in DWDS. Finally, the study did not include transcriptional profiling, mutant analysis, GI excision/mobility assays, or DWDS-mimetic flow/pipe-reactor validation; therefore, direct causality between GI_4/GI_6 and the observed phenotypes remains unproven. Future work should test the proposed Switch–Shield framework under DWDS-mimetic conditions, including controlled free-chlorine and chloramine gradients, flow/pipe-reactor biofilm models, and quantification of detachment or shedding into bulk water. Targeted RT-qPCR and/or RNA-seq could assess stress-responsive expression of priority GI_4/GI_6 genes, including IME/SOS-associated regulators and EPS/envelope-remodeling genes. Genetic perturbation or comparative analysis of naturally matched strains differing in GI_4/GI_6 content would further help test whether these loci are causally linked to oxidative-stress response patterns, biofilm persistence, or surface-associated fitness. These studies will be required to determine whether the proposed Switch–Shield model represents a causal persistence mechanism or a useful framework for prioritizing candidate loci in environmental *Stenotrophomonas* surveillance.

## Supplementary Information

Below is the link to the electronic supplementary material.

Supplementary Material 1.

## Data Availability

The raw sequencing data for Stenotrophomonas sp. NG-SM01 (Stenotrophomonas maltophilia complex; Sgn4) were deposited in the NCBI Sequence Read Archive under BioProject PRJNA1419804 and BioSample SAMN55197924. The Illumina and Oxford Nanopore sequencing runs are available under accession numbers SRR37144688 and SRR37144689, respectively. The genome assembly is publicly available in GenBank under WGS project JBWJAL01 and accession JBWJAL010000000, version JBWJAL010000001.1. The complete chromosome record is available as CM149968.1. The newly curated PubMLST guaA allele 909 and the corresponding allelic profile ST1409 were accepted in the PubMLST Stenotrophomonas maltophilia database; NG-SM01 is registered under PubMLST isolate ID 2048.

## Abbreviations

AMR: Antimicrobial resistance
ARG: Antibiotic resistance gene
AST: Antimicrobial susceptibility testing
CFU: Colony-forming unit
DWDS: Drinking-water distribution system
EPS: Extracellular polymeric substances
GI: Genomic Island
HGT: Horizontal gene transfer
ICE: Integrative and conjugative element
IS: Insertion sequence
LNF: Lactose non-fermenter
MBL: Metallo-β-lactamase
MLST: Multilocus sequence typing
RND: Resistance–nodulation–division (efflux system family)
ROS: Reactive oxygen species
SNP: Single-nucleotide polymorphism
SOS: SOS DNA-damage response
ST: Sequence type
T4SS: Type IV secretion system
TSI: Triple sugar iron agar
VFDB: Virulence Factors Database
WGS: Whole-genome sequencing
