# Supplementary material for: Genomic analysis identifies candidate SOS-associated and EPS/envelope-remodeling islands in a drinking-water Stenotrophomonas maltophilia complex isolate
Source: Sci Rep. 2026 Jun 28;16:19640. doi: 10.1038/s41598-026-58581-0 (PMC13310844; doi:10.1038/s41598-026-58581-0)
Supplement: Supplementary file 1 — Supplementary Material 1. [file 41598_2026_58581_MOESM1_ESM.docx]

**Supplementary Table S1. Software tools and databases used in this study (versions, links, access date)**

| **Category** | **Tool / Database** | **Version / release used** |
| --- | --- | --- |
| OS / runtime | Ubuntu Linux | 22.04.5 LTS |
| OS / runtime | Python | 3.10.0 |
| OS / runtime | Python | 3.8.20 |
| Read QC (ONT) | Filtlong | 0.2.1 |
| Read QC (Illumina) | fastp | 0.20.0 |
| Read QC summary | NanoPlot | 1.40.2 |
| Read QC summary | MultiQC | 1.9.0 |
| Read subsampling | Seqtk | 1 |
| Assembly | Flye | 2.9.3 |
| ONT polishing | Medaka | 1.8 |
| Read mapping | BWA | 0.7.17 |
| Short-read polishing | Polypolish | 0.6.0 |
| Assembly QC | QUAST | 5.2.0 |
| Genome quality | CheckM2 | 1.0.1 |
| Distance / placement | Mash | 1.1 |
| Annotation | Bakta | 1.8.2 |
| Annotation | Prokka | 1.14.6 |
| Orthology annotation | eggNOG-mapper | 2.1.8 |
| eggNOG database | eggNOG DB | 5.0.2 |
| Domain annotation | InterProScan | 5.59-91.0 |
| MLST platform | PubMLST (BIGSdb) | Web |
| Pangenome | Panaroo | 1.5.2 |
| Pan-GWAS | Scoary | 1.6.16 |
| Synteny visualization | clinker / clustermap.js | 0.0.32 |
| CAZyme annotation | dbCAN3 | 3.0.6 |
| CAZyme evidence | HMMER | 3.4 |
| Alignments / screening | DIAMOND | 2.1.19 |
| Genomic islands | IslandViewer4 | Web |
| ICE / mobile elements | ICEberg | 3 |
| Integrons | IntegronFinder | Galaxy 2.0.5 |
| Prophage prediction | PHASTER | Web |
| Prophage annotation | Pharokka | Galaxy 1.3.2 |
| CRISPR | MinCED | Galaxy 0.1.5 |
| CRISPR | CRT | Galaxy 1.2.0 |
| CRISPR confirmation | CRISPRCasFinder | Web |
| AMR (primary) | AMRFinderPlus | 4.0.23 |
| AMR/Virulence wrapper | ABRicate | 1.0.1 |
| Virulence DB | VFDB | (update: 25-01-2026) |
| AMR DB | CARD | 4.0.1 |
| AMR DB | ResFinder | 4.7.2 |
| Metal/biocide DB | BacMet2 EXP | FASTA date: 2017-12-06 |
| Metabolic reconstruction | gapseq | 1.4.0 |
| Visualization | CGView | 2.0.3 |
| Visualization | Proksee | Web |
| Synteny analysis | Clinker | 0.0.32 |
| Data handling | pandas | 2.0.3 |
| Plotting | matplotlib | 3.7.3 |

**Supplementary Table S2. Legend for eggNOG/COG single-letter functional categories used in this study**

| **COG letter** | **Functional category** |
| --- | --- |
| C | Energy production & conversion |
| D | Cell cycle control, cell division, chromosome partitioning |
| E | Amino acid transport & metabolism |
| F | Nucleotide transport & metabolism |
| G | Carbohydrate transport & metabolism |
| H | Coenzyme transport & metabolism |
| I | Lipid transport & metabolism |
| J | Translation, ribosomal structure & biogenesis |
| K | Transcription |
| L | Replication, recombination & repair |
| M | Cell wall/membrane/envelope biogenesis |
| N | Cell motility |
| O | Posttranslational modification, protein turnover, chaperones |
| P | Inorganic ion transport & metabolism |
| Q | Secondary metabolites biosynthesis, transport & catabolism |
| R | General function prediction only |
| S | Function unknown |
| T | Signal transduction mechanisms |
| U | Intracellular trafficking, secretion & vesicular transport |
| V | Defense mechanisms |
| W | Extracellular structures |
| Y | Nuclear structure (rare in bacteria) |
| Z | Cytoskeleton (rare in bacteria) |

**Supplementary Table S3. Legend for CAZyme classes codes used in this study**

| **Abbreviation** | **Full name** | **Biological function** |
| --- | --- | --- |
| GH | Glycoside hydrolases | Enzymes that hydrolyze glycosidic bonds |
| GT | Glycosyltransferases | Enzymes that build glycosidic bonds by transferring sugars |
| CE | Carbohydrate esterases | Remove acetyl/succinyl (and related) esters from carbohydrates |
| PL | Polysaccharide lyases | Cleavage by β-elimination (not hydrolysis) |
| AA | Auxiliary activities | Redox enzymes that assist carbohydrate conversion (often oxidative) |
| CBM | Carbohydrate-binding modules | Non-catalytic binding domains that tether enzymes to substrates |

**Supplementary Table S4:** **Biochemical profile and colony phenotype of NG-SM01.**

| **Biochemical Test** | **Result** |
| --- | --- |
| Catalase | Positive |
| Coagulase | Negative |
| Oxidase | Positive |
| Urease | Negative |
| Indole | Negative |
| Bile Esculin | Negative |
| Mannitol Salt Agar | No Growth |
| Eosin Methylene Blue (EMB) | Growth (Lactose Non-Fermenter, LNF) |
| MacConkey Agar | Growth (Lactose Non-Fermenter, LNF) |
| Sorbitol MacConkey Agar | Growth (Colorless) |
| Motility | Positive |
| Mannitol Fermentation | Negative |
| Sorbitol Fermentation | Negative |
| Triple Sugar Iron (TSI) Agar | Alkaline slant / Alkaline butt (K/K) |
| Citrate Utilization | Positive |
| β-galactosidase (ONPG) | Positive |
| Muller Hinton Agar | Yellow (Gold) Pigment |
| Trypticase Soy Agar (TSA) | Yellow (Gold) Pigment |
| Nitrate Reduction | Positive |
| Blood Agar | Growth |
| Hemolysis | None (Gamma, N) |
| Oxidation of Glucose | Positive (P) |
| Fermentation of Glucose | Negative (N) |

**Supplementary Table S5:** **Disk diffusion zones for NG-SM01 and interpretation (CLSI where available).**

| **Antibiotic class** | **Antibiotic** | **Disk code** | **Disk (µg)** | **Zone (mm)** | **CLSI interpretation*** |
| --- | --- | --- | --- | --- | --- |
| β-lactams (penicillins) | Ampicillin | AMP | 10 | 0 | NA |
|  | Amoxicillin–clavulanate | AMC | 20/10 (30) | 0 | NA |
| β-lactams (penicillin + inhibitor) | Ampicillin–sulbactam | SAM | 10/10 (20) | 0 | NA |
|  | Piperacillin–tazobactam | TZP | 100/10 (110) | 11.4 | NA |
| β-lactams (cephalosporins) | Cefoxitin | FOX | 30 | 0 | NA |
|  | Cefotaxime | CTX | 30 | 0 | NA |
|  | Cefepime | FEP | 30 | 0 | NA |
|  | Ceftriaxone | CRO | 30 | 0 | NA |
|  | Ceftiofur | FUR | 30 | 0 | NA |
| β-lactams (monobactam) | Aztreonam | ATM | 30 | 0 | NA |
| β-lactams (carbapenems) | Imipenem | IPM | 10 | 0 | NA |
|  | Meropenem | MEM | 10 | 0 | NA |
| Aminoglycosides | Amikacin | AMK | 30 | 17.7 | NA |
|  | Gentamicin | CN | 10 | 21.2 | NA |
| Tetracyclines | Tetracycline | TE | 30 | 12.7 | NA |
| Fluoroquinolones | Enrofloxacin | ENR | 5 | 30 | NA |
|  | Ciprofloxacin | CIP | 5 | 23 | NA |
|  | Levofloxacin | LEV | 5 | 22 | S |
| Folate pathway inhibitors | Sulfamethoxazole–trimethoprim | SXT | 1.25/23.75 (25) | 21 | S |
| Other | Fosfomycin | FOS | 50 | 0 | NA |
|  | Nitrofurantoin | NIT | 300 | 0 | NA |

* CLSI interpretation applied only where CLSI disk diffusion criteria exist for Stenotrophomonas maltophilia. NA = Not applicable (no CLSI disk diffusion breakpoint/interpretive criteria for *S. maltophilia*; zone diameter reported only).

**Supplementary Table S6. Reference genome panel used for phylogenomics.**
Assembly accessions, primary sequence identifiers, strain/isolate labels, and core assembly features (genome length, GC%, and N50) for NG-SM01 and the curated comparative panel, including outgroups and the NBRC 14161 type strain.

| **Assembly accession** | **PrimarySeqID** | **Organism (strain/isolate)** | **Total length (bp)** | **GC (%)** | **N50 (bp)** |
| --- | --- | --- | --- | --- | --- |
| GCF_025642255.1 | NZ_CP106759.1 | S. maltophilia_P | 4120518 | 66.94 | 4120518 |
| GCF_002138415.1 | NZ_CP015612.1 | S. maltophilia_G | 4668743 | 66.25 | 4668743 |
| GCF_001274595.1 | NZ_CP011010.1 | S. maltophilia_AJ | 4804002 | 66.65 | 4804002 |
| GCF_004346925.1 | NZ_CP031167.1 | S. maltophilia_A | 4564481 | 66.38 | 4564481 |
| GCF_003205835.1 | NZ_CP029773.1 | S. maltophilia_AL | 4891004 | 66.27 | 4891004 |
| GCF_001431665.1 | NZ_LLXV01000001.1 | S. maltophilia_K | 4484616 | 66.84 | 83392 |
| GCF_006970445.1 | NZ_CP033877.1 | S. maltophilia_AU | 4549249 | 66.72 | 4549249 |
| GCF_900186865.1 | NZ_LT906480.1 | S. maltophilia | 5004261 | 66.12 | 5004261 |
| GCF_006974125.1 | NZ_CP037858.1 | S. maltophilia_O | 4554224 | 66.52 | 4554224 |
| GCF_002799165.1 | NZ_NERE01000004.1 | S. maltophilia_Q | 4846973 | 66.11 | 2480350 |
| GCF_002799155.1 | NZ_NERD01000002.1 | S. maltophilia_R | 4422561 | 66.81 | 4313447 |
| GCF_002799245.1 | NZ_NEQV01000001.1 | S. maltophilia_S | 4746430 | 66.55 | 1121887 |
| GCF_000020665.1 | NC_011071.1 | S. maltophilia_L | 4573969 | 66.3 | 4573969 |
| GCF_013464915.1 | NZ_RAVT01000001.1 | S. maltophilia_AT | 4646463 | 66.66 | 74914 |
| GCF_013004645.1 | NZ_JABEME010000001.1 | S. maltophilia_AM | 4573818 | 66.33 | 310943 |
| GCF_001676385.1 | NZ_LYVJ01000001.1 | S. maltophilia_J | 4123397 | 66.69 | 276364 |
| GCF_004920835.1 | NZ_QFHO01000010.1 | S. maltophilia_AQ | 4559750 | 66.14 | 122165 |
| GCF_004684085.1 | NZ_SRHZ01000001.1 | S. maltophilia_AS | 4626840 | 66.04 | 90353 |
| NBRC_14161 | BCUI01000001.1 | S. maltophilia NBRC 14161 | 4936723 | 66.19 | 65715 |

Note: Reference labels follow internal panel naming based on source database annotations and are not intended to revise the genome-based placement of NG-SM01 within the *S. maltophilia* complex/Sgn4 or to imply assignment of NG-SM01 to *S. maltophilia sensu stricto*.

**Supplementary Table S7. BUSCO completeness metrics for phylogenomic panel genomes.**
BUSCO summary statistics (Complete, Duplicated, Fragmented, Missing) for NG-SM01 and all reference genomes used in phylogenomic reconstruction.

| **Assembly accession** | **BUSCO C (%)** | **BUSCO D (%)** | **BUSCO F (%)** | **BUSCO M (%)** |
| --- | --- | --- | --- | --- |
| GCF_000020665.1 | 99.2 | 0 | 0.8 | 0 |
| GCF_001274595.1 | 99.2 | 0 | 0.8 | 0 |
| GCF_001431665.1 | 97.6 | 0 | 2.4 | 0 |
| GCF_001676385.1 | 97.6 | 0.8 | 2.4 | 0 |
| GCF_002138415.1 | 99.2 | 0 | 0.8 | 0 |
| GCF_002799155.1 | 99.2 | 0 | 0.8 | 0 |
| GCF_002799165.1 | 100 | 0 | 0 | 0 |
| GCF_002799245.1 | 100 | 0 | 0 | 0 |
| GCF_003205835.1 | 99.2 | 0 | 0.8 | 0 |
| GCF_004346925.1 | 100 | 0 | 0 | 0 |
| GCF_004684085.1 | 99.2 | 0 | 0.8 | 0 |
| GCF_004920835.1 | 100 | 0 | 0 | 0 |
| GCF_006970445.1 | 99.2 | 0 | 0.8 | 0 |
| GCF_006974125.1 | 99.2 | 0 | 0.8 | 0 |
| GCF_013004645.1 | 100 | 0 | 0 | 0 |
| GCF_013464915.1 | 100 | 0 | 0 | 0 |
| GCF_025642255.1 | 100 | 0 | 0 | 0 |
| GCF_900186865.1 | 100 | 0 | 0 | 0 |
| NBRC_14161 | 100 | 0 | 0 | 0 |

**Supplementary Table S8. FastANI comparisons supporting genome-based placement of NG-SM01.**Pairwise ANI and alignment fraction (AF) values between NG-SM01 and selected reference genomes, including the closest GTDB reference and the NBRC 14161 type strain.

| **Query genome** | **Reference genome (accession)** | **Reference species/strain** | **FastANI (%)** | **AF (Aligned/Total)** | **AF (%)** | **Aligned fragments** | **Total fragments** | **Category** |
| --- | --- | --- | --- | --- | --- | --- | --- | --- |
| NG-SM01 | GCF_025642255.1 | S. maltophilia_P | 98.8581 | 1309/1370 | 95.55 | 1309 | 1370 | Environmental  (rhizosphere) |
| NG-SM01 | GCF_003205835.1 | S. maltophilia_AL | 88.6007 | 1160/1370 | 84.67 | 1160 | 1370 | Environmental  (wastewater) |
| NG-SM01 | GCF_002799245.1 | S. maltophilia_S | 88.5467 | 1116/1370 | 81.46 | 1116 | 1370 | Environmental  (brackish water) |
| NG-SM01 | GCF_006970445.1 | S. maltophilia_AU | 88.4857 | 1122/1370 | 81.9 | 1122 | 1370 | Environmental  (rhizosphere) |
| NG-SM01 | GCF_001274595.1 | S. maltophilia_AJ | 88.4684 | 1163/1370 | 84.89 | 1163 | 1370 | Clinical  (blood) |
| NG-SM01 | GCF_900101175.1 | S. pavanii | 88.4556 | 1159/1370 | 84.6 | 1159 | 1370 | Environmental  (plant-associated / rhizosphere lineage) |
| NG-SM01 | GCF_002799155.1 | S. maltophilia_R | 88.4273 | 1148/1370 | 83.8 | 1148 | 1370 | Clinical  (urine) |
| NG-SM01 | GCF_004346925.1 | S. maltophilia_A | 88.3782 | 1148/1370 | 83.8 | 1148 | 1370 | Environmental  (soil) |
| NG-SM01 | GCF_001431665.1 | S. maltophilia_K | 88.3151 | 1126/1370 | 82.19 | 1126 | 1370 | Environmental  (Piper betle) |
| NG-SM01 | GCF_002799165.1 | S. maltophilia_Q | 88.3095 | 1154/1370 | 84.23 | 1154 | 1370 | Clinical  (blood) |
| NG-SM01 | GCF_900186865.1 | S. maltophilia | 88.3072 | 1157/1370 | 84.45 | 1157 | 1370 | Clinical  (mouth) |
| NG-SM01 | GCF_006974125.1 | S. maltophilia_O | 88.3003 | 1131/1370 | 82.55 | 1131 | 1370 | Environmental  (aerobic granular sludge) |
| NG-SM01 | GCF_013464915.1 | S. maltophilia_AT | 88.2865 | 1120/1370 | 81.75 | 1120 | 1370 | Clinical  (perineum swab) |
| NG-SM01 | GCF_002138415.1 | S. maltophilia_G | 88.2797 | 1158/1370 | 84.53 | 1158 | 1370 | Environmental  (soil) |
| NG-SM01 | GCF_013004645.1 | S. maltophilia_AM | 88.2635 | 1126/1370 | 82.19 | 1126 | 1370 | Environmental |
| NG-SM01 | NBRC_14161 | S. maltophilia NBRC 14161 | 88.2545 | 1086/1370 | 79.27 | 1086 | 1370 | Clinical  (human oropharyngeal region) |
| NG-SM01 | GCF_000020665.1 | S. maltophilia_L | 88.19 | 1159/1370 | 84.6 | 1159 | 1370 | Unknown  (not reported) |
| NG-SM01 | GCF_000382065.1 | S. hibiscicola | 88.1526 | 1149/1370 | 83.87 | 1149 | 1370 | Environmental  (plant-associated) |
| NG-SM01 | GCF_004920835.1 | S. maltophilia_AQ | 88.0995 | 1112/1370 | 81.17 | 1112 | 1370 | Environmental  (hospital hot water tap / built environment) |
| NG-SM01 | GCF_004684085.1 | S. maltophilia_AS | 87.9288 | 1092/1370 | 79.71 | 1092 | 1370 | Environmental  (gold particle resident biofilm) |
| NG-SM01 | GCF_001676385.1 | S. maltophilia_J | 87.8128 | 1099/1370 | 80.22 | 1099 | 1370 | Environmental  (horse manure) |

Note: Reference labels follow internal panel naming based on source database annotations and are not intended to revise the genome-based placement of NG-SM01 within the *S. maltophilia* complex/Sgn4 or to imply assignment of NG-SM01 to *S. maltophilia sensu stricto*.

**Supplementary Table S9. 16S rRNA BLASTn top matches for NG-SM01.**Top database hits for the NG-SM01 16S rRNA sequence with query coverage, percent identity, and accession identifiers.

| **Description** | **Scientific Name** | **Max Score** | **Total Score** | **Query Cover** | **E value** | **Per. ident** | **Acc. Len** | **Accession** |
| --- | --- | --- | --- | --- | --- | --- | --- | --- |
| Stenotrophomonas maltophilia strain IAM 12423 16S ribosomal RNA, partial sequence | Stenotrophomonas maltophilia | 2765 | 2765 | 100% | 0 | 99.15 | 1538 | NR_041577.1 |
| Stenotrophomonas maltophilia strain LMG 958 16S ribosomal RNA, partial sequence | Stenotrophomonas maltophilia | 2700 | 2700 | 97% | 0 | 99.13 | 1500 | NR_119220.1 |
| Stenotrophomonas maltophilia strain NBRC 14161 16S ribosomal RNA, partial sequence | Stenotrophomonas maltophilia | 2645 | 2645 | 95% | 0 | 99.12 | 1470 | NR_113648.1 |
| Stenotrophomonas maltophilia strain ATCC 13637 16S ribosomal RNA, partial sequence | Stenotrophomonas maltophilia | 2638 | 2638 | 95% | 0 | 99.11 | 1467 | NR_112030.1 |

**Supplementary Table S10. Node-by-node support summary for the core-genome phylogeny.**Key splits defining NG-SM01 placement and deeper clade structure, with corresponding branch lengths and bootstrap support values.

| **Node ID** | **Relationship** | **Descendant taxa (split)** | **Branch length (subs/site)** | **Bootstrap (%)** |
| --- | --- | --- | --- | --- |
| **N1** | **NG-SM01 is sister to maltophilia_P (GCF_025642255.1)** | **NG-SM01 + maltophilia_P** | **0.1657** | **100** |
| N2 | Clade expands by adding maltophilia_J | NG-SM01 + maltophilia_P + maltophilia_J | 0.011 | 100 |
| N3 | Clade expands by adding maltophilia_K | NG-SM01 + maltophilia_P + maltophilia_J + maltophilia_K | 0.0054 | 100 |
| N4 | NG-SM01 placement clade within broader group | Includes NBRC_14161, hibiscicola (GCF_000382065.1), and multiple maltophilia genomes | 0.0099 | 100 |
| N5 | Same placement clade | Same as N4 | 0.0045 | 99 |

Note: Reference labels follow internal panel naming based on source database annotations and are not intended to revise the genome-based placement of NG-SM01 within the *S. maltophilia* complex/Sgn4 or to imply assignment of NG-SM01 to *S. maltophilia sensu stricto*.

**Supplementary Table S11. Nucleotide substitution map for NG-SM01 *guaA* relative to PubMLST *guaA*_544.** The table reports the ungapped reference position (1-based), reference base in PubMLST *guaA*(544), query base in NG-SM01, and alignment column for each of the five SNPs identified across the full 552-bp *guaA* locus. Following PubMLST curator review, the NG-SM01 sequence was accepted as *guaA* allele 909, and the complete allelic profile was assigned ST1409.

| **Ref_allele** | **Query_allele** | **Pos_1based** | **Ref_base** | **Query_base** | **Alignment_col** |
| --- | --- | --- | --- | --- | --- |
| guaA_544 | NG-SM01_guaA-909 | 276 | **A** | **G** | 276 |
| guaA_544 | NG-SM01_guaA-909 | 279 | **C** | **G** | 279 |
| guaA_544 | NG-SM01_guaA-909 | 303 | **T** | **C** | 303 |
| guaA_544 | NG-SM01_guaA-909 | 390 | **G** | **A** | 390 |
| guaA_544 | NG-SM01_guaA-909 | 441 | **G** | **A** | 441 |

**Supplementary Table S12. Coordinates and size of NG-SM01 panel-restricted blocks.**Genomic block identifiers with start/end coordinates, number of panel-restricted per block, and block span (bp) on the NG-SM01 chromosome.

| **block_id** | **start** | **end** | **n_panel-restricted genes** | **span_bp** |
| --- | --- | --- | --- | --- |
| **block_1** | 286650 | 291377 | 4 | 4728 |
| **block_2** | 333971 | 351622 | 7 | 17652 |
| **block_3** | 435823 | 442305 | 4 | 6483 |
| **block_4** | 534676 | 550604 | 16 | 15929 |
| **block_5** | 649565 | 649777 | 1 | 213 |
| **block_6** | 1100244 | 1101367 | 2 | 1124 |
| **block_7** | 1384036 | 1386808 | 2 | 2773 |
| **block_8** | 1512940 | 1513647 | 1 | 708 |
| **block_9** | 1590070 | 1596173 | 3 | 6104 |
| **block_10** | 1724719 | 1728434 | 4 | 3716 |
| **block_11** | 1957395 | 1958153 | 1 | 759 |
| **block_12** | 1985404 | 1986251 | 2 | 848 |
| **block_13** | 2107942 | 2108196 | 1 | 255 |
| **block_14** | 2249097 | 2256979 | 7 | 7883 |
| **block_15** | 2393359 | 2394159 | 1 | 801 |
| **block_16** | 2493490 | 2511345 | 17 | 17856 |
| **block_17** | 2658613 | 2663856 | 3 | 5244 |
| **block_18** | 2736258 | 2736461 | 1 | 204 |
| **block_19** | 2913863 | 2914336 | 1 | 474 |
| **block_20** | 2963374 | 2963499 | 1 | 126 |
| **block_21** | 2986066 | 2986182 | 1 | 117 |
| **block_22** | 3289476 | 3289802 | 1 | 327 |
| **block_23** | 3332142 | 3378973 | 54 | 46832 |
| **block_24** | 3633338 | 3639188 | 5 | 5851 |
| **block_25** | 3759671 | 3760720 | 1 | 1050 |
| **block_26** | 4034274 | 4041425 | 7 | 7152 |
| **block_27** | 4067605 | 4095571 | 16 | 27967 |

**Supplementary Table S13. Gene-level annotation for panel-restricted geneswithin NG-SM01 blocks.** Per-gene coordinates, strand, locus tags, gene/product annotations, and block membership for NG-SM01 panel-restricted genes.

| **contig** | **start** | **end** | **strand** | **locus_tag** | **gene** | **product** | **block_id** |
| --- | --- | --- | --- | --- | --- | --- | --- |
| edge_1 | 286650 | 286784 | - | NG_SM01_00239 | | hypothetical protein | block_1 |
| edge_1 | 287723 | 288325 | + | NG_SM01_00241 | | hypothetical protein | block_1 |
| edge_1 | 289930 | 290619 | + | NG_SM01_00244 | gfcB | putative lipoprotein GfcB | block_1 |
| edge_1 | 290625 | 291377 | + | NG_SM01_00245 | | hypothetical protein | block_1 |
| edge_1 | 333971 | 335200 | - | NG_SM01_00286 | mtnN | 5'-methylthioadenosine/S-adenosylhomocysteine nucleosidase | block_2 |
| edge_1 | 335639 | 339058 | - | NG_SM01_00287 | | hypothetical protein | block_2 |
| edge_1 | 348467 | 348682 | + | NG_SM01_00294 | | hypothetical protein | block_2 |
| edge_1 | 348935 | 349786 | - | NG_SM01_00295 | | hypothetical protein | block_2 |
| edge_1 | 349936 | 350601 | - | NG_SM01_00296 | | hypothetical protein | block_2 |
| edge_1 | 350613 | 350846 | - | NG_SM01_00297 | | putative HTH-type transcriptional regulator | block_2 |
| edge_1 | 350843 | 351622 | - | NG_SM01_00298 | | hypothetical protein | block_2 |
| edge_1 | 435823 | 437064 | + | NG_SM01_00373 | intA | Prophage integrase IntA | block_3 |
| edge_1 | 437294 | 439921 | - | NG_SM01_00374 | | hypothetical protein | block_3 |
| edge_1 | 440653 | 440787 | + | NG_SM01_00375 | | hypothetical protein | block_3 |
| edge_1 | 441523 | 442305 | + | NG_SM01_00376 | | hypothetical protein | block_3 |
| edge_1 | 534676 | 536259 | - | NG_SM01_00466 | | hypothetical protein | block_4 |
| edge_1 | 536522 | 536776 | + | NG_SM01_00467 | | hypothetical protein | block_4 |
| edge_1 | 536870 | 537052 | - | NG_SM01_00468 | | hypothetical protein | block_4 |
| edge_1 | 537118 | 538287 | - | NG_SM01_00469 | | hypothetical protein | block_4 |
| edge_1 | 538562 | 538903 | + | NG_SM01_00470 | | hypothetical protein | block_4 |
| edge_1 | 539705 | 540838 | - | NG_SM01_00471 | | hypothetical protein | block_4 |
| edge_1 | 540960 | 542654 | - | NG_SM01_00472 | | hypothetical protein | block_4 |
| edge_1 | 543000 | 544400 | + | NG_SM01_00473 | | hypothetical protein | block_4 |
| edge_1 | 544476 | 545738 | - | NG_SM01_00474 | | hypothetical protein | block_4 |
| edge_1 | 545748 | 546563 | - | NG_SM01_00475 | yejK | Nucleoid-associated protein YejK | block_4 |
| edge_1 | 547022 | 547255 | - | NG_SM01_00476 | | hypothetical protein | block_4 |
| edge_1 | 547466 | 548233 | + | NG_SM01_00477 | | hypothetical protein | block_4 |
| edge_1 | 548264 | 548584 | + | NG_SM01_00478 | | hypothetical protein | block_4 |
| edge_1 | 548592 | 549053 | + | NG_SM01_00479 | | hypothetical protein | block_4 |
| edge_1 | 549421 | 549657 | + | NG_SM01_00480 | | hypothetical protein | block_4 |
| edge_1 | 549660 | 550604 | - | NG_SM01_00481 | | hypothetical protein | block_4 |
| edge_1 | 649565 | 649777 | + | NG_SM01_00591 | | hypothetical protein | block_5 |
| edge_1 | 1100244 | 1100591 | + | NG_SM01_00998 | | hypothetical protein | block_6 |
| edge_1 | 1100609 | 1101367 | + | NG_SM01_00999 | | hypothetical protein | block_6 |
| edge_1 | 1384036 | 1384965 | + | NG_SM01_01287 | | hypothetical protein | block_7 |
| edge_1 | 1386158 | 1386808 | + | NG_SM01_01289 | | hypothetical protein | block_7 |
| edge_1 | 1512940 | 1513647 | + | NG_SM01_01387 | | hypothetical protein | block_8 |
| edge_1 | 1590070 | 1590663 | - | NG_SM01_01468 | | hypothetical protein | block_9 |
| edge_1 | 1590709 | 1591623 | - | NG_SM01_01469 | | hypothetical protein | block_9 |
| edge_1 | 1594236 | 1596173 | - | NG_SM01_01472 | | hypothetical protein | block_9 |
| edge_1 | 1724719 | 1725663 | + | NG_SM01_01593 | | hypothetical protein | block_10 |
| edge_1 | 1725672 | 1726598 | + | NG_SM01_01594 | | hypothetical protein | block_10 |
| edge_1 | 1726585 | 1727301 | + | NG_SM01_01595 | lnrL | Linearmycin resistance ATP-binding protein LnrL | block_10 |
| edge_1 | 1727298 | 1728434 | + | NG_SM01_01596 | | hypothetical protein | block_10 |
| edge_1 | 1957395 | 1958153 | - | NG_SM01_01799 | | hypothetical protein | block_11 |
| edge_1 | 1985404 | 1985916 | - | NG_SM01_01822 | | hypothetical protein | block_12 |
| edge_1 | 1985931 | 1986251 | - | NG_SM01_01823 | | hypothetical protein | block_12 |
| edge_1 | 2107942 | 2108196 | - | NG_SM01_01924 | | hypothetical protein | block_13 |
| edge_1 | 2249097 | 2250182 | - | NG_SM01_02052 | algL | Alginate lyase | block_14 |
| edge_1 | 2250179 | 2251492 | - | NG_SM01_02053 | | hypothetical protein | block_14 |
| edge_1 | 2251485 | 2252840 | - | NG_SM01_02054 | | hypothetical protein | block_14 |
| edge_1 | 2252870 | 2253640 | - | NG_SM01_02055 | | N-acetylglucosaminyldiphosphoundecaprenol N-acetyl-beta-D-mannosaminyltransferase | block_14 |
| edge_1 | 2253649 | 2254875 | - | NG_SM01_02056 | | hypothetical protein | block_14 |
| edge_1 | 2254872 | 2255993 | - | NG_SM01_02057 | | hypothetical protein | block_14 |
| edge_1 | 2255990 | 2256979 | - | NG_SM01_02058 | | hypothetical protein | block_14 |
| edge_1 | 2393359 | 2394159 | + | NG_SM01_02183 | | hypothetical protein | block_15 |
| edge_1 | 2493490 | 2496792 | + | NG_SM01_02276 | | hypothetical protein | block_16 |
| edge_1 | 2496792 | 2498996 | + | NG_SM01_02277 | | hypothetical protein | block_16 |
| edge_1 | 2498993 | 2499370 | + | NG_SM01_02278 | | hypothetical protein | block_16 |
| edge_1 | 2501174 | 2501929 | - | NG_SM01_02282 | | putative oxidoreductase | block_16 |
| edge_1 | 2502181 | 2502822 | - | NG_SM01_02283 | | hypothetical protein | block_16 |
| edge_1 | 2502897 | 2503469 | - | NG_SM01_02284 | rutB | Peroxyureidoacrylate/ureidoacrylate amidohydrolase RutB | block_16 |
| edge_1 | 2503473 | 2504111 | - | NG_SM01_02285 | | hypothetical protein | block_16 |
| edge_1 | 2504211 | 2504405 | - | NG_SM01_02286 | | hypothetical protein | block_16 |
| edge_1 | 2504563 | 2505726 | + | NG_SM01_02287 | | putative zinc-binding alcohol dehydrogenase | block_16 |
| edge_1 | 2505734 | 2506228 | + | NG_SM01_02288 | | hypothetical protein | block_16 |
| edge_1 | 2506352 | 2506780 | + | NG_SM01_02289 | bluF | Blue light- and temperature-regulated antirepressor BluF | block_16 |
| edge_1 | 2506823 | 2507215 | - | NG_SM01_02290 | | hypothetical protein | block_16 |
| edge_1 | 2507407 | 2507745 | + | NG_SM01_02291 | | hypothetical protein | block_16 |
| edge_1 | 2507910 | 2508431 | + | NG_SM01_02292 | yciE | Protein YciE | block_16 |
| edge_1 | 2508462 | 2509370 | + | NG_SM01_02293 | | hypothetical protein | block_16 |
| edge_1 | 2509491 | 2510978 | + | NG_SM01_02294 | petC_2 | Cytochrome b6-f complex iron-sulfur subunit | block_16 |
| edge_1 | 2511037 | 2511345 | - | NG_SM01_02295 | | hypothetical protein | block_16 |
| edge_1 | 2658613 | 2659584 | + | NG_SM01_02428 | | hypothetical protein | block_17 |
| edge_1 | 2659571 | 2662936 | + | NG_SM01_02429 | | hypothetical protein | block_17 |
| edge_1 | 2662933 | 2663856 | + | NG_SM01_02430 | | hypothetical protein | block_17 |
| edge_1 | 2736258 | 2736461 | + | NG_SM01_02490 | | hypothetical protein | block_18 |
| edge_1 | 2913863 | 2914336 | + | NG_SM01_02644 | | hypothetical protein | block_19 |
| edge_1 | 2963374 | 2963499 | + | NG_SM01_02681 | | hypothetical protein | block_20 |
| edge_1 | 2986066 | 2986182 | - | NG_SM01_02707 | | hypothetical protein | block_21 |
| edge_1 | 3289476 | 3289802 | + | NG_SM01_02969 | | hypothetical protein | block_22 |
| edge_1 | 3332142 | 3333308 | - | NG_SM01_03013 | | hypothetical protein | block_23 |
| edge_1 | 3333305 | 3333496 | - | NG_SM01_03014 | | hypothetical protein | block_23 |
| edge_1 | 3333489 | 3333641 | - | NG_SM01_03015 | | hypothetical protein | block_23 |
| edge_1 | 3333634 | 3333834 | - | NG_SM01_03016 | | hypothetical protein | block_23 |
| edge_1 | 3333831 | 3334952 | - | NG_SM01_03017 | | hypothetical protein | block_23 |
| edge_1 | 3334949 | 3335158 | - | NG_SM01_03018 | | hypothetical protein | block_23 |
| edge_1 | 3335455 | 3335691 | - | NG_SM01_03019 | | hypothetical protein | block_23 |
| edge_1 | 3336052 | 3336456 | + | NG_SM01_03020 | | hypothetical protein | block_23 |
| edge_1 | 3336560 | 3337102 | - | NG_SM01_03021 | | hypothetical protein | block_23 |
| edge_1 | 3337620 | 3337844 | + | NG_SM01_03022 | | hypothetical protein | block_23 |
| edge_1 | 3338201 | 3338398 | - | NG_SM01_03023 | | hypothetical protein | block_23 |
| edge_1 | 3338521 | 3339102 | + | NG_SM01_03024 | | hypothetical protein | block_23 |
| edge_1 | 3339099 | 3339410 | + | NG_SM01_03025 | | hypothetical protein | block_23 |
| edge_1 | 3339433 | 3340359 | + | NG_SM01_03026 | | hypothetical protein | block_23 |
| edge_1 | 3340325 | 3340927 | + | NG_SM01_03027 | | hypothetical protein | block_23 |
| edge_1 | 3340917 | 3341117 | + | NG_SM01_03028 | | hypothetical protein | block_23 |
| edge_1 | 3341117 | 3341335 | + | NG_SM01_03029 | | hypothetical protein | block_23 |
| edge_1 | 3341335 | 3341700 | + | NG_SM01_03030 | | hypothetical protein | block_23 |
| edge_1 | 3341697 | 3342200 | + | NG_SM01_03031 | | hypothetical protein | block_23 |
| edge_1 | 3342197 | 3342463 | + | NG_SM01_03032 | | hypothetical protein | block_23 |
| edge_1 | 3342460 | 3342705 | + | NG_SM01_03033 | | hypothetical protein | block_23 |
| edge_1 | 3342705 | 3343394 | + | NG_SM01_03034 | | hypothetical protein | block_23 |
| edge_1 | 3343603 | 3343836 | + | NG_SM01_03035 | | hypothetical protein | block_23 |
| edge_1 | 3343826 | 3344239 | + | NG_SM01_03036 | | hypothetical protein | block_23 |
| edge_1 | 3344236 | 3344931 | + | NG_SM01_03037 | | hypothetical protein | block_23 |
| edge_1 | 3344972 | 3345445 | + | NG_SM01_03038 | | hypothetical protein | block_23 |
| edge_1 | 3347880 | 3348383 | + | NG_SM01_03043 | | hypothetical protein | block_23 |
| edge_1 | 3348358 | 3350445 | + | NG_SM01_03044 | | hypothetical protein | block_23 |
| edge_1 | 3350447 | 3350662 | + | NG_SM01_03045 | | hypothetical protein | block_23 |
| edge_1 | 3350655 | 3352136 | + | NG_SM01_03046 | | hypothetical protein | block_23 |
| edge_1 | 3352126 | 3353541 | + | NG_SM01_03047 | | hypothetical protein | block_23 |
| edge_1 | 3353543 | 3354232 | + | NG_SM01_03048 | | hypothetical protein | block_23 |
| edge_1 | 3354307 | 3355311 | + | NG_SM01_03049 | | hypothetical protein | block_23 |
| edge_1 | 3355355 | 3356116 | + | NG_SM01_03050 | | hypothetical protein | block_23 |
| edge_1 | 3356113 | 3356442 | + | NG_SM01_03051 | | hypothetical protein | block_23 |
| edge_1 | 3356435 | 3356878 | + | NG_SM01_03052 | | hypothetical protein | block_23 |
| edge_1 | 3356915 | 3357667 | + | NG_SM01_03053 | | hypothetical protein | block_23 |
| edge_1 | 3357670 | 3358089 | + | NG_SM01_03054 | | hypothetical protein | block_23 |
| edge_1 | 3358306 | 3361023 | + | NG_SM01_03055 | | hypothetical protein | block_23 |
| edge_1 | 3361023 | 3361379 | + | NG_SM01_03056 | | hypothetical protein | block_23 |
| edge_1 | 3361376 | 3361837 | + | NG_SM01_03057 | | hypothetical protein | block_23 |
| edge_1 | 3361837 | 3362229 | + | NG_SM01_03058 | | hypothetical protein | block_23 |
| edge_1 | 3362220 | 3370334 | + | NG_SM01_03059 | | hypothetical protein | block_23 |
| edge_1 | 3370413 | 3370619 | + | NG_SM01_03060 | | hypothetical protein | block_23 |
| edge_1 | 3370953 | 3371270 | + | NG_SM01_03061 | | hypothetical protein | block_23 |
| edge_1 | 3371698 | 3371916 | + | NG_SM01_03062 | | hypothetical protein | block_23 |
| edge_1 | 3373358 | 3373801 | + | NG_SM01_03063 | | hypothetical protein | block_23 |
| edge_1 | 3373817 | 3374080 | - | NG_SM01_03064 | | hypothetical protein | block_23 |
| edge_1 | 3374146 | 3374760 | + | NG_SM01_03065 | | hypothetical protein | block_23 |
| edge_1 | 3375655 | 3376176 | + | NG_SM01_03066 | | hypothetical protein | block_23 |
| edge_1 | 3376170 | 3376631 | - | NG_SM01_03067 | | hypothetical protein | block_23 |
| edge_1 | 3376706 | 3377638 | + | NG_SM01_03068 | | hypothetical protein | block_23 |
| edge_1 | 3377953 | 3378396 | + | NG_SM01_03069 | | hypothetical protein | block_23 |
| edge_1 | 3378512 | 3378973 | - | NG_SM01_03070 | | hypothetical protein | block_23 |
| edge_1 | 3633338 | 3634429 | + | NG_SM01_03308 | yhdJ | DNA adenine methyltransferase YhdJ | block_24 |
| edge_1 | 3634399 | 3635403 | - | NG_SM01_03309 | | hypothetical protein | block_24 |
| edge_1 | 3636099 | 3636326 | - | NG_SM01_03310 | | hypothetical protein | block_24 |
| edge_1 | 3636686 | 3637978 | + | NG_SM01_03311 | | hypothetical protein | block_24 |
| edge_1 | 3638661 | 3639188 | - | NG_SM01_03313 | | hypothetical protein | block_24 |
| edge_1 | 3759671 | 3760720 | + | NG_SM01_03426 | | hypothetical protein | block_25 |
| edge_1 | 4034274 | 4034651 | - | NG_SM01_03655 | | hypothetical protein | block_26 |
| edge_1 | 4034685 | 4035065 | - | NG_SM01_03656 | | hypothetical protein | block_26 |
| edge_1 | 4035152 | 4037125 | - | NG_SM01_03657 | macB_3 | Macrolide export ATP-binding/permease protein MacB | block_26 |
| edge_1 | 4037122 | 4038345 | - | NG_SM01_03658 | macA_3 | Macrolide export protein MacA | block_26 |
| edge_1 | 4038477 | 4039256 | - | NG_SM01_03659 | | hypothetical protein | block_26 |
| edge_1 | 4039399 | 4040754 | - | NG_SM01_03660 | sasA_15 | Adaptive-response sensory-kinase SasA | block_26 |
| edge_1 | 4040751 | 4041425 | - | NG_SM01_03661 | rssB_3 | Swarming motility regulation protein RssB | block_26 |
| edge_1 | 4067605 | 4068258 | - | NG_SM01_03683 | ycaC_2 | putative hydrolase YcaC | block_27 |
| edge_1 | 4068399 | 4069310 | + | NG_SM01_03684 | dmlR_10 | HTH-type transcriptional regulator DmlR | block_27 |
| edge_1 | 4071576 | 4072469 | - | NG_SM01_03688 | pgrR_11 | HTH-type transcriptional regulator PgrR | block_27 |
| edge_1 | 4072581 | 4073639 | + | NG_SM01_03689 | | putative protein | block_27 |
| edge_1 | 4073636 | 4074847 | + | NG_SM01_03690 | nepI_2 | Purine ribonucleoside efflux pump NepI | block_27 |
| edge_1 | 4074982 | 4075329 | + | NG_SM01_03691 | | hypothetical protein | block_27 |
| edge_1 | 4075478 | 4076572 | + | NG_SM01_03692 | | hypothetical protein | block_27 |
| edge_1 | 4076726 | 4077784 | + | NG_SM01_03693 | ycjY | putative protein YcjY | block_27 |
| edge_1 | 4077781 | 4078935 | + | NG_SM01_03694 | | hypothetical protein | block_27 |
| edge_1 | 4078948 | 4079841 | - | NG_SM01_03695 | pgrR_12 | HTH-type transcriptional regulator PgrR | block_27 |
| edge_1 | 4080012 | 4080512 | + | NG_SM01_03696 | | hypothetical protein | block_27 |
| edge_1 | 4080523 | 4081596 | + | NG_SM01_03697 | iolS_2 | Aldo-keto reductase IolS | block_27 |
| edge_1 | 4088090 | 4088890 | + | NG_SM01_03703 | | hypothetical protein | block_27 |
| edge_1 | 4088887 | 4089171 | + | NG_SM01_03704 | | hypothetical protein | block_27 |
| edge_1 | 4089161 | 4089298 | + | NG_SM01_03705 | | hypothetical protein | block_27 |
| edge_1 | 4095320 | 4095571 | + | NG_SM01_03716 | | hypothetical protein | block_27 |

**Supplementary Table S14. Block-wise concentration of annotated panel-restricted genes.** Counts of annotated (non-hypothetical) panel-restricted genes per block, ranked by contribution.

| **block_id** | **n_** **annotated panel-restricted genes** |
| --- | --- |
| **block_27** | 8 |
| **block_16** | 6 |
| **block_26** | 4 |
| **block_14** | 2 |
| **block_2** | 2 |
| **block_1** | 1 |
| **block_10** | 1 |
| **block_4** | 1 |
| **block_24** | 1 |
| **block_3** | 1 |

**Supplementary Table S15. Annotated NG-SM01 panel-restricted genes and associated cluster identifiers.** List of non-hypothetical panel-restricted genes with coordinates, functional annotation, and corresponding pangenome cluster IDs.

| **block_id** | **start** | **end** | **strand** | **locus_tag** | **gene** | **product** | **cluster_id** |
| --- | --- | --- | --- | --- | --- | --- | --- |
| block_1 | 289930 | 290619 | + | NG_SM01_00244 | gfcB | putative lipoprotein GfcB | group_5956 |
| block_10 | 1726585 | 1727301 | + | NG_SM01_01595 | lnrL | Linearmycin resistance ATP-binding protein LnrL | group_5678 |
| block_14 | 2249097 | 2250182 | - | NG_SM01_02052 | algL | Alginate lyase | algL |
| block_14 | 2252870 | 2253640 | - | NG_SM01_02055 | | N-acetylglucosaminyldiphosphoundecaprenol N-acetyl-beta-D-mannosaminyltransferase | group_5101 |
| block_16 | 2501174 | 2501929 | - | NG_SM01_02282 | | putative oxidoreductase | group_5251 |
| block_16 | 2502897 | 2503469 | - | NG_SM01_02284 | rutB | Peroxyureidoacrylate/ureidoacrylate amidohydrolase RutB | rutB |
| block_16 | 2504563 | 2505726 | + | NG_SM01_02287 | | putative zinc-binding alcohol dehydrogenase | group_2797 |
| block_16 | 2506352 | 2506780 | + | NG_SM01_02289 | bluF | Blue light- and temperature-regulated antirepressor BluF | group_972 |
| block_16 | 2507910 | 2508431 | + | NG_SM01_02292 | yciE | Protein YciE | group_261 |
| block_16 | 2509491 | 2510978 | + | NG_SM01_02294 | petC_2 | Cytochrome b6-f complex iron-sulfur subunit | petC_2 |
| block_2 | 333971 | 335200 | - | NG_SM01_00286 | mtnN | 5'-methylthioadenosine/S-adenosylhomocysteine nucleosidase | mtnN |
| block_2 | 350613 | 350846 | - | NG_SM01_00297 | | putative HTH-type transcriptional regulator | group_2599 |
| block_24 | 3633338 | 3634429 | + | NG_SM01_03308 | yhdJ | DNA adenine methyltransferase YhdJ | yhdJ |
| block_26 | 4035152 | 4037125 | - | NG_SM01_03657 | macB_3 | Macrolide export ATP-binding/permease protein MacB | macB_3 |
| block_26 | 4037122 | 4038345 | - | NG_SM01_03658 | macA_3 | Macrolide export protein MacA | macA_3 |
| block_26 | 4039399 | 4040754 | - | NG_SM01_03660 | sasA_15 | Adaptive-response sensory-kinase SasA | sasA_15 |
| block_26 | 4040751 | 4041425 | - | NG_SM01_03661 | rssB_3 | Swarming motility regulation protein RssB | rssB_3 |
| block_27 | 4067605 | 4068258 | - | NG_SM01_03683 | ycaC_2 | putative hydrolase YcaC | ycaC_2 |
| block_27 | 4068399 | 4069310 | + | NG_SM01_03684 | dmlR_10 | HTH-type transcriptional regulator DmlR | dmlR_10 |
| block_27 | 4071576 | 4072469 | - | NG_SM01_03688 | pgrR_11 | HTH-type transcriptional regulator PgrR | pgrR_11 |
| block_27 | 4072581 | 4073639 | + | NG_SM01_03689 | | putative protein | group_3502 |
| block_27 | 4073636 | 4074847 | + | NG_SM01_03690 | nepI_2 | Purine ribonucleoside efflux pump NepI | nepI_2 |
| block_27 | 4076726 | 4077784 | + | NG_SM01_03693 | ycjY | putative protein YcjY | group_3503 |
| block_27 | 4078948 | 4079841 | - | NG_SM01_03695 | pgrR_12 | HTH-type transcriptional regulator PgrR | pgrR_12 |
| block_27 | 4080523 | 4081596 | + | NG_SM01_03697 | iolS_2 | Aldo-keto reductase IolS | iolS_2 |
| block_3 | 435823 | 437064 | + | NG_SM01_00373 | intA | Prophage integrase IntA | intA |
| block_4 | 545748 | 546563 | - | NG_SM01_00475 | yejK | Nucleoid-associated protein YejK | group_4695 |

**Supplementary Table S16. Functional and COG context of annotated panel-restricted genes.** Annotated panel-restricted genes with finalized gene names, COG categories, and cluster identifiers to support functional grouping.

| **block_id** | **contig** | **start** | **end** | **strand** | **locus_tag** | | | **gene_name** | **COG_category** | **cluster_id** |
| --- | --- | --- | --- | --- | --- | --- | --- | --- | --- | --- |
| block_1 | edge_1 | 289930 | 290619 | + | NG_SM01_00244 | | | gfcB | S | group_5956 |
| block_10 | edge_1 | 1726585 | 1727301 | + | NG_SM01_01595 | | | lnrL | S | group_5678 |
| block_14 | edge_1 | 2249097 | 2250182 | - | NG_SM01_02052 | | | algL | G | algL |
| block_14 | edge_1 | 2252870 | 2253640 | - | NG_SM01_02055 | |  | | M | group_5101 |
| block_16 | edge_1 | 2501174 | 2501929 | - | NG_SM01_02282 |  | | | M | group_5251 |
| block_16 | edge_1 | 2502897 | 2503469 | - | NG_SM01_02284 | | | rutB | Q | rutB |
| block_16 | edge_1 | 2504563 | 2505726 | + | NG_SM01_02287 | | |  | E | group_2797 |
| block_16 | edge_1 | 2506352 | 2506780 | + | NG_SM01_02289 | | | bluF | T | group_972 |
| block_16 | edge_1 | 2507910 | 2508431 | + | NG_SM01_02292 | | | yciE | S | group_261 |
| block_16 | edge_1 | 2509491 | 2510978 | + | NG_SM01_02294 | | | petC_2 | CE | petC_2 |
| block_2 | edge_1 | 333971 | 335200 | - | NG_SM01_00286 | | | mtnN | F | mtnN |
| block_2 | edge_1 | 350613 | 350846 | - | NG_SM01_00297 | | |  | K | group_2599 |
| block_24 | edge_1 | 3633338 | 3634429 | + | NG_SM01_03308 | | | yhdJ | L | yhdJ |
| block_26 | edge_1 | 4035152 | 4037125 | - | NG_SM01_03657 | | | macB_3 | V | macB_3 |
| block_26 | edge_1 | 4037122 | 4038345 | - | NG_SM01_03658 | | | macA_3 | M | macA_3 |
| block_26 | edge_1 | 4039399 | 4040754 | - | NG_SM01_03660 | | | sasA_15 | T | sasA_15 |
| block_26 | edge_1 | 4040751 | 4041425 | - | NG_SM01_03661 | | | rssB_3 | T | rssB_3 |
| block_27 | edge_1 | 4067605 | 4068258 | - | NG_SM01_03683 | | | ycaC_2 | Q | ycaC_2 |
| block_27 | edge_1 | 4068399 | 4069310 | + | NG_SM01_03684 | | | dmlR_10 | K | dmlR_10 |
| block_27 | edge_1 | 4071576 | 4072469 | - | NG_SM01_03688 | | | pgrR_11 | K | pgrR_11 |
| block_27 | edge_1 | 4072581 | 4073639 | + | NG_SM01_03689 | | |  | S | group_3502 |
| block_27 | edge_1 | 4073636 | 4074847 | + | NG_SM01_03690 | | | nepI_2 | EGP | nepI_2 |
| block_27 | edge_1 | 4076726 | 4077784 | + | NG_SM01_03693 | | | ycjY | S | group_3503 |
| block_27 | edge_1 | 4078948 | 4079841 | - | NG_SM01_03695 | | | pgrR_12 | K | pgrR_12 |
| block_27 | edge_1 | 4080523 | 4081596 | + | NG_SM01_03697 | | | iolS_2 | C | iolS_2 |
| block_3 | edge_1 | 435823 | 437064 | + | NG_SM01_00373 | | | intA | L | intA |
| block_4 | edge_1 | 545748 | 546563 | - | NG_SM01_00475 | | | yejK | S | group_4695 |

**Supplementary Table S17. Composition of panel-restricted genes blocks by hypothetical versus non-hypothetical content.** Per-block totals of panel-restricted genes with hypothetical/non-hypothetical counts and percentages.

| **block** | **contig** | **Start** | **End** | **panel-restricted genes total** | **panel-restricted hypo count** | **panel-restricted hypo %** | **panel-restricted _nonhypo count** | **panel-restricted _nonhypo %** |
| --- | --- | --- | --- | --- | --- | --- | --- | --- |
| block_1 | edge_1 | 286650 | 291377 | 4 | 3 | 75 | 1 | 25 |
| block_10 | edge_1 | 1724719 | 1728434 | 4 | 3 | 75 | 1 | 25 |
| block_11 | edge_1 | 1957395 | 1958153 | 1 | 1 | 100 | 0 | 0 |
| block_12 | edge_1 | 1985404 | 1986251 | 2 | 2 | 100 | 0 | 0 |
| block_13 | edge_1 | 2107942 | 2108196 | 1 | 1 | 100 | 0 | 0 |
| block_14 | edge_1 | 2249097 | 2256979 | 7 | 5 | 71.43 | 2 | 28.57 |
| block_15 | edge_1 | 2393359 | 2394159 | 1 | 1 | 100 | 0 | 0 |
| **block_16** | **edge_1** | **2493490** | **2511345** | **17** | **11** | **64.71** | **6** | **35.29** |
| block_17 | edge_1 | 2658613 | 2663856 | 3 | 3 | 100 | 0 | 0 |
| block_18 | edge_1 | 2736258 | 2736461 | 1 | 1 | 100 | 0 | 0 |
| block_19 | edge_1 | 2913863 | 2914336 | 1 | 1 | 100 | 0 | 0 |
| block_2 | edge_1 | 333971 | 351622 | 7 | 5 | 71.43 | 2 | 28.57 |
| block_20 | edge_1 | 2963374 | 2963499 | 1 | 1 | 100 | 0 | 0 |
| block_21 | edge_1 | 2986066 | 2986182 | 1 | 1 | 100 | 0 | 0 |
| block_22 | edge_1 | 3289476 | 3289802 | 1 | 1 | 100 | 0 | 0 |
| block_23 | edge_1 | 3332142 | 3378973 | 54 | 54 | 100 | 0 | 0 |
| block_24 | edge_1 | 3633338 | 3639188 | 5 | 4 | 80 | 1 | 20 |
| block_25 | edge_1 | 3759671 | 3760720 | 1 | 1 | 100 | 0 | 0 |
| **block_26** | **edge_1** | **4034274** | **4041425** | **7** | **3** | **42.86** | **4** | **57.14** |
| **block_27** | **edge_1** | **4067605** | **4095571** | **16** | **8** | **50** | **8** | **50** |
| block_3 | edge_1 | 435823 | 442305 | 4 | 3 | 75 | 1 | 25 |
| block_4 | edge_1 | 534676 | 550604 | 16 | 15 | 93.75 | 1 | 6.25 |
| block_5 | edge_1 | 649565 | 649777 | 1 | 1 | 100 | 0 | 0 |
| block_6 | edge_1 | 1100244 | 1101367 | 2 | 2 | 100 | 0 | 0 |
| block_7 | edge_1 | 1384036 | 1386808 | 2 | 2 | 100 | 0 | 0 |
| block_8 | edge_1 | 1512940 | 1513647 | 1 | 1 | 100 | 0 | 0 |
| block_9 | edge_1 | 1590070 | 1596173 | 3 | 3 | 100 | 0 | 0 |

**Supplementary Table S18. Enrichment testing for non-hypothetical genes within panel-restricted -gene blocks.**One-sided Fisher’s exact test results per block with odds ratios, *P* values, and Benjamini–Hochberg FDR correction identifying blocks enriched for annotated genes.

| **Block** | **non-hypo** | **hypo** | **Total** | **Odds ratio  (non-hypo enrich)** | | **P (one-sided)** | **BH-FDR** |
| --- | --- | --- | --- | --- | --- | --- | --- |
| **block_27** | **8** | **8** | **16** | | **6.79** | **0.00103** | **0.0279** |
| block_26 | 4 | 3 | 7 | | 7.77 | 0.01466 | 0.1979 |
| block_16 | 6 | 11 | 17 | | 3.27 | 0.03863 | 0.3477 |

**Supplementary Table S19. Pangenome rarefaction and gene discovery across sampling depth.**Mean ± SD pangenome size (gene clusters) and newly discovered gene clusters as a function of the number of genomes sampled (1–20), computed across 500 random genome-order permutations.

| **Genomes_n** | **Pangenome_Mean** | **Pangenome_SD** | **New_Genes_Mean** | **New_Genes_SD** |
| --- | --- | --- | --- | --- |
| 1 | 4092.12 | 256.13 | 4092.12 | 256.13 |
| 2 | 4879.39 | 221.92 | 787.27 | 263.14 |
| 3 | 5459.34 | 249.47 | 579.95 | 199.26 |
| 4 | 5976.57 | 274.79 | 517.22 | 169.67 |
| 5 | 6450.58 | 293.74 | 474.01 | 170.45 |
| 6 | 6884.45 | 297.88 | 433.87 | 160.1 |
| 7 | 7280.32 | 296.77 | 395.87 | 161.82 |
| 8 | 7657.48 | 297.59 | 377.16 | 166.07 |
| 9 | 8018.15 | 300.44 | 360.67 | 161.5 |
| 10 | 8358.1 | 298.33 | 339.95 | 156.14 |
| 11 | 8685.9 | 290.72 | 327.8 | 160.69 |
| 12 | 8994.65 | 287 | 308.75 | 146.2 |
| 13 | 9298.35 | 274.11 | 303.7 | 142.04 |
| 14 | 9575.16 | 266.56 | 276.81 | 138.7 |
| 15 | 9850.11 | 250.19 | 274.95 | 139.8 |
| 16 | 10106.12 | 228.26 | 256.01 | 138.21 |
| 17 | 10367.74 | 199.54 | 261.62 | 138.09 |
| 18 | 10610.94 | 162.91 | 243.2 | 129.49 |
| 19 | 10852.35 | 121.8 | 241.41 | 124.11 |
| 20 | 11082 | 0 | 229.65 | 121.8 |

**Supplementary Table S20. Heaps’ law estimates support an open pangenome across nested sampling depths.**Heaps’ law α estimates with 95% confidence intervals across nested genome counts (8–20), along with corresponding mean pangenome totals and mean ± SD of newly discovered clusters at the last genome addition, derived from permutation-based rarefaction.

| **Genomes_n** | **Heaps_alpha** | **alpha_CI_low** | **alpha_CI_high** | **Pangenome_total** | **Pangenome_SD** | **New_genes_last_mean** | **New_genes_last_SD** |
| --- | --- | --- | --- | --- | --- | --- | --- |
| 8 | 0.3031 | 0.2368 | 0.3773 | 7629.925 | 304.364 | 371.945 | 166.79 |
| 10 | 0.3105 | 0.2534 | 0.3738 | 8276.05 | 304.326 | 334.729 | 152.955 |
| 12 | 0.3229 | 0.2686 | 0.3731 | 8972 | 254.814 | 313.602 | 148.391 |
| 14 | 0.3309 | 0.2801 | 0.3784 | 9584.488 | 288.745 | 288.94 | 143.347 |
| 16 | 0.3364 | 0.2897 | 0.3784 | 10093.36 | 241.431 | 264.795 | 138.625 |
| 18 | 0.3431 | 0.2999 | 0.3809 | 10614.96 | 194.026 | 247.85 | 130.582 |
| 20 | 0.3479 | 0.3081 | 0.3814 | 11082 | 0 | 230.276 | 124.095 |

**Supplementary Table S21. CAZyme class composition across the NG-SM01–centered *S. maltophilia* complex panel (n = 20 genomes).** Per-genome CAZyme copy numbers are reported for the six major CAZy classes (GH, GT, CE, PL, AA, CBM) with class-wise proportions in parentheses (% of total CAZymes per genome). TOTAL indicates the summed CAZyme count per genome. NG-SM01 is shown alongside 19 reference genomes to enable direct cross-genome comparison of class-level abundance and relative composition.

| **genome** | **GH** | **GT** | **CE** | **PL** | **AA** | **CBM** | **TOTAL** |
| --- | --- | --- | --- | --- | --- | --- | --- |
| NG-SM01 | 47 (45.6%) | 29 (28.2%) | 12 (11.7%) | 1 (1.0%) | 6 (5.8%) | 8 (7.8%) | 103 |
| GCF_000020665.1 | 34 (35.8%) | 31 (32.6%) | 16 (16.8%) | 2 (2.1%) | 7 (7.4%) | 5 (5.3%) | 95 |
| GCF_001274595.1 | 37 (38.5%) | 28 (29.2%) | 17 (17.7%) | 1 (1.0%) | 8 (8.3%) | 5 (5.2%) | 96 |
| GCF_001431665.1 | 40 (38.1%) | 29 (27.6%) | 18 (17.1%) | 2 (1.9%) | 8 (7.6%) | 8 (7.6%) | 105 |
| GCF_001676385.1 | 44 (42.7%) | 25 (24.3%) | 17 (16.5%) | 3 (2.9%) | 8 (7.8%) | 6 (5.8%) | 103 |
| GCF_002138415.1 | 40 (38.1%) | 29 (27.6%) | 17 (16.2%) | 3 (2.9%) | 8 (7.6%) | 8 (7.6%) | 105 |
| GCF_002799155.1 | 32 (35.6%) | 27 (30.0%) | 18 (20.0%) | 1 (1.1%) | 7 (7.8%) | 5 (5.6%) | 90 |
| GCF_002799165.1 | 36 (36.7%) | 28 (28.6%) | 18 (18.4%) | 2 (2.0%) | 9 (9.2%) | 5 (5.1%) | 98 |
| GCF_002799245.1 | 33 (37.1%) | 25 (28.1%) | 16 (18.0%) | 1 (1.1%) | 10 (11.2%) | 4 (4.5%) | 89 |
| GCF_003205835.1 | 31 (34.4%) | 27 (30.0%) | 17 (18.9%) | 1 (1.1%) | 9 (10.0%) | 5 (5.6%) | 90 |
| GCF_004346925.1 | 33 (35.5%) | 29 (31.2%) | 17 (18.3%) | 1 (1.1%) | 8 (8.6%) | 5 (5.4%) | 93 |
| GCF_004684085.1 | 32 (33.7%) | 30 (31.6%) | 15 (15.8%) | 2 (2.1%) | 11 (11.6%) | 5 (5.3%) | 95 |
| GCF_004920835.1 | 35 (35.7%) | 31 (31.6%) | 18 (18.4%) | 2 (2.0%) | 7 (7.1%) | 5 (5.1%) | 98 |
| GCF_006970445.1 | 33 (35.9%) | 27 (29.3%) | 19 (20.7%) | 1 (1.1%) | 7 (7.6%) | 5 (5.4%) | 92 |
| GCF_006974125.1 | 34 (36.6%) | 28 (30.1%) | 16 (17.2%) | 1 (1.1%) | 8 (8.6%) | 6 (6.5%) | 93 |
| GCF_013004645.1 | 41 (39.0%) | 29 (27.6%) | 19 (18.1%) | 2 (1.9%) | 8 (7.6%) | 6 (5.7%) | 105 |
| GCF_013464915.1 | 35 (38.5%) | 28 (30.8%) | 16 (17.6%) | 0 (0.0%) | 7 (7.7%) | 5 (5.5%) | 91 |
| GCF_025642255.1 | 48 (46.6%) | 27 (26.2%) | 14 (13.6%) | 0 (0.0%) | 6 (5.8%) | 8 (7.8%) | 103 |
| GCF_900186865.1 | 41 (39.0%) | 28 (26.7%) | 17 (16.2%) | 3 (2.9%) | 9 (8.6%) | 7 (6.7%) | 105 |
| NBRC_14161 | 42 (39.6%) | 28 (26.4%) | 17 (16.0%) | 3 (2.8%) | 9 (8.5%) | 7 (6.6%) | 106 |

**Supplementary Table S22. Summary statistics for CAZyme class abundance and fraction across genomes.** For each CAZy class (GH, GT, CE, PL, AA, CBM) and the TOTAL CAZyme burden, the table reports (i) the mean ± SD and range of copy number per genome, and (ii) the mean ± SD and range of the fraction across genomes (percentage contribution of each class to the CAZyme repertoire per genome). These summary metrics quantify both absolute and compositional variation across the 20-genome panel.

| **CAZyme class** | **Count per genome (mean ± SD; range)** | | **Fraction across genomes (mean ± SD; range)** |
| --- | --- | --- | --- |
| GH | 37.4 ± 5.1 (31–48) | 38.1% ± 3.4% (33.7–46.6%) | |
| GT | 28.1 ± 1.6 (25–31) | 28.9% ± 2.1% (24.3–32.6%) | |
| CE | 16.7 ± 1.7 (12–19) | 17.2% ± 2.0% (11.7–20.7%) | |
| PL | 1.6 ± 0.9 (0–3) | 1.6% ± 0.9% (0.0–2.9%) | |
| AA | 8.0 ± 1.3 (6–11) | 8.2% ± 1.5% (5.8–11.6%) | |
| CBM | 5.9 ± 1.3 (4–8) | 6.0% ± 1.0% (4.5–7.8%) | |
| **TOTAL** | **97.8 ± 6.1 (89–106)** | **—** | |

**Supplementary Table S23. Comparative gene neighborhood of the rare GT26-associated glycan locus in NG-SM01 and the only other GT26-positive genome.** Gene-order “synteny strip” for the GT26 locus region in NG-SM01 (contig edge_1) and in *S. maltophilia* reference genome GCF_004920835.1 (contig NZ_QFHO01000004.1). For each genome, the table lists contig, gene coordinates, strand, locus tag, and product annotation. Conserved functional anchors (e.g., Wzc/Ugd/GT26) define a shared backbone, while differences highlight locus-level remodeling, including embedding of AlgL/PL5 within the NG-SM01 neighborhood.

| **Genome** | **Contig** | **Gene start** | **Gene end** | **Strand** | **Locus tag** | **Annotation (product)** |
| --- | --- | --- | --- | --- | --- | --- |
| NG-SM01 | edge_1 | 2,244,351 | 2,247,326 | + | NG_SM01_02049 | Vitamin B12 transporter BtuB |
| NG-SM01 | edge_1 | 2,247,392 | 2,248,747 | + | NG_SM01_02050 | Alpha-N-acetylgalactosaminidase |
| NG-SM01 | edge_1 | 2,249,097 | 2,250,182 | − | NG_SM01_02052 | Alginate lyase (AlgL/PL5) |
| NG-SM01 | edge_1 | 2,252,870 | 2,253,640 | − | NG_SM01_02055 | GT26: N-acetylglucosaminyldiphosphoundecaprenol N-acetyl-β-D-mannosaminyltransferase |
| NG-SM01 | edge_1 | 2,257,509 | 2,259,743 | + | NG_SM01_02059 | Tyrosine-protein kinase Wzc |
| NG-SM01 | edge_1 | 2,259,830 | 2,260,996 | − | NG_SM01_02060 | UDP-glucose 6-dehydrogenase (Ugd) |
| NG-SM01 | edge_1 | 2,261,158 | 2,262,051 | + | NG_SM01_02061 | 4-hydroxybenzoate octaprenyltransferase |
| NG-SM01 | edge_1 | 2,262,157 | 2,263,581 | + | NG_SM01_02062 | Putative short-chain fatty acid transporter |
| GCF_004920835.1 | NZ_QFHO01000004.1 | 132,393 | 133,286 | − | GCF_00492083_03252 | 4-hydroxybenzoate octaprenyltransferase |
| GCF_004920835.1 | NZ_QFHO01000004.1 | 133,385 | 135,598 | − | GCF_00492083_03253 | Tyrosine-protein kinase Wzc |
| GCF_004920835.1 | NZ_QFHO01000004.1 | 136,017 | 137,183 | + | GCF_00492083_03254 | UDP-glucose 6-dehydrogenase (Ugd) |
| GCF_004920835.1 | NZ_QFHO01000004.1 | 137,214 | 138,335 | + | GCF_00492083_03255 | GDP-mannose 4,6-dehydratase |
| GCF_004920835.1 | NZ_QFHO01000004.1 | 142,099 | 143,283 | + | GCF_00492083_03259 | Phosphatidyl-myo-inositol mannosyltransferase |
| GCF_004920835.1 | NZ_QFHO01000004.1 | 143,252 | 144,037 | + | GCF_00492083_03260 | GT26: N-acetylglucosaminyldiphosphoundecaprenol N-acetyl-β-D-mannosaminyltransferase |
| GCF_004920835.1 | NZ_QFHO01000004.1 | 144,151 | 144,516 | + | GCF_00492083_03261 | GDP-mannose mannosyl hydrolase |
| GCF_004920835.1 | NZ_QFHO01000004.1 | 144,554 | 145,498 | − | GCF_00492083_03262 | GDP-L-fucose synthase |
| GCF_004920835.1 | NZ_QFHO01000004.1 | 146,856 | 148,211 | − | GCF_00492083_03266 | Alpha-N-acetylgalactosaminidase |
| GCF_004920835.1 | NZ_QFHO01000004.1 | 148,277 | 151,252 | − | GCF_00492083_03267 | Vitamin B12 transporter BtuB |

**Supplementary Table S24.** **Final non-redundant NG-SM01 genomic islands after merging overlapping caller intervals (coordinates, contig, and length).**

| **GI** | **Contig** | **Start (bp)** | **End (bp)** | **Length (bp)** |
| --- | --- | --- | --- | --- |
| GI_1 | edge_1 | 189,712 | 195,476 | 5,765 |
| GI_2 | edge_1 | 333,493 | 339,058 | 5,566 |
| GI_3 | edge_1 | 433,467 | 447,632 | 14,166 |
| GI_4 | edge_1 | 532,797 | 558,795 | 25,999 |
| GI_5 | edge_1 | 1,587,313 | 1,591,623 | 4,311 |
| GI_6 | edge_1 | 2,249,097 | 2,260,996 | 11,900 |
| GI_7 | edge_1 | 2,658,613 | 2,663,856 | 5,244 |
| GI_8 | edge_1 | 3,333,634 | 3,340,359 | 6,726 |
| GI_9 | edge_1 | 3,342,197 | 3,346,753 | 4,557 |
| GI_10 | edge_1 | 3,357,670 | 3,362,229 | 4,560 |
| GI_11 | edge_1 | 3,370,413 | 3,380,024 | 9,612 |
| GI_12 | edge_1 | 3,633,338 | 3,639,188 | 5,851 |
| GI_13 | edge_1 | 3,733,138 | 3,737,626 | 4,489 |
| GI_14 | edge_1 | 3,756,082 | 3,760,720 | 4,639 |
| GI_15 | edge_1 | 3,794,776 | 3,798,972 | 4,197 |
| GI_16 | edge_1 | 4,052,119 | 4,065,246 | 13,128 |
| GI_17 | edge_1 | 4,070,618 | 4,076,572 | 5,955 |
| GI_18 | edge_1 | 4,091,367 | 4,095,571 | 4,205 |

**Supplementary Table S25.** **GI-level nucleotide composition for prioritized islands (length, G/C counts, GI GC%, genome GC%, ΔGC, |ΔGC|, and GC skew).**

| **GI (window)** | **Length (bp)** | **G** | **C** | **GC% (GI)** | **GC% (genome)** | **ΔGC (GI−genome)** | **\|ΔGC\|** | **GC skew** |
| --- | --- | --- | --- | --- | --- | --- | --- | --- |
| GI_4::edge_1:532796–558795 | 25,999 | 7,210 | 8,279 | 59.58 | 66.9 | −7.32 | 7.32 | −0.069017 |
| GI_6::edge_1:2249096–2260996 | 11,900 | 3,745 | 3,438 | 60.36 | 66.9 | −6.54 | 6.54 | 0.04274 |
| GI_3::edge_1:433466–447632 | 14,166 | 4,279 | 4,603 | 62.7 | 66.9 | −4.20 | 4.2 | −0.036478 |
| GI_17::edge_1:4070617–4076572 | 5,955 | 1,897 | 1,849 | 62.91 | 66.9 | −3.99 | 3.99 | 0.012814 |
| GI_16::edge_1:4052118–4065246 | 13,128 | 4,469 | 4,272 | 66.58 | 66.9 | −0.32 | 0.32 | 0.022537 |

**Supplementary Table S26.** **Ranked compositional discordance of prioritized islands using ΔGC, tetranucleotide cosine distance, codon cosine distance, and codon Jensen–Shannon divergence.**

| **GI** | **Length (bp)** | **ΔGC (%)** | **Tetramer cosine distance** | **Codon cosine distance** | **Codon JS divergence (bits)** |
| --- | --- | --- | --- | --- | --- |
| GI_4 | 25,999 | −7.32 | 0.075008 | 0.074304 | 0.049802 |
| GI_6 | 11,900 | −6.54 | 0.061411 | 0.054046 | 0.038696 |
| GI_3 | 14,166 | −4.20 | 0.043129 | 0.052703 | 0.029226 |
| GI_17 | 5,955 | −3.99 | 0.039044 | 0.032176 | 0.026862 |
| GI_16 | 13,128 | −0.32 | 0.009909 | 0.011231 | 0.008973 |

**Supplementary Table S27.** **CDS-level sequence-composition and codon-bias signatures for prioritized islands (CDS GC%, CpG odds, ApT odds, dinucleotide signature distance, codon-usage JSD, ENC).**

| **GI** | **CDS_n** | **CDS GC (%)** | **CpG odds** | **ApT odds** | **Dinuc signature distance** | **Codon-usage JSD** | **ENC** |
| --- | --- | --- | --- | --- | --- | --- | --- |
| GI_3 | 9 | 63.65 | 1.133 | 1.103 | 0.678 | 0.0291 | 85.22 |
| GI_4 | 24 | 60.25 | 1.032 | 1.127 | 0.849 | 0.0498 | 165.83 |
| GI_6 | 9 | 60.37 | 1.092 | 1.267 | 0.632 | 0.0388 | 106.66 |
| GI_16 | 12 | 66.69 | 1.093 | 1.397 | 0.365 | 0.0088 | 61.12 |
| GI_17 | 6 | 63.29 | 1.009 | 1.139 | 0.667 | 0.0269 | 88.69 |

**Supplementary Table S28.** **Ranked island divergence summary across multiple signatures (codon-usage JSD, dinucleotide distance, ApT odds, CpG odds) highlighting the highest-to-lowest discordance order.**

| **Rank** | **Metric** | **GI (highest → lowest)** | **Values** |
| --- | --- | --- | --- |
| 1 | Codon-usage JSD | GI_4 > GI_6 > GI_3 > GI_17 > GI_16 | 0.0498, 0.0388, 0.0291, 0.0269, 0.0088 |
| 2 | Dinuc signature distance | GI_4 > GI_3 > GI_17 > GI_6 > GI_16 | 0.849, 0.678, 0.667, 0.632, 0.365 |
| 3 | ApT odds | GI_16 > GI_6 > GI_17 > GI_4 > GI_3 | 1.397, 1.267, 1.139, 1.127, 1.103 |
| 4 | CpG odds | GI_3 > GI_16 > GI_6 > GI_4 > GI_17 | 1.133, 1.093, 1.092, 1.032, 1.009 |

**Supplementary Table S29.** **Prioritized GI boundaries and flanking (border) genes in NG-SM01 (left/right border products, strand, and coordinates).**

| **GI** | **Contig** | **Start** | **End** | **Left border gene  (product, strand, coords)** | **Right border gene  (product, strand, coords)** |
| --- | --- | --- | --- | --- | --- |
| GI_3 | edge_1 | 433466 | 447632 | NG_SM01_00368 (hypothetical protein, −, 433206–433463) | NG_SM01_00379 (Extracellular basic protease, +, 448028–449527) |
| GI_4 | edge_1 | 532796 | 558795 | NG_SM01_00464 (Non-hemolytic phospholipase C, +, 530622–532730) | NG_SM01_00490 (putative protein kinase UbiB, −, 558885–560570) |
| GI_6 | edge_1 | 2249096 | 2260996 | NG_SM01_02050 (Alpha-N-acetylgalactosaminidase, +, 2247392–2248747) | NG_SM01_02061 (4-hydroxybenzoate octaprenyltransferase, +, 2261158–2262051) |
| GI_16 | edge_1 | 4052118 | 4065246 | NG_SM01_03669 (Vitamin B12 transporter BtuB, −, 4048499–4051210) | NG_SM01_03682 (ATM1-type heavy metal exporter, +, 4065924–4067582) |
| GI_17 | edge_1 | 4070617 | 4076572 | NG_SM01_03686 (hypothetical protein, −, 4070258–4070536) | NG_SM01_03693 (putative protein YcjY, +, 4076726–4077784) |

**Supplementary Table S30.** **First and last CDS fully captured within each prioritized GI (products, strand, and coordinates).**

| **GI** | **GI first gene (product, strand, coords)** | **GI last gene (product, strand, coords)** |
| --- | --- | --- |
| GI_3 | NG_SM01_00369 (Persistence and stress-resistance toxin PasT, −, 433467–433892) | NG_SM01_00378 (hypothetical protein, +, 447420–447632) |
| GI_4 | NG_SM01_00465 (hypothetical protein, −, 532797–533717) | NG_SM01_00489 (LexA repressor, −, 558160–558795) |
| GI_6 | NG_SM01_02052 (Alginate lyase, −, 2249097–2250182) | NG_SM01_02060 (UDP-glucose 6-dehydrogenase, −, 2259830–2260996) |
| GI_16 | NG_SM01_03671 (Aldose 1-epimerase, −, 4052551–4053693) | NG_SM01_03680 (hypothetical protein, −, 4061953–4063332) |
| GI_17 | NG_SM01_03687 (hypothetical protein, −, 4070618–4070776) | NG_SM01_03692 (hypothetical protein, +, 4075478–4076572) |

**Supplementary Table S31.** **Mobility-associated features near GI edges (tRNA at left/right edge; integrase/recombinase near edges).**

| **GI** | **tRNA near left edge (coords)** | **tRNA near right edge (coords)** | | **Integrase/recombinase near edge (product, strand, coords)** |
| --- | --- | --- | --- | --- |
| GI_3 | transfer-messenger RNA, SsrA (435307–435660) | – | NG_SM01_00373 (Prophage integrase IntA, +, 435823–437064) | |
| GI_4 | – | – | – | |
| GI_6 | tRNA-Arg(ccg) (2248935–2249011) | – | – | |
| GI_16 | – | – | – | |
| GI_17 | – | – | – | |

**Supplementary Table S32.** **Mobility/stabilization genes identified within GI_3 (e.g., PasT toxin–antitoxin and integrase IntA) with locus tags and coordinates.**

| **GI** | **Locus tag** | **Gene** | **Product** | **Mobility class** | **Strand** | **CDS start** | **CDS end** |
| --- | --- | --- | --- | --- | --- | --- | --- |
| GI_3 | NG_SM01_00369 | pasT | Persistence and stress-resistance toxin PasT | toxin–antitoxin | − | 433,467 | 433,892 |
| GI_3 | NG_SM01_00373 | intA | Prophage integrase IntA | integrase/recombinase | + | 435,823 | 437,064 |

**Supplementary Table S33.** **Motif-level details for detected boundary repeat candidates (repeat length, mismatches, motifs, flank positions, interpretation).**

| **GI** | **Best hit type** | **Repeat length (bp)** | **Mismatches** | **Left motif** | **Right motif** | **Left pos in L flank (bp)** | **Right pos in R flank (bp)** | **Interpretation** |
| --- | --- | --- | --- | --- | --- | --- | --- | --- |
| GI_6 | near repeat (10 bp, ≤1 mismatch) | 10 | 1 | TTGGCCGCAT | TTCGCCGCAT | 116 | 181 | Weak/degenerate direct-repeat candidate (1 mismatch) consistent with imperfect att-like duplication; supports GI boundary in addition to tRNA adjacency. |
| GI_17 | exact repeat + near repeat | 9 | 0 | CGCTGTTGG | CGCTGTTGG | 28 | 175 | Exact 9-bp direct repeat consistent with short target-site duplication/att-like motif; strengthens boundary confidence. |
| GI_17 | near repeat (10 bp, ≤1 mismatch) | 10 | 1 | CGCGGTTGGC | CGCTGTTGGC | 10 | 175 | Additional weak 10-bp near-repeat (1 mismatch) adjacent to the exact repeat; consistent with localized duplication/degeneracy at boundary. |

**Supplementary Table S34.** **RNA/tRNA and mobility features within ±20 kb of prioritized GI edges (coordinates and contextual signals).**

| **GI** | **Start** | **End** | **RNA/tRNA within 20 kb of left edge** | **Left RNA coords** | **RNA/tRNA within 20 kb of right edge** | **Right RNA coords** | **Integrase/recombinase within 20 kb** | **Feature coords** | **Strand** | **Product** |
| --- | --- | --- | --- | --- | --- | --- | --- | --- | --- | --- |
| GI_16 | 4,052,118 | 4,065,246 | – | – | – | – | – | – | – | – |
| GI_4 | 532,796 | 558,795 | tRNA-Arg(acg) | 522,640–522,716 | tRNA-Ser(gct) | 553,034–553,126 | – | – | – | – |
| GI_17 | 4,070,617 | 4,076,572 | – | – | – | – | – | – | – | – |

**Supplementary Table S35.** **Summary of edge-associated signals from the ±20 kb scan (tRNA/RNA presence, mobility enzyme detection, notes).**

| **GI** | **tRNA/RNA signal near edges (±20 kb)** | **Mobility enzyme (integrase/recombinase) detected (±20 kb)** | **Notes** |
| --- | --- | --- | --- |
| GI_16 | None detected | None detected | No edge-proximal tRNA/RNA or mobility enzyme in the ±20 kb window. |
| GI_4 | Present at both edges (tRNA-Arg(acg) left; tRNA-Ser(gct) right) | None detected | Dual tRNA proximity may indicate preferred integration neighborhood despite no integrase hit in the ±20 kb scan. |
| GI_17 | None detected | None detected | No edge-proximal tRNA/RNA or mobility enzyme in the ±20 kb window. |

**Supplementary Table S36.** **Direct-repeat (att-like/target-site duplication) signals detected at GI boundaries using 200-bp flank scanning (hit type, mismatch, interpretation).**

| **GI** | **Repeat search window** | **Best hit type** | **Repeat length (bp)** | **Mismatches** | **Interpretation** |
| --- | --- | --- | --- | --- | --- |
| GI_3 | 200 bp flanks | LCS exact ≥12 / exact ≥8 / near(10, ≤1 mismatch) | – | – | No detectable direct-repeat signal in 200-bp windows; boundary likely degenerate/short or outside scanned region. Integration support should rely on integrase/tRNA evidence. |
| GI_4 | 200 bp flanks | LCS exact ≥12 / exact ≥8 / near(10, ≤1 mismatch) | – | – | No detectable direct-repeat signal in 200-bp windows; boundary likely degenerate/short or outside scanned region. |
| GI_6 | 200 bp flanks | near repeat (10 bp, ≤1 mismatch) | 10 | 1 | Weak/degenerate direct-repeat candidate (1 mismatch) consistent with imperfect att-like duplication; supports GI boundary in addition to tRNA adjacency. |
| GI_16 | 200 bp flanks | LCS exact ≥12 / exact ≥8 / near(10, ≤1 mismatch) | – | – | No detectable direct-repeat signal in 200-bp windows; boundary likely degenerate/short or outside scanned region. |
| GI_17 | 200 bp flanks | exact repeat + near repeat | 9 | 0 | Exact 9-bp direct repeat consistent with short target-site duplication/att-like motif; strengthens boundary confidence. |

**Supplementary Table S37A.** **Read-mapping coverage depth within each prioritized GI and its flanks, including GI-to-flank enrichment ratios.**

| **GI** | **GI_region** | **GI_mean depth** | **left_flank region** | **left_flank_ mean depth** | **right_flank region** | **right_flank mean depth** | **GI_to_flank ratio** |
| --- | --- | --- | --- | --- | --- | --- | --- |
| GI_3 | edge_1:433467-447632 | 355.42 | edge_1:431467-433466 | 316.77 | edge_1:447633-449632 | 320.62 | 1.115236 |
| GI_4 | edge_1:532797-558795 | 387.42 | edge_1:530797-532796 | 326.8 | edge_1:558796-560795 | 333.11 | 1.17416 |
| GI_6 | edge_1:2249097-2260996 | 460.24 | edge_1:2247097-2249096 | 418.38 | edge_1:2260997-2262996 | 400.8 | 1.12366 |
| GI_16 | edge_1:4052119-4065246 | 324.8 | edge_1:4050119-4052118 | 329.59 | edge_1:4065247-4067246 | 329.27 | 0.985945 |
| GI_17 | edge_1:4070618-4076572 | 325.78 | edge_1:4068618-4070617 | 306.52 | edge_1:4076573-4078572 | 346.53 | 0.997718 |

**Supplementary Table S37B.** **Boundary-spanning read support at left and right GI junctions (counts per boundary).**

| **GI** | **left_boundary_spanning_reads** | **right_boundary_spanning_reads** |
| --- | --- | --- |
| GI_3 | 272 | 374 |
| GI_4 | 364 | 307 |
| GI_6 | 451 | 488 |
| GI_16 | 356 | 366 |
| GI_17 | 277 | 358 |

**Supplementary Table S38.** **Curated-panel nucleotide prevalence of priority genomic islands based on best BLASTn hit (external hit status, best subject, percent identity, and query coverage).**

| **GI** | **External hit in curated panel** | **Best subject** | **pid (%)** | **qcov (%)** |
| --- | --- | --- | --- | --- |
| GI_16 | Yes | GCF_025642255.1 | 99.17 | 100 |
| GI_3 | Yes (partial) | GCF_001676385.1 | 96.3 | 65 |
| GI_4 | No | – | – | – |
| GI_6 | No | – | – | – |
| GI_17 | No | – | – | – |

**Supplementary Table S39.** **Protein-level conservation and locus architecture stability for priority islands, summarizing reciprocal best hits (RBH) and gene-order conservation (adjacent pairs tested vs. conserved) against the selected reference windows.**

| **GI** | **Reference used** | **GI_CDS_total** | **RBH_pairs** | **Adjacent pairs tested** | **Adjacent pairs conserved** | **Adjacency conservation rate** |
| --- | --- | --- | --- | --- | --- | --- |
| GI_16 | GCF_025642255.1 | 12 | 12 | 11 | 11 | 1 |
| GI_3 | GCF_001676385.1 | 9 | 7 | 4 | 0 | 0 |

**Supplementary Table S40.** **Presence/absence classification of prioritized GIs across the 19-genome comparative panel (ABSENT/PARTIAL/PRESENT counts and percentages) using predefined BLAST similarity thresholds.**

| **GI_short** | **ABSENT** | **PARTIAL** | **PRESENT** | **Total** | **PRESENT (%)** | **PARTIAL (%)** | **ABSENT (%)** |
| --- | --- | --- | --- | --- | --- | --- | --- |
| GI_3 | 14 | 5 | 0 | 19 | 0 | 26.3 | 73.7 |
| GI_4 | 2 | 17 | 0 | 19 | 0 | 89.5 | 10.5 |
| GI_6 | 19 | 0 | 0 | 19 | 0 | 0 | 100 |
| GI_16 | 18 | 0 | 1 | 19 | 5.3 | 0 | 94.7 |
| GI_17 | 19 | 0 | 0 | 19 | 0 | 0 | 100 |

*Presence/absence calls were assigned using BLAST-based similarity thresholds as follows: **PRESENT** = identity ≥90% and query coverage (qcov) ≥70%; **PARTIAL** = identity ≥90% and qcov 10–69% (shared fragments); **ABSENT** = otherwise, including **NO_HIT**.

**Supplementary Table S41. COG functional category composition of GI_4 and GI_6.** Counts and percentages of eggNOG-assigned COG categories across GI_4 and GI_6 CDS. GI_4 is dominated by replication/recombination/repair (L, 33.3%) and information-processing categories (J and K, 16.7% each), with additional function-unknown (S, 25.0%) and defense mechanisms (V, 8.3%). GI_6 shows enrichment for cell wall/membrane/envelope biogenesis (M, 33.3%) and function-unknown (S, 33.3%), with additional contributions from cell cycle/chromosome partitioning (D, 11.1%), carbohydrate transport/metabolism (G, 11.1%), and intracellular trafficking/secretion (U, 11.1%).

| **GI** | **COG** | **Count (n)** | **Percent (%)** | | **COG_name** |
| --- | --- | --- | --- | --- | --- |
| GI_4 | L | 4 | 33.3 | Replication, recombination and repair | |
| GI_4 | S | 3 | 25 | Function unknown | |
| GI_4 | J | 2 | 16.7 | Translation, ribosomal structure and biogenesis | |
| GI_4 | K | 2 | 16.7 | Transcription | |
| GI_4 | V | 1 | 8.3 | Defense mechanisms | |
| GI_6 | M | 3 | 33.3 | Cell wall/membrane/envelope biogenesis | |
| GI_6 | S | 3 | 33.3 | Function unknown | |
| GI_6 | D | 1 | 11.1 | Cell cycle control, cell division, chromosome partitioning | |
| GI_6 | G | 1 | 11.1 | Carbohydrate transport and metabolism | |
| GI_6 | U | 1 | 11.1 | Intracellular trafficking, secretion, and vesicular transport | |

**Supplementary Table S42. Key prophage hallmark genes annotated in Region R2 (edge_1:3,343,826–3,370,334).** Pharokka-derived annotation of representative structural and functional markers defining the R2 head/packaging module (terminase small/large subunits, portal protein, head maturation protease, head–tail adaptor, major head protein), tail morphogenesis components (minor/major tail proteins including an Ig-like domain–containing major tail protein, tail assembly chaperone, tape-measure protein, central tail fiber J), and lysis functions (endolysin and Rz-like spanin). Coordinates are reported as genome positions on contig edge_1 with strand orientation.

| **Prophage_region** | **CDS_ID** | **Annotation** | **Contig** | **Start_bp** | **End_bp** | **Strand** |
| --- | --- | --- | --- | --- | --- | --- |
| R2 | JDPUOZLI_CDS_0001 | RusA-like Holliday junction resolvase | edge_1 | 3343826 | 3344239 | + |
| R2 | JDPUOZLI_CDS_0006 | Rz-like spanin | edge_1 | 3346360 | 3346767 | + |
| R2 | JDPUOZLI_CDS_0010 | terminase small subunit | edge_1 | 3347880 | 3348383 | + |
| R2 | JDPUOZLI_CDS_0011 | terminase large subunit | edge_1 | 3348358 | 3350445 | + |
| R2 | JDPUOZLI_CDS_0012 | head-tail adaptor Ad1 | edge_1 | 3350447 | 3350662 | + |
| R2 | JDPUOZLI_CDS_0013 | portal protein | edge_1 | 3350655 | 3352136 | + |
| R2 | JDPUOZLI_CDS_0014 | head maturation protease | edge_1 | 3352120 | 3353541 | + |
| R2 | JDPUOZLI_CDS_0016 | major head protein | edge_1 | 3354307 | 3355311 | + |
| R2 | JDPUOZLI_CDS_0017 | minor tail protein | edge_1 | 3355355 | 3356116 | + |
| R2 | JDPUOZLI_CDS_0020 | major tail protein with Ig-like domain | edge_1 | 3356915 | 3357667 | + |
| R2 | JDPUOZLI_CDS_0021 | tail assembly chaperone | edge_1 | 3357670 | 3358089 | + |
| R2 | JDPUOZLI_CDS_0023 | tail length tape measure protein | edge_1 | 3358306 | 3361023 | + |
| R2 | JDPUOZLI_CDS_0024 | virion structural protein | edge_1 | 3361053 | 3361379 | + |
| R2 | JDPUOZLI_CDS_0025 | minor tail protein | edge_1 | 3361376 | 3361837 | + |
| R2 | JDPUOZLI_CDS_0026 | minor tail protein | edge_1 | 3361837 | 3362229 | + |
| R2 | JDPUOZLI_CDS_0027 | central tail fiber J | edge_1 | 3362220 | 3370334 | + |
| R2 | JDPUOZLI_CDS_0004 | endolysin | edge_1 | 3345513 | 3346001 | + |

**Supplementary Table S43. Key prophage hallmark genes annotated in Region R3 (edge_1:4,088,887–4,099,432).** Pharokka-derived annotation of the core replication/head module in R3, including a replication initiation protein, single-stranded DNA-binding protein, and head morphogenesis/packaging markers (minor head protein and head morphogenesis). Coordinates are reported as genome positions on contig edge_1 with strand orientation.

| **Prophage_region** | **CDS_ID** | **Annotation** | **Contig** | **Start_bp** | **End_bp** | **Strand** |
| --- | --- | --- | --- | --- | --- | --- |
| R3 | CECNNIOU_CDS_0004 | replication initiation protein | edge_1 | 4089514 | 4090617 | + |
| R3 | CECNNIOU_CDS_0005 | single strand DNA binding protein | edge_1 | 4090599 | 4090877 | + |
| R3 | CECNNIOU_CDS_0010 | minor head protein | edge_1 | 4092558 | 4092905 | + |
| R3 | CECNNIOU_CDS_0011 | head morphogenesis | edge_1 | 4092907 | 4094226 | + |

**Supplementary Table S44. Gene content and functional features of the GI-4–associated integrative and mobilizable element (IME) detected by ICEberg 3.0.** Locus tags, genomic coordinates (with strand), gene lengths, predicted products, and functional feature calls for the IME inserted near tRNA-Ser (edge_1:533,895–553,050; 19,156 bp; 57.9% GC). Core mobilization and integration functions include a MOBQ-family relaxase (NG_SM01_00466) and phage-type integrases (NG_SM01_00473 and NG_SM01_00483), while regulatory cargo candidates include YejK (NG_SM01_00475) and CsrA (NG_SM01_00485). Flanking genes are listed to indicate element boundaries and local genomic context.

| **Locus tag** | **Genomic coordinates (strand)** | **Length (bp)** | **Predicted product** | **Functional feature** | |
| --- | --- | --- | --- | --- | --- |
| NG_SM01_00461 | 525686–526856 (+) | 1171 | Inner membrane protein YbjJ | | Flank |
| NG_SM01_00462 | 526863–528009 (−) | 1147 | Hypothetical protein | | Flank |
| NG_SM01_00463 | 528234–530613 (+) | 2380 | Vitamin B12 transporter outer membrane protein BtuB | | Flank |
| NG_SM01_00464 | 530621–532730 (+) | 2110 | Non-hemolytic phospholipase C | | Flank |
| NG_SM01_00465 | 532796–533717 (−) | 922 | Hypothetical protein | | Flank (GI boundary) |
| NG_SM01_00466 | 534675–536259 (−) | 1585 | MOBQ family relaxase | | Mobility (relaxase) |
| NG_SM01_00467 | 536521–536776 (+) | 256 | Hypothetical protein | | GI cargo (unknown) |
| NG_SM01_00468 | 536869–537052 (−) | 184 | Hypothetical protein | | GI cargo (unknown) |
| NG_SM01_00469 | 537117–538287 (−) | 1171 | Hypothetical protein | | GI cargo (unknown) |
| NG_SM01_00470 | 538561–538903 (+) | 343 | Hypothetical protein | | GI cargo (unknown) |
| NG_SM01_00471 | 539704–540838 (−) | 1135 | Hypothetical protein | | GI cargo (unknown) |
| NG_SM01_00472 | 540959–542654 (−) | 1696 | Hypothetical protein | | GI cargo (unknown) |
| NG_SM01_00473 | 542999–544400 (+) | 1402 | Phage integrase | | Integrase |
| NG_SM01_00474 | 544475–545738 (−) | 1264 | Hypothetical protein | | GI cargo (unknown) |
| NG_SM01_00475 | 545747–546563 (−) | 817 | Nucleoid-associated protein YejK | | GI cargo (stress/adaptation marker) |
| NG_SM01_00476 | 547021–547255 (−) | 235 | Hypothetical protein | | GI cargo (unknown) |
| NG_SM01_00477 | 547465–548233 (+) | 769 | Hypothetical protein | | GI cargo (unknown) |
| NG_SM01_00478 | 548263–548584 (+) | 322 | Hypothetical protein | | GI cargo (unknown) |
| NG_SM01_00479 | 548591–549053 (+) | 463 | Hypothetical protein | | GI cargo (unknown) |
| NG_SM01_00480 | 549420–549657 (+) | 238 | Hypothetical protein | | GI cargo (unknown) |
| NG_SM01_00481 | 549659–550604 (−) | 946 | Hypothetical protein | | GI cargo (unknown) |
| NG_SM01_00482 | 550844–551288 (−) | 445 | Hypothetical protein | | GI cargo (unknown) |
| NG_SM01_00483 | 551472–552951 (−) | 1480 | Phage integrase | | Integrase |
| NG_SM01_00484 | 553033–553126 (−) | 94 | tRNA-Ser | | tRNA (GI hotspot) |
| NG_SM01_00485 | 553203–553407 (−) | 205 | Translational regulator CsrA (csrA) | | Flank; virulence-associated factor |
| NG_SM01_00486 | 553554–556203 (−) | 2650 | Alanine—tRNA ligase (AlaRS) | | Flank |
| NG_SM01_00487 | 556312–556807 (−) | 496 | Regulatory protein RecX | | Flank |
| NG_SM01_00488 | 556917–558018 (−) | 1102 | Recombinase RecA | | Flank |
| NG_SM01_00489 | 558159–558795 (−) | 637 | LexA repressor | | Flank |

**Supplementary Table S45. ISfinder hits detected in the NG-SM01 genome and length-based classification.** Table lists all ISfinder significant alignments, including IS element name, IS family, contig, genomic coordinates (start/end), bitscore, E-value, and strand. An additional column reports the alignment length (hit_len_bp) and a practical length-based class (MICROTRACE, TRACE, FRAGMENT) to distinguish short motif-level matches from longer fragments. These hits were further cross-compared against GI_3/GI_4/GI_6 coordinates and ±200 bp boundary windows, and against the three prophage regions, to assess physical linkage between IS-family signatures and horizontally acquired regions.

| **IS_element** | **IS_family** | **Contig** | **Start_bp** | **End_bp** | **Bitscore** | **E_value** | **Strand** | **hit_len_bp** | **class_by_len** |
| --- | --- | --- | --- | --- | --- | --- | --- | --- | --- |
| ISRe46 | IS481 | edge_1 | 387600 | 387633 | 52 | 0.008 | Plus/Minus | 34 | MICROTRACE |
| TnXo19 | Tn3 | edge_1 | 403004 | 403025 | 44.1 | 1.9 | Plus/Minus | 22 | MICROTRACE |
| ISStma7 | IS110 | edge_1 | 1157177 | 1157201 | 50.1 | 0.031 | Plus/Plus | 25 | MICROTRACE |
| ISStma8 | IS110 | edge_1 | 1157177 | 1157201 | 50.1 | 0.031 | Plus/Plus | 25 | MICROTRACE |
| ISArsp14 | ISNCY | edge_1 | 1747972 | 1748012 | 58 | 1.00E-04 | Plus/Plus | 41 | MICROTRACE |
| ISAzs17 | Tn3 | edge_1 | 1834598 | 1834936 | 204 | 9.00E-49 | Plus/Plus | 339 | FRAGMENT |
| ISAzs17 | Tn3 | edge_1 | 1835089 | 1835121 | 42.1 | 7.6 | Plus/Plus | 33 | MICROTRACE |
| ISAzs17 | Tn3 | edge_1 | 1835611 | 1835650 | 48.1 | 0.12 | Plus/Minus | 40 | MICROTRACE |
| ISAzs17 | Tn3 | edge_1 | 1836120 | 1836169 | 44.1 | 1.9 | Plus/Minus | 50 | TRACE |
| ISAzs17 | Tn3 | edge_1 | 1836226 | 1836402 | 129 | 4.00E-26 | Plus/Minus | 177 | TRACE |
| ISAzs17 | Tn3 | edge_1 | 1836466 | 1836665 | 87.7 | 1.00E-13 | Plus/Minus | 200 | FRAGMENT |
| ISAzs17 | Tn3 | edge_1 | 1836805 | 1837094 | 83.8 | 2.00E-12 | Plus/Minus | 290 | FRAGMENT |
| TnXo19 | Tn3 | edge_1 | 2108710 | 2108758 | 42.1 | 7.6 | Plus/Minus | 49 | MICROTRACE |
| TnXo19 | Tn3 | edge_1 | 2110316 | 2110372 | 73.8 | 2.00E-09 | Plus/Minus | 57 | TRACE |
| TnXo19 | Tn3 | edge_1 | 2111147 | 2111181 | 54 | 0.002 | Plus/Minus | 35 | MICROTRACE |
| TnXo19 | Tn3 | edge_1 | 2111333 | 2111385 | 65.9 | 5.00E-07 | Plus/Minus | 53 | TRACE |
| ISStma7 | IS110 | edge_1 | 2114593 | 2114616 | 48.1 | 0.12 | Plus/Plus | 24 | MICROTRACE |
| ISStma8 | IS110 | edge_1 | 2114593 | 2114616 | 48.1 | 0.12 | Plus/Plus | 24 | MICROTRACE |
| ISArsp14 | ISNCY | edge_1 | 2399425 | 2399450 | 44.1 | 1.9 | Plus/Plus | 26 | MICROTRACE |
| ISArsp14 | ISNCY | edge_1 | 2583542 | 2583572 | 46.1 | 0.48 | Plus/Plus | 31 | MICROTRACE |
| ISStma7 | IS110 | edge_1 | 2611005 | 2611030 | 52 | 0.008 | Plus/Minus | 26 | MICROTRACE |
| ISStma8 | IS110 | edge_1 | 2611005 | 2611030 | 52 | 0.008 | Plus/Minus | 26 | MICROTRACE |
| ISStma7 | IS110 | edge_1 | 2785696 | 2785720 | 50.1 | 0.031 | Plus/Minus | 25 | MICROTRACE |
| ISStma8 | IS110 | edge_1 | 2785696 | 2785720 | 50.1 | 0.031 | Plus/Minus | 25 | MICROTRACE |
| ISPa40 | Tn3 | edge_1 | 2893772 | 2893798 | 54 | 0.002 | Plus/Minus | 27 | MICROTRACE |
| ISStma7 | IS110 | edge_1 | 2905317 | 2905339 | 46.1 | 0.48 | Plus/Plus | 23 | MICROTRACE |
| ISStma8 | IS110 | edge_1 | 2905317 | 2905339 | 46.1 | 0.48 | Plus/Plus | 23 | MICROTRACE |
| ISStma7 | IS110 | edge_1 | 2976232 | 2976256 | 50.1 | 0.031 | Plus/Plus | 25 | MICROTRACE |
| ISStma8 | IS110 | edge_1 | 2976232 | 2976256 | 50.1 | 0.031 | Plus/Plus | 25 | MICROTRACE |
| ISVsp2 | IS1634 | edge_1 | 3055471 | 3055497 | 46.1 | 0.48 | Plus/Plus | 27 | MICROTRACE |
| ISVsp2 | IS1634 | edge_1 | 3055477 | 3055503 | 54 | 0.002 | Plus/Plus | 27 | MICROTRACE |
| ISVsp2 | IS1634 | edge_1 | 3055481 | 3055504 | 48.1 | 0.12 | Plus/Plus | 24 | MICROTRACE |
| ISAzs17 | Tn3 | edge_1 | 3673743 | 3673775 | 42.1 | 7.6 | Plus/Minus | 33 | MICROTRACE |
|  |  |  |  |  |  |  |  |  |  |

**Supplementary Table S46. Resistance and virulence determinants detected in NG-SM01 across multiple annotation sources.**Genomic coordinates (start, end, strand) and gene names for antimicrobial resistance and virulence-associated loci detected in NG-SM01, with source support (ResFinder, CARD, AMRFinderPlus, VFDB/Prokka) and associated identity/coverage (or source-specific information). This table includes aminoglycoside resistance determinants (aph(3′)-IIc, aac(6′)-Iz), intrinsic β-lactamases (blaL1 and blaL2-like), and efflux systems (SmeDEF and EmrABC), as well as the single VFDB adherence-associated hit PilG.

| **Category** | **Start** | **End** | **Strand** | **Gene** | **Source support** | **%ID (or info)** | **%Cov (or info)** |
| --- | --- | --- | --- | --- | --- | --- | --- |
| resistance | 62698 | 63510 | + | aph(3')-IIc_1 | ResFinder | ResFinder:84.77 | ResFinder:99.88 |
| resistance | 741268 | 742851 | − | emrB | AMRFinder | AMRFinder:96.59 | AMRFinder:100.00 |
| resistance | 742862 | 744037 | − | emrA | AMRFinder | AMRFinder:96.44 | AMRFinder:100.00 |
| resistance | 744052 | 745542 | − | emrC | AMRFinder | AMRFinder:92.76 | AMRFinder:100.00 |
| resistance | 2597970 | 2599151 | + | smeD | CARD | CARD:91.73 | CARD:99.75 |
| resistance | 2599164 | 2602273 | + | smeE | CARD | CARD:93.18 | CARD:99.58 |
| resistance | 2602375 | 2603760 | + | smeF | AMRFinder | AMRFinder:90.69 | AMRFinder:99.14 |
| resistance | 2602405 | 2603760 | + | smeF | CARD | CARD:87.68 | CARD:96.79 |
| resistance | 2964480 | 2965391 | + | blaL2_like (NG_SM01_02683) | BLASTp (L2_uniprot_P96465)+Prokka | BLASTp:80.86 | BLASTp:100.00 |
| resistance | 3083282 | 3083746 | + | aac(6') | AMRFinder | AMRFinder:98.71 | AMRFinder:100.00 |
| resistance | 3083282 | 3083730 | + | aac(6')-Iz | CARD | CARD:84.63 | CARD:97.19 |
| resistance | 3083282 | 3083730 | + | aac(6')-Iz_1 | ResFinder | ResFinder:84.63 | ResFinder:97.19 |
| resistance | 3990346 | 3991128 | − | blaL1_3 | ResFinder | ResFinder:82.40 | ResFinder:89.89 |
| virulence_screen | 3017819 | 3018154 | + | phoP_4 | VFDB; Prokka | 80.71 | 82.11 |

**Supplementary Table S47. Comparative BLASTp profiling of the blaL1 protein across NG-SM01 and 19 reference proteomes.**BLASTp summary statistics for the NG-SM01 blaL1 protein (261 aa) queried against 19 reference proteomes, reporting best percent identity, alignment length, query and subject coverage, E-value, and bitscore. The table highlights allele clustering with a single 100% identical match (GCF_025642255.1) and a second cluster of divergent but full-length matches (~80.84–83.14% identity; 100% query coverage) across the remaining references.

| **Ref ID** | **Best % identity** | **Aln length** | **Qcov %** | **Scov %** | **E-value** | **Bitscore** |
| --- | --- | --- | --- | --- | --- | --- |
| GCF_025642255.1 | 100 | 261 | 100 | 84.47 | 0 | 535 |
| GCF_002138415.1 | 80.843 | 261 | 100 | 90 | 7.27E-164 | 452 |
| GCF_001274595.1 | 81.226 | 261 | 100 | 83.92 | 4.07E-155 | 431 |
| GCF_004346925.1 | 80.843 | 261 | 100 | 90 | 1.01E-163 | 452 |
| GCF_003205835.1 | 82.375 | 261 | 100 | 90 | 2.09E-159 | 441 |
| GCF_001431665.1 | 82.375 | 261 | 100 | 83.92 | 1.16E-164 | 455 |
| GCF_006970445.1 | 81.992 | 261 | 100 | 90 | 5.47E-166 | 458 |
| GCF_900186865.1 | 82.759 | 261 | 100 | 85.02 | 4.94E-165 | 456 |
| GCF_006974125.1 | 81.609 | 261 | 100 | 90 | 2.02E-156 | 434 |
| GCF_002799165.1 | 81.609 | 261 | 100 | 90 | 4.07E-163 | 451 |
| GCF_002799155.1 | 81.992 | 261 | 100 | 83.92 | 7.81E-159 | 441 |
| GCF_002799245.1 | 81.226 | 261 | 100 | 90 | 4.87E-164 | 453 |
| GCF_000020665.1 | 83.142 | 261 | 100 | 90 | 8.09E-168 | 462 |
| GCF_013464915.1 | 83.142 | 261 | 100 | 90 | 2.51E-160 | 443 |
| GCF_013004645.1 | 81.609 | 261 | 100 | 90 | 4.23E-164 | 453 |
| GCF_001676385.1 | 81.609 | 261 | 100 | 90 | 2.01E-160 | 444 |
| GCF_004920835.1 | 81.992 | 261 | 100 | 90 | 1.69E-159 | 441 |
| GCF_004684085.1 | 81.992 | 261 | 100 | 90 | 1.62E-158 | 439 |
| NBRC_14161 | 82.759 | 261 | 100 | 85.02 | 4.88E-165 | 456 |

**Supplementary Table S48. BacMet2_EXP matches for NG-SM01 panel-restricted genes.** Top four BacMet-supported hits detected among the 27 panel-restricted annotated genes, reporting percent identity, query/subject coverage, E-value, bitscore, and BacMet functional annotation. Tier 1 corresponds to high-confidence support based on coverage and score; the fourth hit (NG_SM01_03660; PmrB/SilS-like sensor kinase) is retained as Tier 2 due to reduced query coverage (~51%).

| **LocusTag** | **BacMet hit  (gene; acc; org)** | **%ID** | **Qcov** | **Scov** | **E-value** | **Bits** | **Function (from BacMet)** |
| --- | --- | --- | --- | --- | --- | --- | --- |
| NG_SM01_01595 | yfeB; Q56953; Y. pestis | 24.4 | 92.9 | 74.7 | 3.36E-22 | 87.8 | YfeB chelated-iron transport membrane protein |
| NG_SM01_03660 | pmrB; Q70FG9; Pectobacterium sp. | 29.6 | 51 | 63.2 | 1.13E-19 | 85.1 | PmrB sensor histidine kinase (two-component system) |
| NG_SM01_03661 | czcR; Q44006; R. metallidurans | 38.5 | 98.7 | 98.2 | 3.55E-41 | 135 | CzcR transcriptional activator (metal response regulator) |
| NG_SM01_03684 | adeL; A3M732; A. baumannii | 32.9 | 95.4 | 85.8 | 2.14E-35 | 125 | LysR-family transcriptional regulator (AdeL-like) |

**Supplementary Table S49. High-confidence (Tier 1) BacMet-supported adaptome anchors within the NG-SM01 panel-restricted -gene set.** Subset of the strongest three BacMet hits (CzcR-like regulator, AdeL-like LysR regulator, and YfeB-like metal/Fe transport component) summarized with abbreviated functional descriptions for rapid cross-reference with Fig. 7.

| **LocusTag** | **BacMet hit (gene; acc; org)** | **%ID** | **Qcov** | **Scov** | **E-value** | **Bits** | **Function (short)** |
| --- | --- | --- | --- | --- | --- | --- | --- |
| NG_SM01_01595 | yfeB; Q56953; Y. pestis | 24.4 | 92.9 | 74.7 | 3.36E-22 | 87.8 | YfeB chelated-Fe transport (membrane) |
| NG_SM01_03661 | czcR; Q44006; R. metallidurans | 38.5 | 98.7 | 98.2 | 3.55E-41 | 135 | CzcR metal-response regulator (TF) |
| NG_SM01_03684 | adeL; A3M732; A. baumannii | 32.9 | 95.4 | 85.8 | 2.14E-35 | 125 | AdeL-like LysR regulator (TF) |

**Supplementary Table S50. gapseq reconstruction output statistics for NG-SM01.** Summary metrics from the gapseq workflow, including pathway and transporter entry totals, reaction table size, number of model reactions mapped, gene support statistics, and evidence-tier counts for reaction weights (rxnWeights), corresponding to Fig. 8A–D.

| **Metric** | **Value** |
| --- | --- |
| Pathways_total | 3159 |
| Pathways_TRUE | 380 |
| Pathways_FALSE | 2776 |
| Reactions_total_entries | 38366 |
| Transporter_table_lines | 1920 |
| Model_reactions_mapped | 13134 |
| Model_reactions_gene_supported | 13134 |
| Model_reactions_no_gene | 0 |
| Pct_mapped_gene_supported | 100 |
| rxnWeights_total | 7543 |
| rxnWeights_minWeight_0.005 | 5103 |
| rxnWeights_pct_minWeight | 67.64 |
| rxnWeights_equal_100 | 1515 |
| rxnWeights_pct_equal_100 | 20.08 |
| rxnWeights_ge_1 | 2440 |
| rxnWeights_pct_ge_1 | 32.35 |
|  |  |

**Supplementary Table S51. Summary of transporter functional categories predicted by gapseq.** Counts and percentages of transporter entries classified into major substrate-related categories (metal-related, sugar-related, amino acid/peptide-related, organic acid-related, and inorganic-related). Percentages are reported relative to the categorized transporter subset used for summarized reporting.

| **Category** | **Count** | **Percent (%)** |
| --- | --- | --- |
| Metal_related | 476 | 42.73 |
| Sugar_related | 309 | 27.74 |
| Amino_peptide_related | 160 | 14.36 |
| Organic_acid_related | 71 | 6.37 |
| Inorganic_related | 98 | 8.8 |
|  |  |  |

**Supplementary Table S52. Predicted minimal medium composition inferred by gapseq for NG-SM01.** List of compounds required for the inferred minimal supportive medium, reported as ModelSEED compound identifiers with compound names and the corresponding maxFlux uptake constraints used in model evaluation. This table provides the full medium definition underlying Fig. 8C.

| **Compounds** | **Name** | **MaxFlux** |
| --- | --- | --- |
| cpd00001 | H2O | 100 |
| cpd00971 | Sodium | 10 |
| cpd00205 | K+ | 10 |
| cpd00099 | Cl- | 10 |
| cpd10515 | Fe2+ | 10 |
| cpd10516 | Fe3+ | 0.1 |
| cpd00009 | Phosphate | 10 |
| cpd00149 | Cobalt | 10 |
| cpd00254 | Mg | 10 |
| cpd00244 | Nickel | 1 |
| cpd00048 | Sulfate | 10 |
| cpd00239 | H2S | 1 |
| cpd00034 | Zn2+ | 10 |
| cpd00058 | Cu2+ | 10 |
| cpd00030 | Mn2+ | 10 |
| cpd00063 | Ca2+ | 10 |
| cpd00013 | Ammonium | 10 |
| cpd00007 | O2 | 12.5 |
| cpd00027 | D-Glucose | 5 |
| cpd00082 | D-Fructose | 5 |
| cpd00794 | Trehalose | 2.5 |
| cpd00154 | Xylose | 5 |
| cpd00054 | L-Serine | 0.1 |
| cpd00322 | L-Isoleucine | 0.1 |
| cpd00220 | Riboflavin | 0.025 |
| cpd00067 | H+ | 0.025 |

**Supplementary Table S53. Crystal violet microtiter biofilm assay—blank-corrected biomass and growth-normalized biofilm index (n = 5).** Biofilm formation was quantified in 96-well plates by crystal violet staining with absorbance measured at 595 nm. Planktonic growth was recorded at 600 nm prior to washing. For each strain (*E. coli* ATCC 25922, *K. pneumoniae* ATCC 13883, *P. aeruginosa* ATCC 27853, and NG-SM01), values are shown for five independent replicates. Blank correction was performed using the corresponding negative-control (medium-only) wells: OD600corr = OD600_sample − OD600_blank and OD595corr = OD595_sample − OD595_blank. The normalized biofilm index was calculated to adjust for growth differences as OD595corr/OD600corr.

| **Strain** | **Rep** | **OD600corr** | **OD595corr (Biofilm biomass)** | **Normalized biofilm (OD595corr/OD600corr)** |
| --- | --- | --- | --- | --- |
| *E.coli* | 1 | 1.0367 | 0.0015 | 0.0014 |
| *E.coli* | 2 | 1.0217 | 0.0144 | 0.0141 |
| *E.coli* | 3 | 1.2742 | 0.012 | 0.0094 |
| *E.coli* | 4 | 1.3057 | 0.0101 | 0.0077 |
| *E.coli* | 5 | 1.0597 | 0.0626 | 0.0591 |
| *Klebsiella* | 1 | 1.1238 | 0.0707 | 0.0629 |
| *Klebsiella* | 2 | 1.0948 | 0.0578 | 0.0528 |
| *Klebsiella* | 3 | 1.1037 | 0.0532 | 0.0482 |
| *Klebsiella* | 4 | 1.3098 | 0.0487 | 0.0372 |
| *Klebsiella* | 5 | 1.1263 | 0.1343 | 0.119 |
| *Pseudomonas* | 1 | 1.3094 | 0.109 | 0.0832 |
| *Pseudomonas* | 2 | 1.2534 | 0.2242 | 0.1789 |
| *Pseudomonas* | 3 | 1.2982 | 0.1447 | 0.1115 |
| *Pseudomonas* | 4 | 1.517 | 0.0404 | 0.0266 |
| *Pseudomonas* | 5 | 1.2823 | 0.2654 | 0.207 |
| NG-SM01 | 1 | 1.2404 | 0.0926 | 0.0747 |
| NG-SM01 | 2 | 0.9708 | 0.0792 | 0.0816 |
| NG-SM01 | 3 | 0.9509 | 0.0986 | 0.1037 |
| NG-SM01 | 4 | 0.9577 | 0.1006 | 0.105 |
| NG-SM01 | 5 | 1.012 | 0.176 | 0.1739 |

**Supplementary Table S54. Summary of crystal violet biofilm formation for NG-SM01 and reference strains (mean ± SD, n = 5).** Biofilm biomass is reported as blank-corrected OD595 (OD595corr) after crystal violet staining, where OD595corr = OD595_sample − OD595_blank (negative-control wells). A growth-normalized biofilm index is additionally shown as OD595corr/OD600corr, with OD600corr = OD600_sample − OD600_blank measured prior to washing. Values represent the mean ± SD of five independent replicates for *E. coli* ATCC 25922, *K. pneumoniae* ATCC 13883, *P. aeruginosa* ATCC 27853, and NG-SM01.

| **Strain** | **OD595corr (Biofilm biomass) mean ± SD** | **Normalized biofilm mean ± SD** |
| --- | --- | --- |
| *E.coli* | 0.0201 ± 0.0242 | 0.0184 ± 0.0232 |
| *Klebsiella* | 0.0729 ± 0.0353 | 0.0641 ± 0.0322 |
| *Pseudomonas* | 0.1567 ± 0.0899 | 0.1214 ± 0.0727 |
| NG-SM01 | 0.1094 ± 0.0382 | 0.1078 ± 0.0393 |

**Supplementary Table S55. Pairwise statistical comparison of NG-SM01 biofilm biomass versus reference strains (Welch’s t-test with Holm correction).** Biofilm biomass was quantified as blank-corrected OD595 (OD595corr) from the crystal violet assay (n = 5 per strain). NG-SM01 was compared against each reference strain (*E. coli* ATCC 25922, *K. pneumoniae* ATCC 13883, *P. aeruginosa* ATCC 27853) using Welch’s two-sided t-test. To control for multiple testing across the three comparisons, p-values were adjusted using the Holm method. Effect sizes are reported as Hedges’ g, and mean differences are expressed as (NG-SM01 − reference).

| **Comparison** | **NG-SM01 mean ± SD** | **Reference mean ± SD** | **Mean difference (NG − Ref)** | **Welch p** | **Holm-adj p** | **Hedges’ g** | **Interpretation** |
| --- | --- | --- | --- | --- | --- | --- | --- |
| NG-SM01 vs *E.coli* | 0.1094 ± 0.0382 | 0.0201 ± 0.0242 | 0.0893 | 0.00335 | 0.01 | 2.52 | Significant; NG-SM01 higher |
| NG-SM01 vs *Klebsiella* | 0.1094 ± 0.0382 | 0.0729 ± 0.0353 | 0.0365 | 0.156 | 0.311 | 0.9 | Not significant |
| NG-SM01 vs *Pseudomonas* | 0.1094 ± 0.0382 | 0.1567 ± 0.0899 | −0.0473 | 0.324 | 0.324 | −0.62 | Not significant |

**Supplementary Table S56. Pairwise statistical comparison of NG-SM01 versus reference strains using the growth-normalized biofilm index (Welch’s t-test with Holm correction).** Biofilm formation was evaluated by the **growth-normalized biofilm index** calculated as **OD595corr/OD600corr** from the crystal violet assay (n = 5 per strain), where OD595corr and OD600corr are blank-corrected values relative to negative-control wells. NG-SM01 was compared against each reference strain (*E. coli* ATCC 25922, *K. pneumoniae* ATCC 13883, *P. aeruginosa* ATCC 27853) using Welch’s two-sided t-test. P-values were adjusted for multiple comparisons across the three pairwise tests using the Holm method. Effect sizes are reported as Hedges’ g, and mean differences are expressed as (NG-SM01 − reference).

| **Comparison** | **NG-SM01 mean ± SD** | **Reference mean ± SD** | **Mean difference (NG − Ref)** | **Welch p** | **Holm-adj p** | **Hedges’ g** | **Interpretation** |
| --- | --- | --- | --- | --- | --- | --- | --- |
| NG-SM01 vs *E.coli* | 0.1078 ± 0.0393 | 0.0184 ± 0.0232 | 0.0894 | 0.00388 | 0.0116 | 2.5 | Significant; NG-SM01 higher |
| NG-SM01 vs *Klebsiella* | 0.1078 ± 0.0393 | 0.0641 ± 0.0322 | 0.0437 | 0.092 | 0.184 | 1.1 | Not significant (trend) |
| NG-SM01 vs *Pseudomonas* | 0.1078 ± 0.0393 | 0.1214 ± 0.0727 | −0.0137 | 0.724 | 0.724 | −0.21 | Not significant |

**Supplementary Table S57. Absolute survival after acute H₂O₂ exposure (log₁₀ CFU/mL).** Mid-log cultures (OD₆₀₀ = 0.5) of *P. aeruginosa* ATCC 27853, *K.pneumoniae* ATCC 13883, *Stenotrophomonas* sp. NG-SM01, and *E.coli* ATCC 25922 were exposed to H₂O₂ (0–3.0 mM) for 5 min, neutralized by stop dilution, serially diluted, and recovery-plated on LB agar. Values are reported as log₁₀(CFU/mL) back-calculated to the OD-standardized starting suspension and summarized as mean ± SD from three biological replicates (n = 3) per dose. Colony counts were taken from the dilution yielding 10–30 CFU per 10-µL spot; confluent growth (“g”) was excluded from CFU estimation.

| **H₂O₂ (mM)** | ***P.aeruginosa* ATCC 27853** | ***K. pneumoniae* ATCC 13883** | **NG-SM01** | ***E.coli* ATCC 25922** |
| --- | --- | --- | --- | --- |
| 0 | 9.451 ± 0.032 | 8.371 ± 0.061 | 8.348 ± 0.041 | 8.452 ± 0.023 |
| 0.25 | 9.436 ± 0.034 | 7.364 ± 0.074 | 7.315 ± 0.024 | 7.415 ± 0.017 |
| 0.5 | 9.436 ± 0.034 | 7.330 ± 0.088 | 7.244 ± 0.063 | 7.377 ± 0.061 |
| 0.75 | 9.420 ± 0.019 | 7.315 ± 0.012 | 7.234 ± 0.078 | 7.409 ± 0.010 |
| 1 | 8.346 ± 0.059 | 7.247 ± 0.014 | 7.180 ± 0.091 | 7.348 ± 0.030 |
| 1.5 | 8.313 ± 0.059 | 7.155 ± 0.046 | 7.164 ± 0.060 | 7.230 ± 0.026 |
| 2 | 8.262 ± 0.036 | 7.145 ± 0.031 | 7.221 ± 0.040 | 7.195 ± 0.016 |
| 2.5 | 7.308 ± 0.012 | 7.191 ± 0.072 | 7.172 ± 0.074 | 7.156 ± 0.017 |
| 3 | 7.338 ± 0.074 | 7.230 ± 0.026 | 7.213 ± 0.104 | 7.078 ± 0.037 |

**Supplementary Table S58. Dose–response trend analysis of oxidative killing across 1.0–3.0 mM H₂O₂.** For each strain (*P.aeruginosa* ATCC 27853, *K.pneumoniae* ATCC 13883, *Stenotrophomonas* sp. NG-SM01, and *E.coli* ATCC 25922), a linear regression was fitted to replicate-level log₁₀ reductions (relative to the matched 0 mM control) measured at 1.0, 1.5, 2.0, 2.5, and 3.0 mM H₂O₂ (5 doses × 3 biological replicates = 15 observations per strain). The table reports the slope (log₁₀ reduction per mM), R², and p value testing whether the slope differs from zero (dose dependence).

| **Strain** | **Slope (log₁₀ reduction per mM)** | **R²** | **p (slope)** |
| --- | --- | --- | --- |
| *P.aeruginosa* ATCC 27853 | 0.604 | 0.773 | 1.60×10⁻⁵ |
| *K.pneumoniae* ATCC 13883 | −0.00047 | 0.00002 | 0.988 |
| NG-SM01 | −0.0152 | 0.0135 | 0.68 |
| *E.coli* ATCC 25922 | 0.123 | 0.854 | 8.45×10⁻⁷ |

**Supplementary Table S59. Two-way ANOVA of oxidative killing across strains and H₂O₂ doses (log₁₀ reduction).** A two-way ANOVA was performed on replicate-level log₁₀ reductions in viable counts (relative to the matched 0 mM control) measured after 5 min exposure to H₂O₂ (0–3.0 mM) for *P. aeruginosa* ATCC 27853, *K. pneumoniae* ATCC 13883, *Stenotrophomonas* sp. NG-SM01, and *E. coli* ATCC 25922 (n = 3 biological replicates per strain × dose). The table reports degrees of freedom (df), sums of squares (SS), mean squares (MS), F statistics, and p values for the main effects (strain, dose) and the strain×dose interaction.

| **Source** | **df** | **Sum of squares (SS)** | **Mean square (MS)** | **F** | **p value** |
| --- | --- | --- | --- | --- | --- |
| Strain | 3 | 0.521871 | 0.173957 | 35.16697 | 4.16×10⁻¹⁴ |
| H₂O₂ dose | 8 | 19.204724 | 2.400591 | 485.30094 | 2.03×10⁻⁵⁹ |
| Strain×Dose | 24 | 10.719492 | 0.446645 | 90.2934 | 2.24×10⁻⁴⁴ |
| Residual (error) | 72 | 0.356155 | 0.004946 | — | — |

**Supplementary Table S60. Time-kill kinetics expressed as Δlog₁₀ reduction versus baseline before H₂O₂ addition.** Mid-log cultures were exposed to 2.0 mM H₂O₂ and sampled at 0, 5, 15, 30, and 60 min. Viable counts were quantified as CFU/mL and converted to log₁₀ units. The 0-min value represents the paired baseline sampled immediately before H₂O₂ addition. Δlog₁₀ reduction was calculated within each biological replicate as: Δlog₁₀ = log₁₀(CFU/mL at baseline before H₂O₂ addition) − log₁₀(CFU/mL at time t). Values are reported as mean ± SD from three independent biological replicates per strain.

| **Time (min)** | ***P.aeruginosa*  ATCC 27853** | ***K. pneumoniae*  ATCC 13883** | **NG-SM01** | ***E.coli*  ATCC 25922** |
| --- | --- | --- | --- | --- |
| **0** | 0.000 ± 0.000 | 0.000 ± 0.000 | 0.000 ± 0.000 | 0.000 ± 0.000 |
| **5** | 1.161 ± 0.015 | 1.197 ± 0.040 | 1.132 ± 0.039 | 1.196 ± 0.041 |
| **15** | 1.081 ± 0.032 | 0.274 ± 0.009 | 0.139 ± 0.036 | 0.238 ± 0.029 |
| **30** | 1.054 ± 0.033 | 0.308 ± 0.035 | 0.118 ± 0.050 | 0.353 ± 0.052 |
| **60** | 1.043 ± 0.040 | 0.395 ± 0.021 | 0.117 ± 0.042 | 0.375 ± 0.028 |

**Supplementary Table S61. Two-way ANOVA of time-kill Δlog₁₀ reductions (strain × time) in the H₂O₂ proxy assay.**
Two-way ANOVA was performed on Δlog₁₀ reduction vs 0 min values calculated within each biological replicate (Δlog₁₀ = log₁₀[CFU/mL at 0 min; 0 H₂O₂] − log₁₀[CFU/mL at time *t*]). The model included the fixed effects of strain, time, and their interaction (strain × time), using measurements at 5, 15, 30, and 60 min (n = 3 biological replicates per strain per timepoint). Effect sizes are reported as partial η².

| **Effect** | **df** | **SS** | **F** | **p** | **Partial η²** |
| --- | --- | --- | --- | --- | --- |
| Strain | 3 | 3.434763 | 901.069 | 5.66E-31 | 0.988 |
| Time | 3 | 4.59473 | 1205.373 | 5.65E-33 | 0.991 |
| Strain × Time | 9 | 1.195076 | 104.505 | 3.76E-21 | 0.967 |
| Residual | 32 | 0.04066 | — | — | — |

**Supplementary Table S62. Tukey HSD post-hoc comparisons of NG-SM01 versus reference strains across time in the H₂O₂ proxy time-kill assay.** Following the two-way ANOVA (Supplementary Table S61), Tukey’s honestly significant difference (HSD) test was applied to Δlog₁₀ reduction vs 0 min values to compare NG-SM01 against each reference strain at each timepoint (5, 15, 30, and 60 min; n = 3 biological replicates per strain per timepoint). Mean differences are reported as NG-SM01 − comparator, with 95% confidence intervals and Tukey-adjusted p-values (p-adj); “Sig.” indicates significance at α = 0.05.

| **Time (min)** | **Comparison** | **Mean diff** | **95% CI (lower, upper)** | p-adj | Sig. |
| --- | --- | --- | --- | --- | --- |
| 5 | NG-SM01 − *P.aeruginosa* ATCC 27853 | −0.039 | (−0.109, 0.031) | 7.502e−01 | No |
| 5 | NG-SM01 − *K.pneumoniae* ATCC 13883 | −0.065 | (−0.135, 0.005) | 1.811e−01 | No |
| 5 | NG-SM01 − *E.coli* | −0.064 | (−0.134, 0.006) | 1.955e−01 | No |
| 15 | NG-SM01 − *P.aeruginosa* ATCC 27853 | −0.942 | (−1.012, −0.872) | 2.052e−14 | Yes |
| 15 | NG-SM01 − *K.pneumoniae* ATCC 13883 | −0.135 | (−0.205, −0.065) | 1.800e−03 | Yes |
| 15 | NG-SM01 − *E.coli* | −0.099 | (−0.169, −0.029) | 1.150e−02 | Yes |
| 30 | NG-SM01 − *P.aeruginosa* ATCC 27853 | −0.936 | (−1.006, −0.866) | 2.052e−14 | Yes |
| 30 | NG-SM01 − *K.pneumoniae* ATCC 13883 | −0.190 | (−0.260, −0.120) | 3.100e−03 | Yes |
| 30 | NG-SM01 − *E.coli* | −0.235 | (−0.305, −0.165) | 8.000e−04 | Yes |
| 60 | NG-SM01 − *P.aeruginosa* ATCC 27853 | −0.926 | (−0.996, −0.856) | 2.052e−14 | Yes |
| 60 | NG-SM01 − *K.pneumoniae* ATCC 13883 | −0.278 | (−0.348, −0.208) | 5.400e−05 | Yes |
| 60 | NG-SM01 − *E.coli* | −0.258 | (−0.328, −0.188) | 1.000e−04 | Yes |


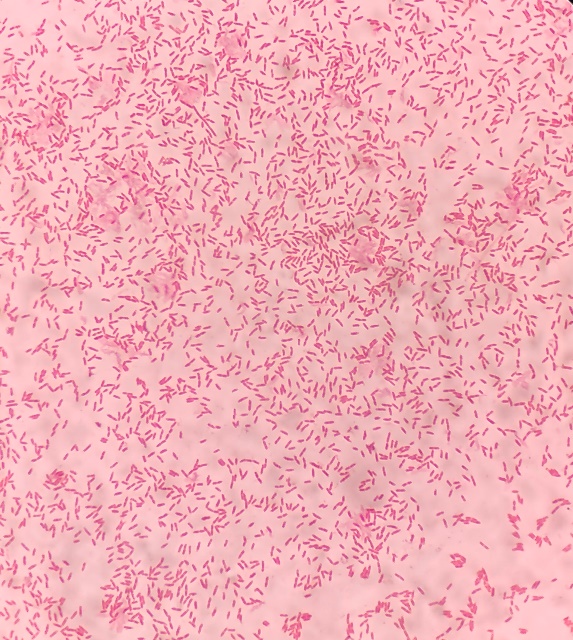


**Supplementary Fig. S1.** Gram-stained smear of the drinking-water isolate NG-SM01 showing abundant Gram-negative bacilli (pink, short rod-shaped cells), consistent with *Stenotrophomonas* morphology under light microscopy.


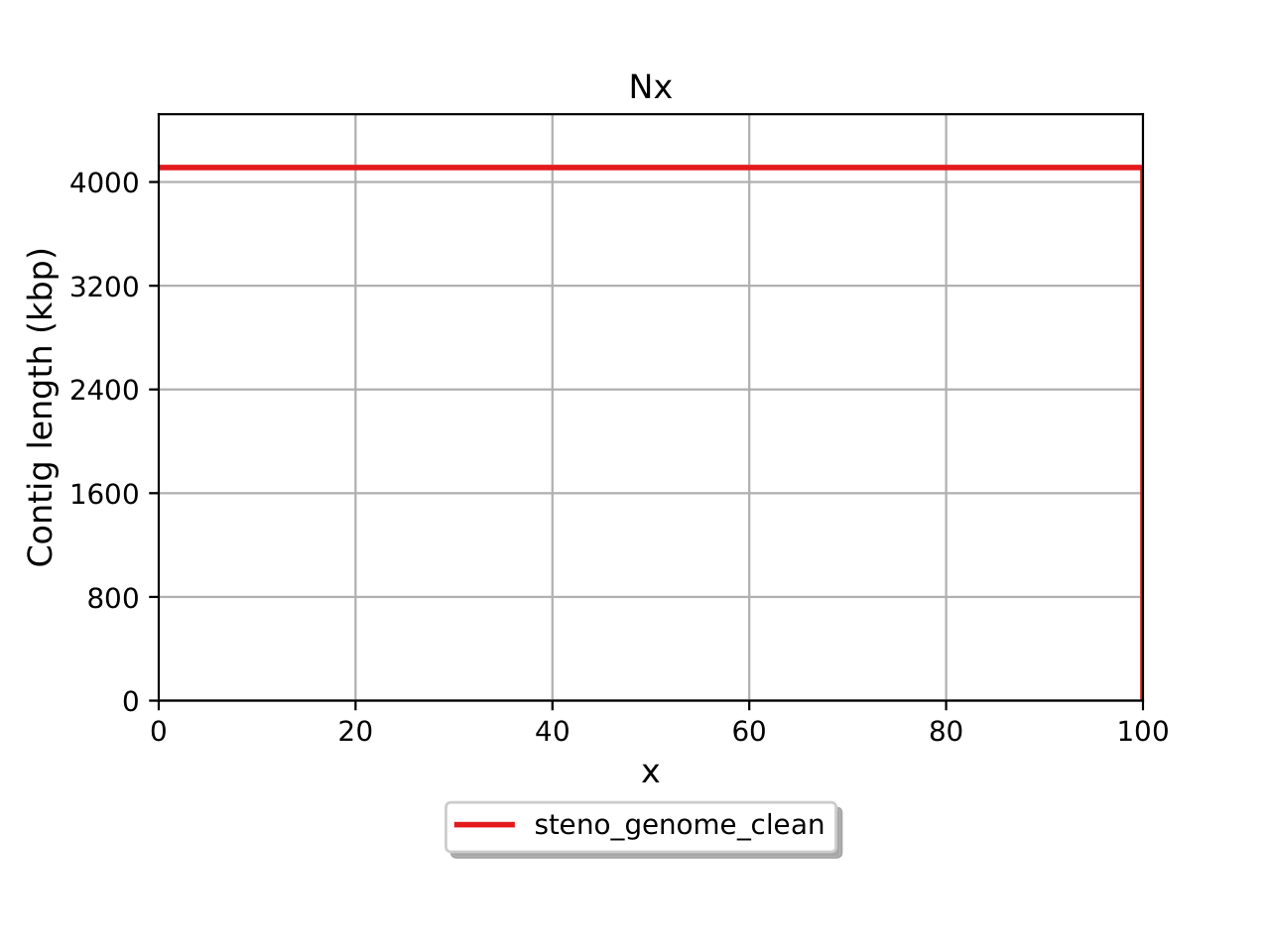


**Supplementary Fig. S2. Nx contiguity profile for the NG-SM01 assembly.** Nx plot showing contig length (kbp) as a function of x (0–100). The profile indicates a single chromosome-scale contig across Nx thresholds, consistent with a fully contiguous assembly.


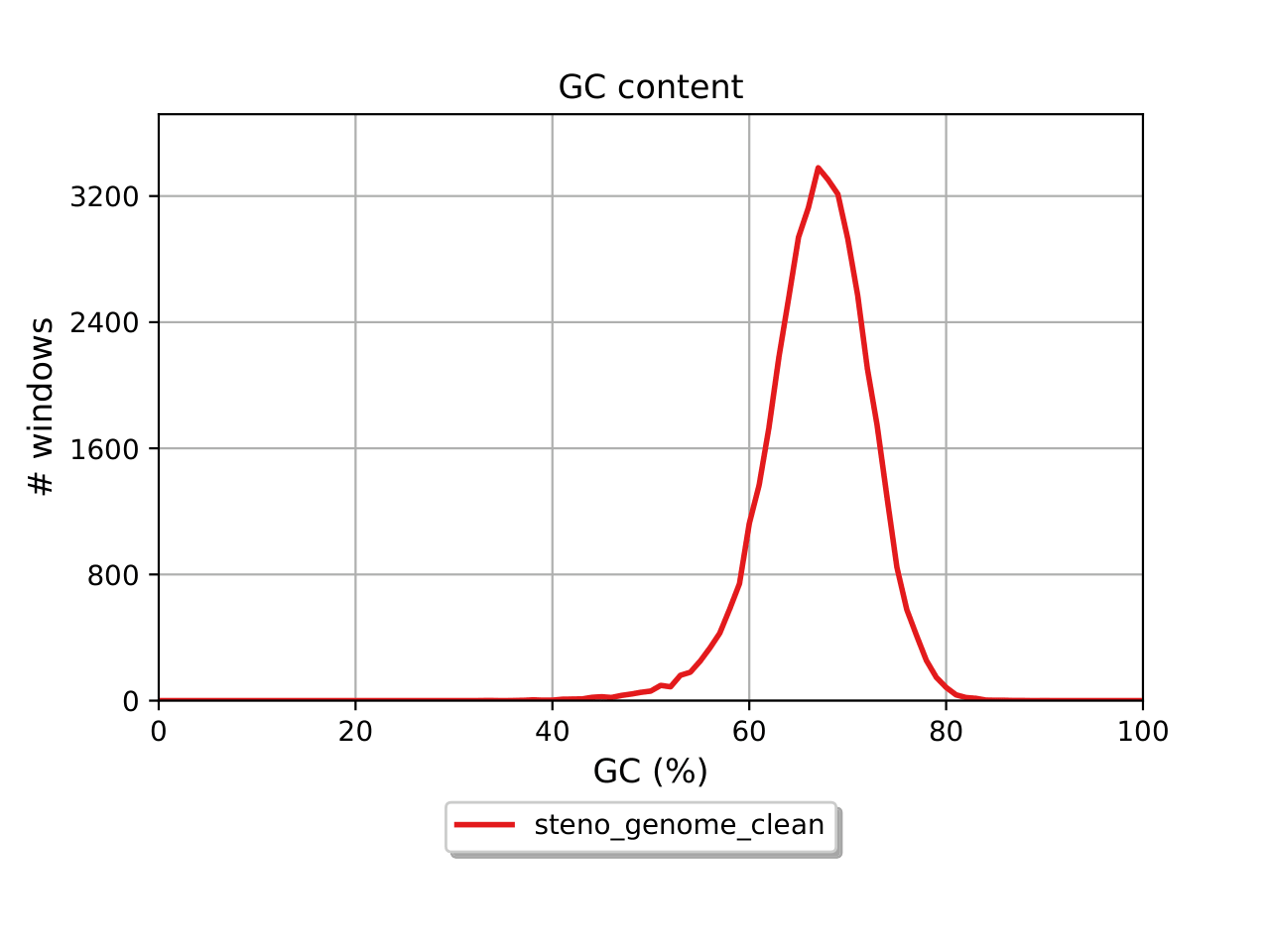


**Supplementary Fig. S3. Genome-wide GC content distribution for NG-SM01.** Smoothed frequency plot of GC% across sliding windows spanning the NG-SM01 genome, showing a unimodal distribution centered in the high-GC range, consistent with a uniform genomic base-composition background.


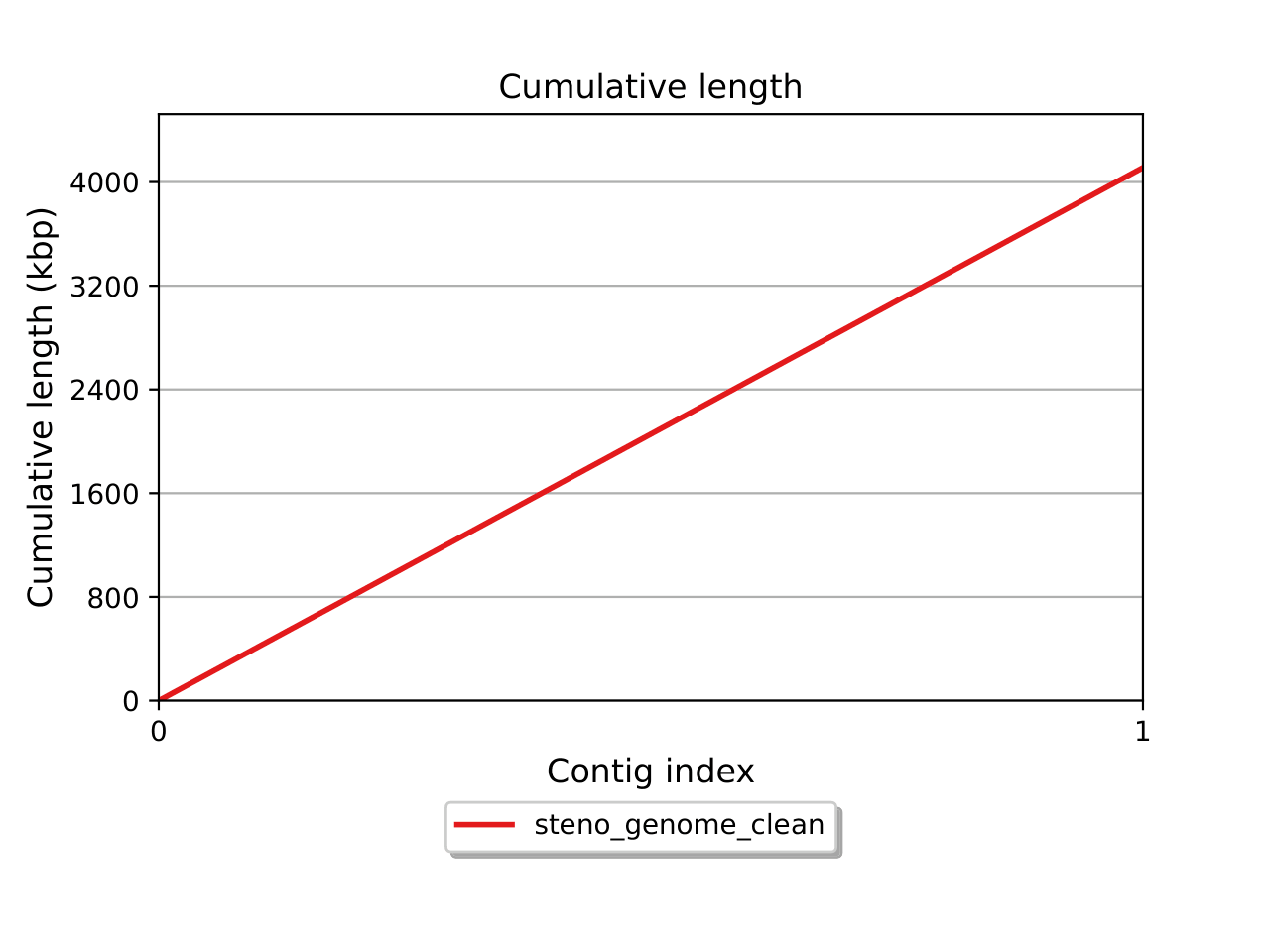


**Supplementary Fig. S4. Cumulative assembly length profile for NG-SM01.** Cumulative contig length (kbp) plotted against contig index after sorting by contig length. The linear rise to the full genome size with a single step is consistent with a single-contig assembly.

**
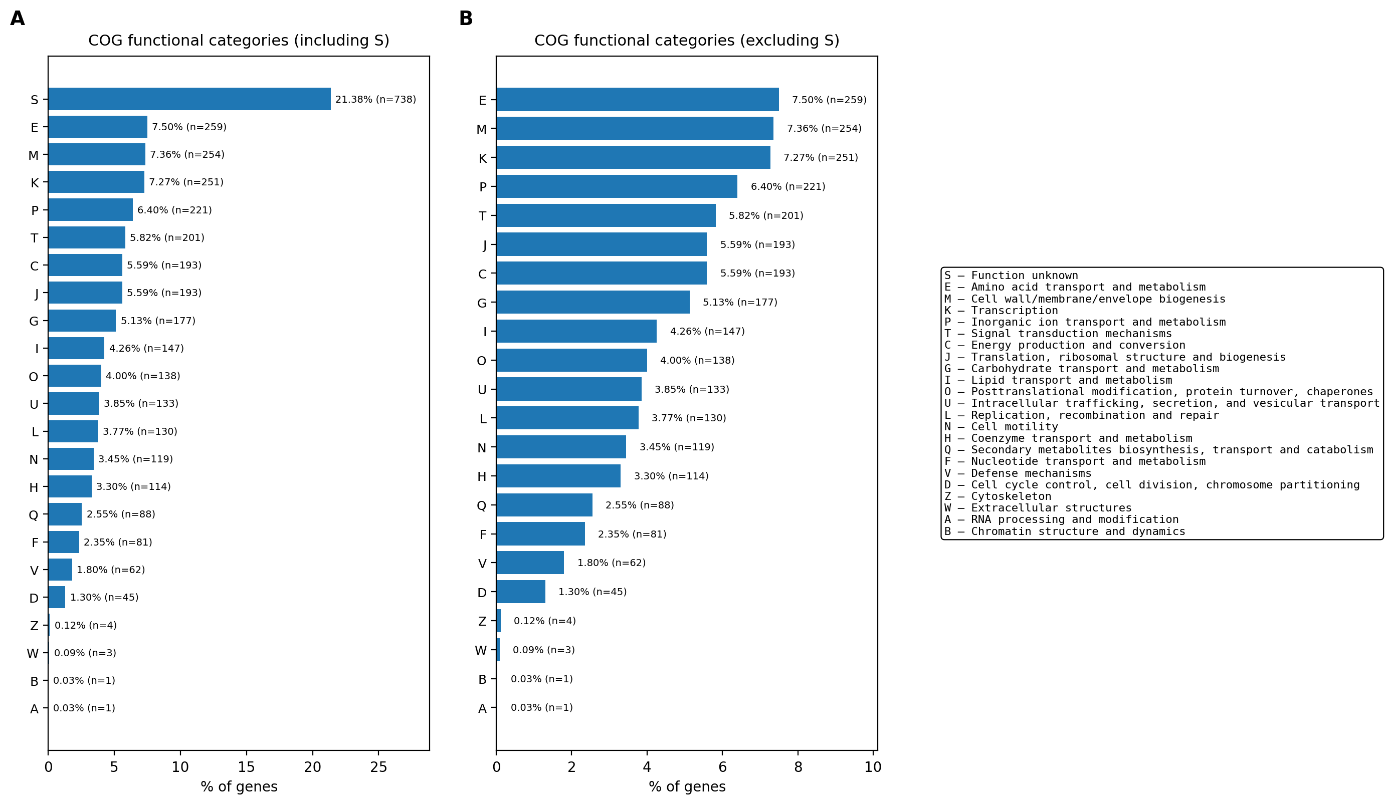
**

**Supplementary Fig. S5. COG functional category distribution in NG-SM01. (A)** Relative abundance of eggNOG-assigned COG categories, including category S (function unknown), shown as percentages with gene counts (n). Genes lacking any COG assignment (n = 228; 6.60%) were excluded. **(B)** The same distribution excluding category S to emphasize the relative contributions of annotated functional groups; bars indicate the percentage and gene count (n) for each COG category.


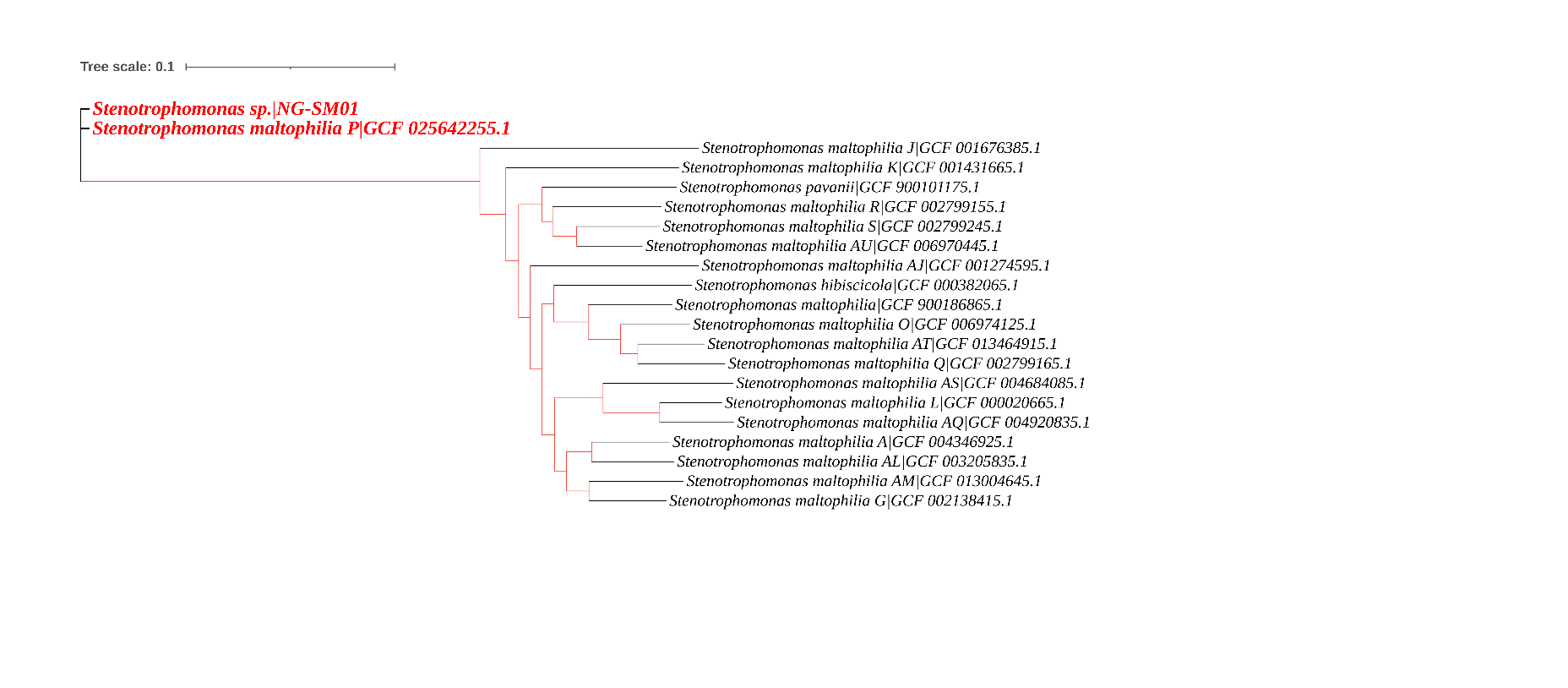


**Supplementary Fig. S6. Core-genome phylogeny excluding the NBRC 14161 type-strain anchor.** Maximum-likelihood tree constructed as in Fig. 3 but excluding the type strain. The placement of NG-SM01 adjacent to the same closest reference lineage is retained, supporting topology stability under alternative panel composition.


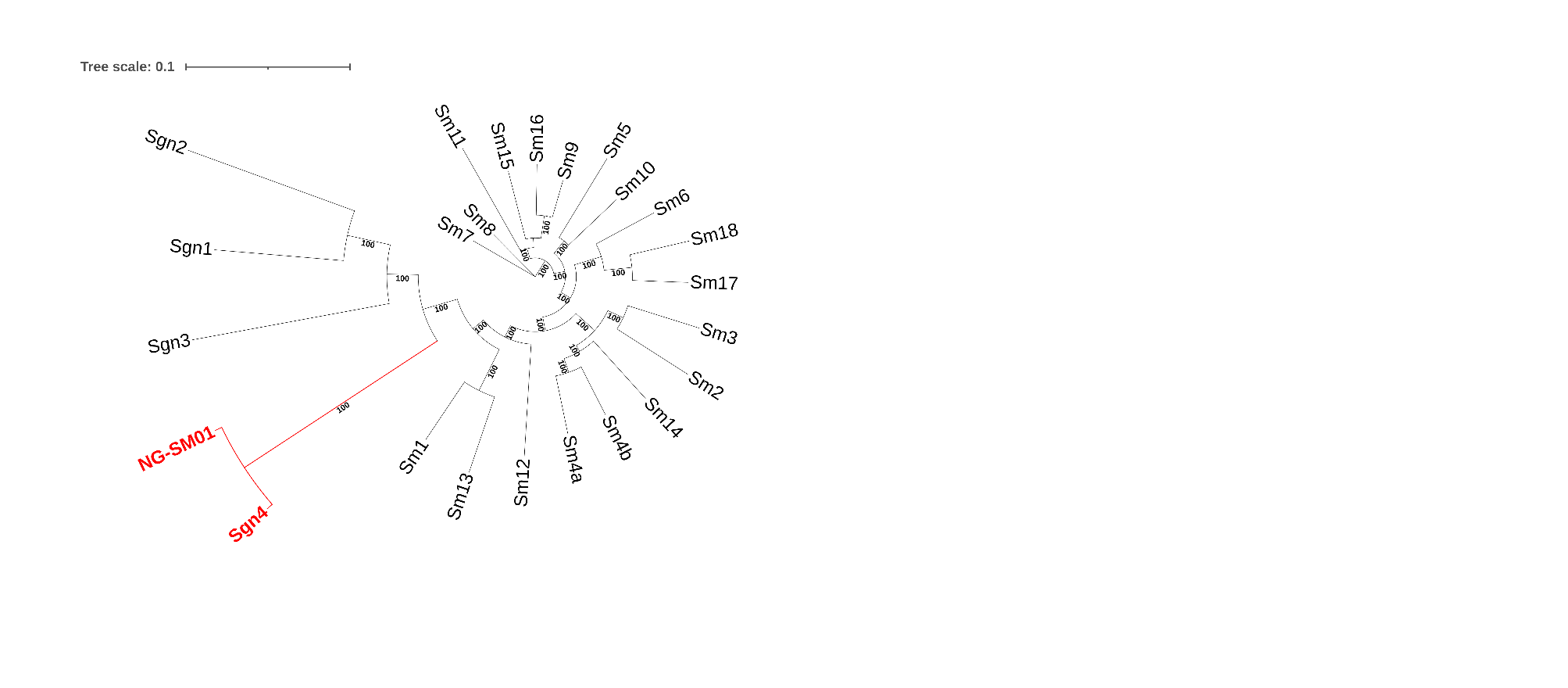


**Supplementary Fig. S7. Lineage-anchored phylogenomic placement of NG-SM01 within the *S. maltophilia* complex.** Maximum-likelihood tree inferred from the filtered Panaroo core-genome alignment using IQ-TREE2, with representative genomes spanning the 23 defined lineages (Sm1–Sm18; Sgn1–Sgn4, including Sm4a/Sm4b). Only NG-SM01 is shown as an isolate label; all other tips are labeled by lineage. Node labels indicate ultrafast bootstrap support. NG-SM01 clusters within the Sgn4 genomospecies and forms a maximally supported sister relationship with the Sgn4 anchor (UFBoot = 100). Scale bar indicates substitutions per site.


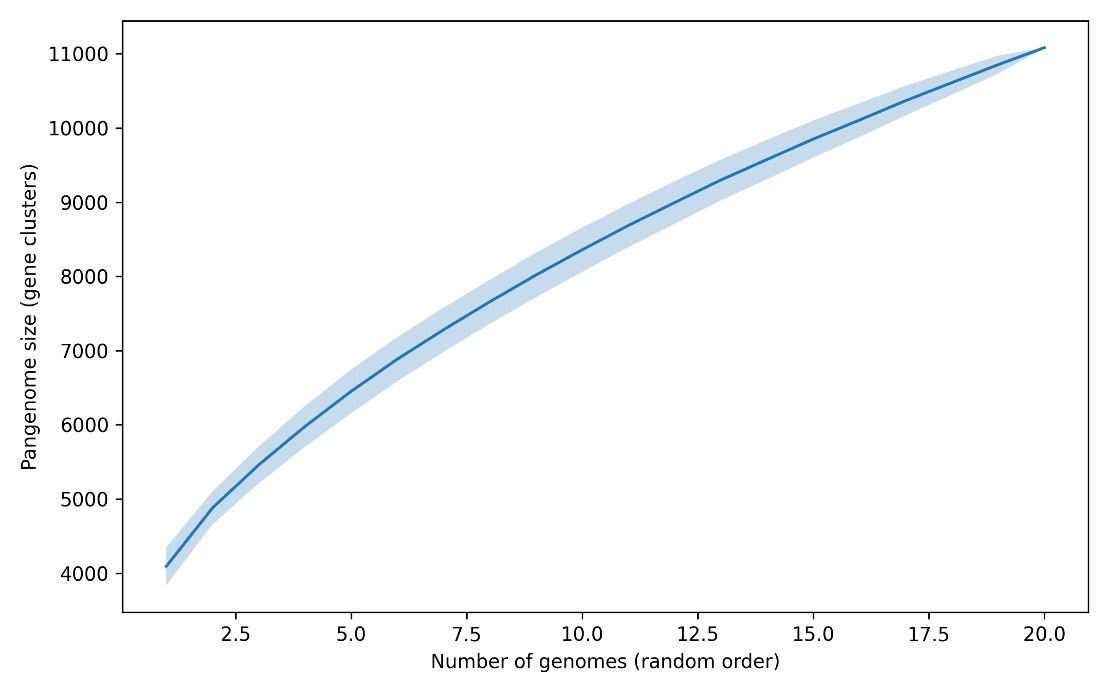


**Supplementary Fig. S8. Pangenome rarefaction curve across the NG-SM01–centered 20-genome panel.** Mean pangenome size (gene clusters) as a function of the number of genomes sampled, computed across 500 random genome-order permutations. Shaded band indicates dispersion across permutations.


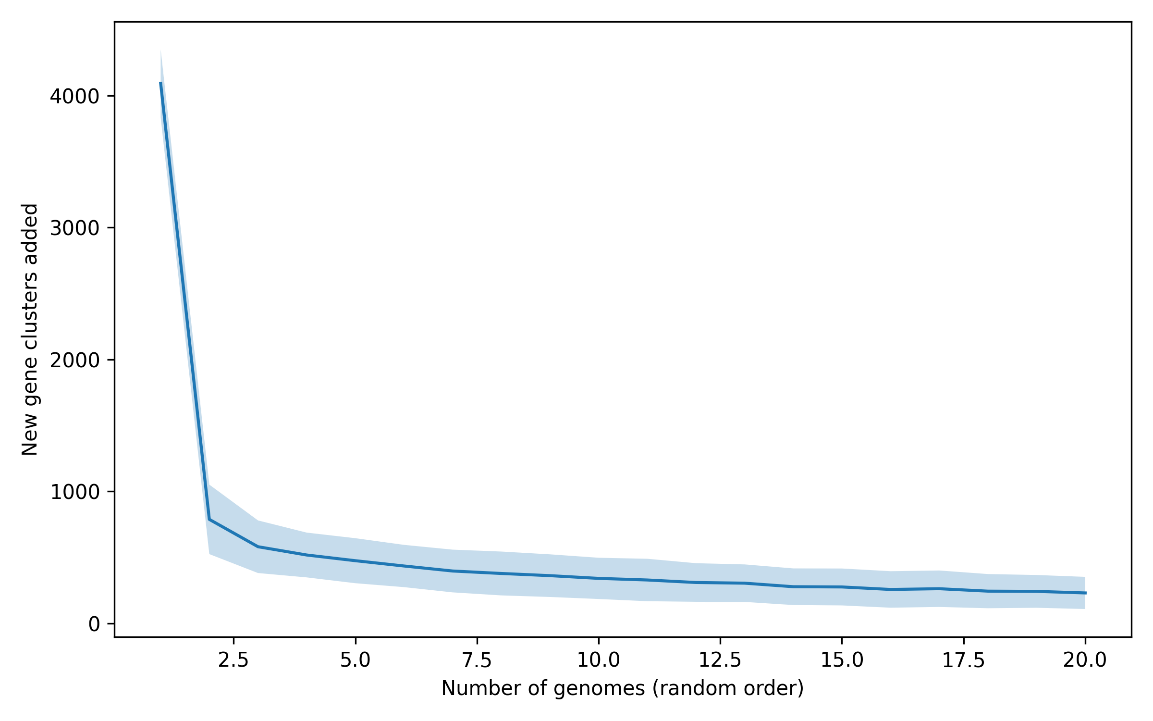


**Supplementary Fig. S9. Gene discovery curve across the NG-SM01–centered 20-genome panel.** Mean number of newly discovered gene clusters contributed by each additional genome, computed across 500 random genome-order permutations. Shaded band indicates dispersion across permutations.

**
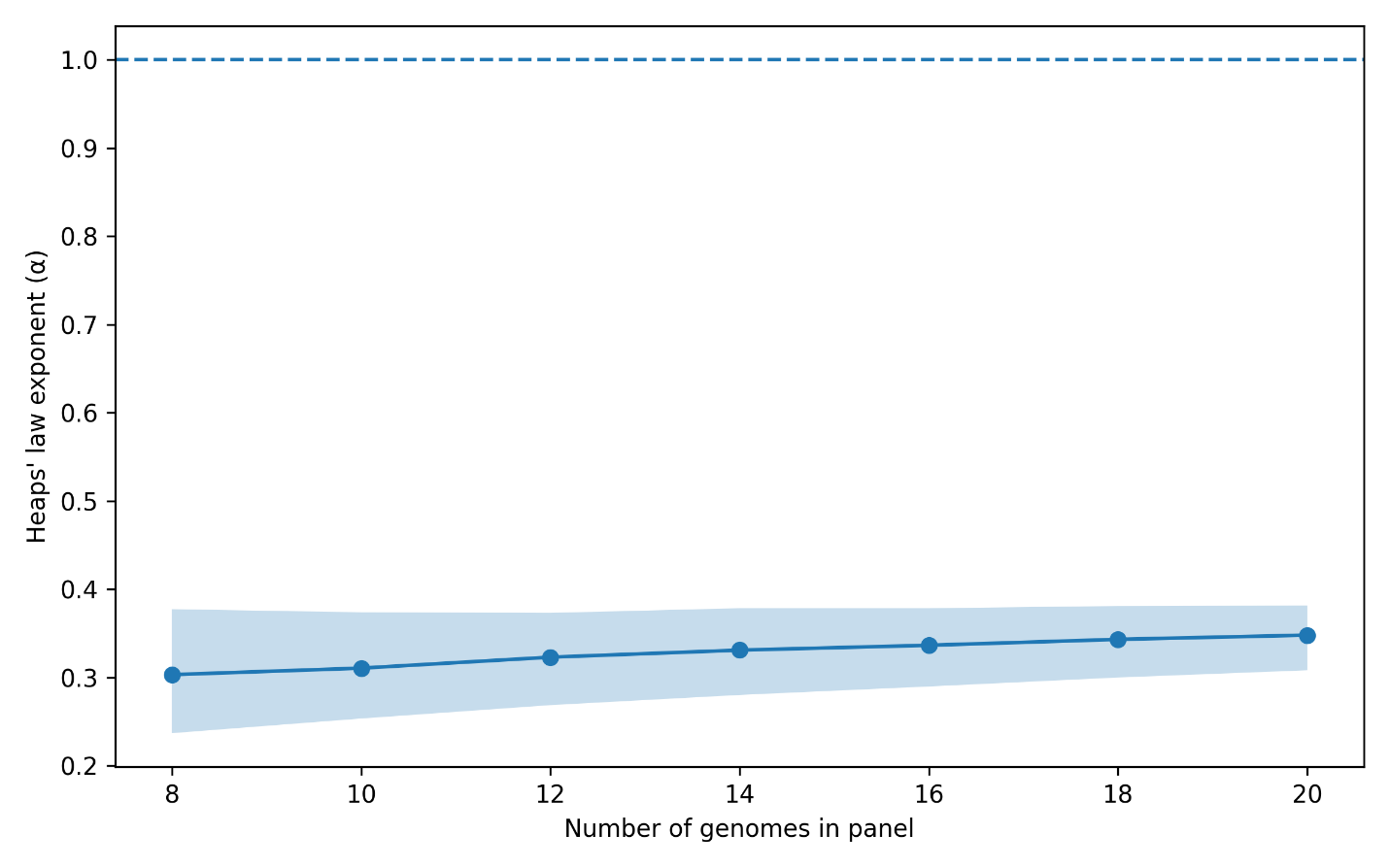
Supplementary Fig. S10. Heaps’ law exponent (α) across nested sampling depths supports an open pangenome.** Estimated Heaps exponent α for nested subsets of the panel (8–20 genomes) with uncertainty bands; the dashed line marks α = 1 (closure boundary). Values remain below 1 across sampling depths, consistent with an open pangenome.


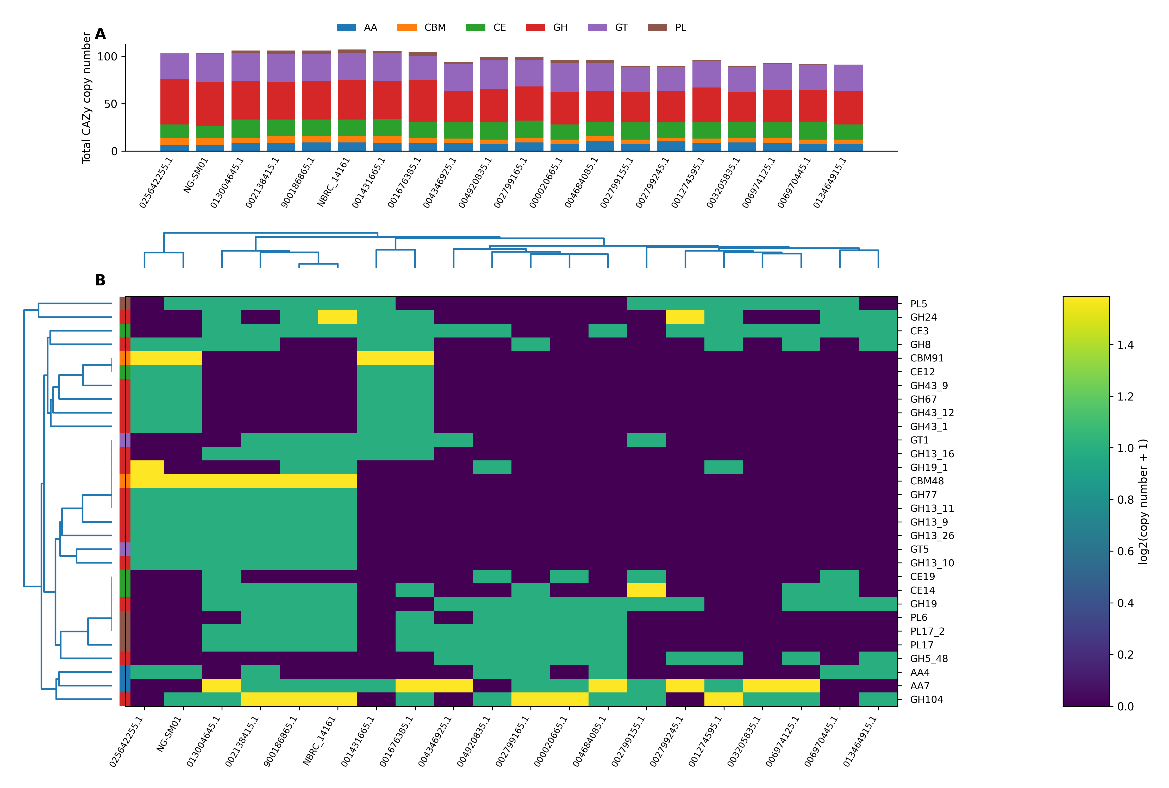


**Supplementary Fig. S11. CAZyme class composition and family-level variability across the NG-SM01–centered *S. maltophilia* complex panel (n = 20 genomes). (A)** Stacked bar plot showing per-genome CAZyme class copy numbers (GH, GT, CE, PL, AA, CBM); bar height represents total CAZyme burden and colored segments indicate class contributions. **(B)** Heatmap of the 30 most variable CAZyme families across genomes (ranked by variance of log2[copy number + 1]) with hierarchical clustering of genomes and families; color scale denotes log2(copy number + 1). Together, the panels show a conserved class-level backbone dominated by GH/GT with modest between-genome shifts, while most differentiation is concentrated in a small set of patchy, high-variance families (notably CBMs and PLs) that vary in presence and copy number across the panel.


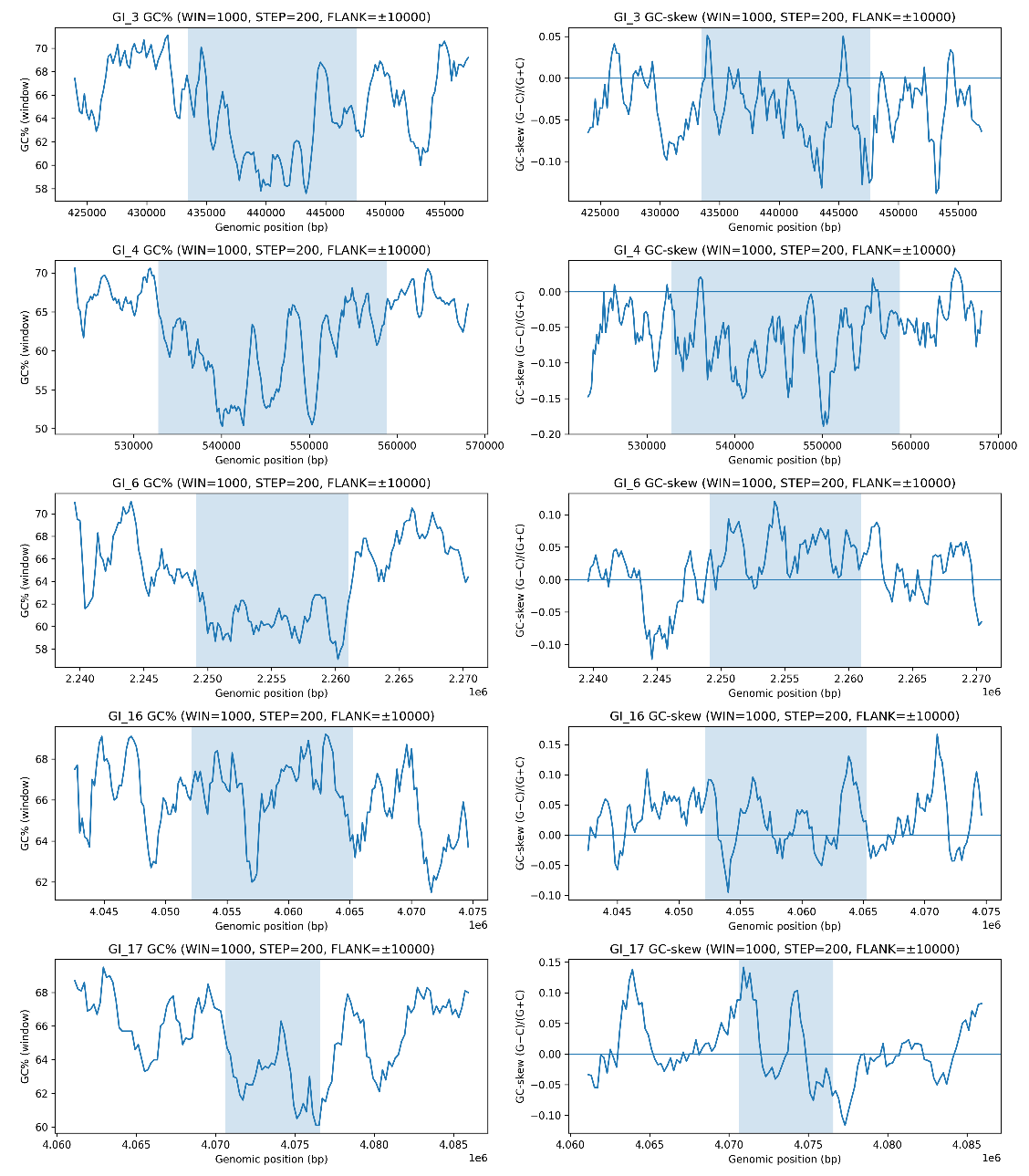


**Supplementary Fig. S12.** **Sliding-window GC% and GC-skew profiles across GI_3, GI_4, GI_6, GI_16, and GI_17 with ±10 kb flanks; shaded regions denote GI intervals.**


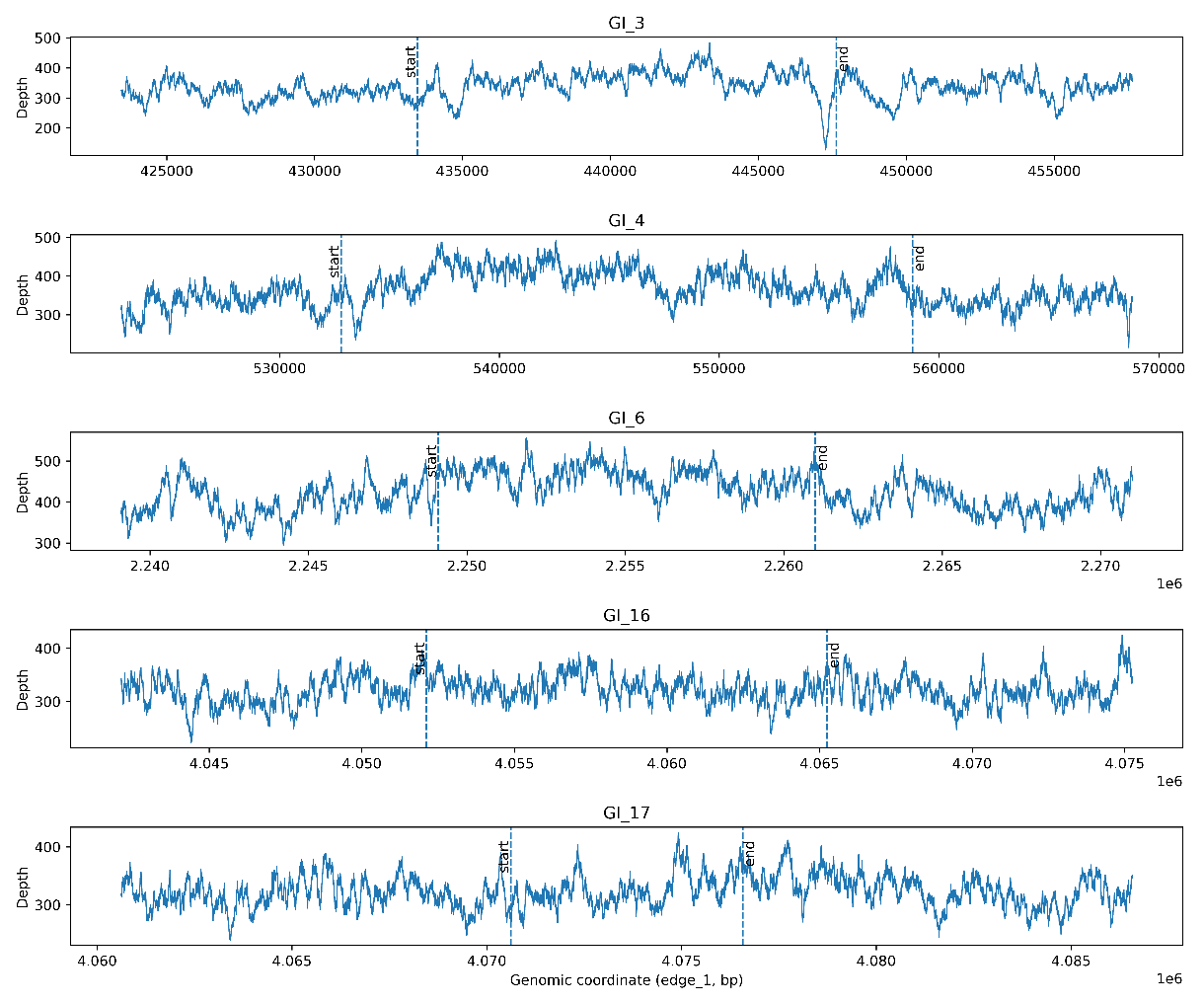


**Supplementary Fig. S13.** **Read-mapping depth traces across each prioritized GI (±10 kb context), marking GI start/end coordinates and supporting breakpoint integrity.**


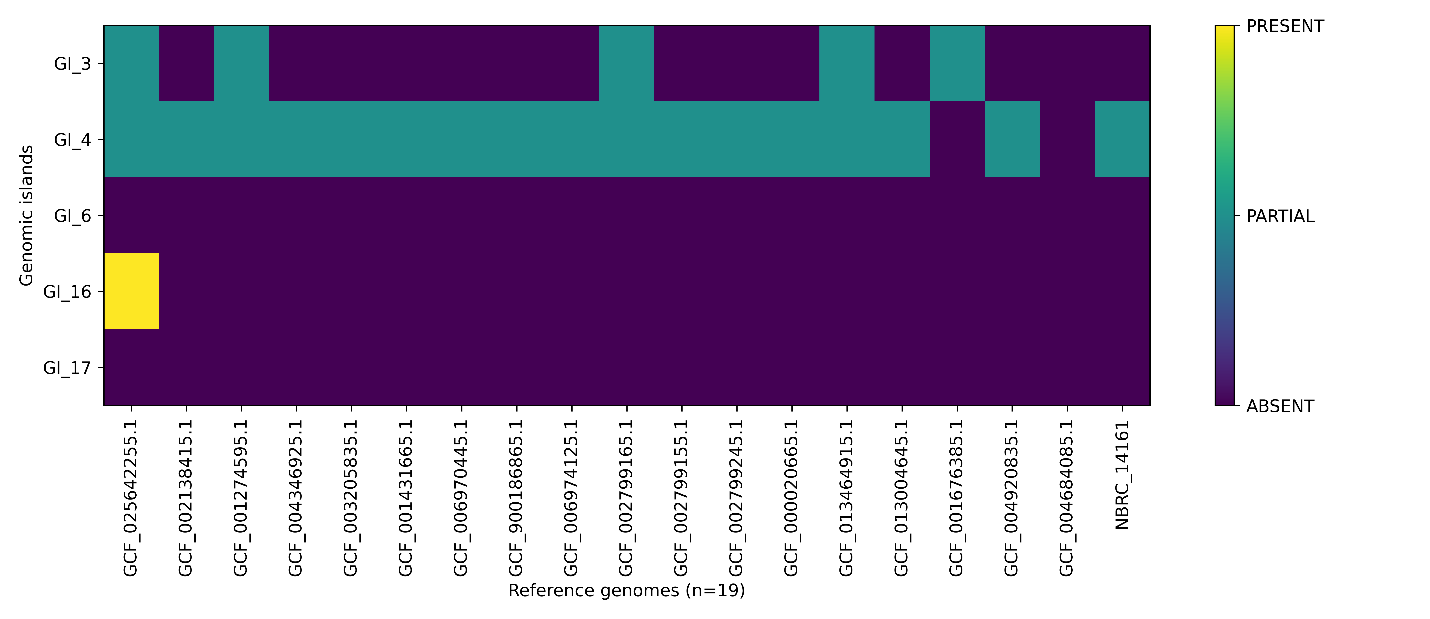


**Supplementary Fig. S14.** **Three-state (PRESENT/PARTIAL/ABSENT) heatmap showing prevalence of prioritized GIs across the 19-genome comparative panel.**


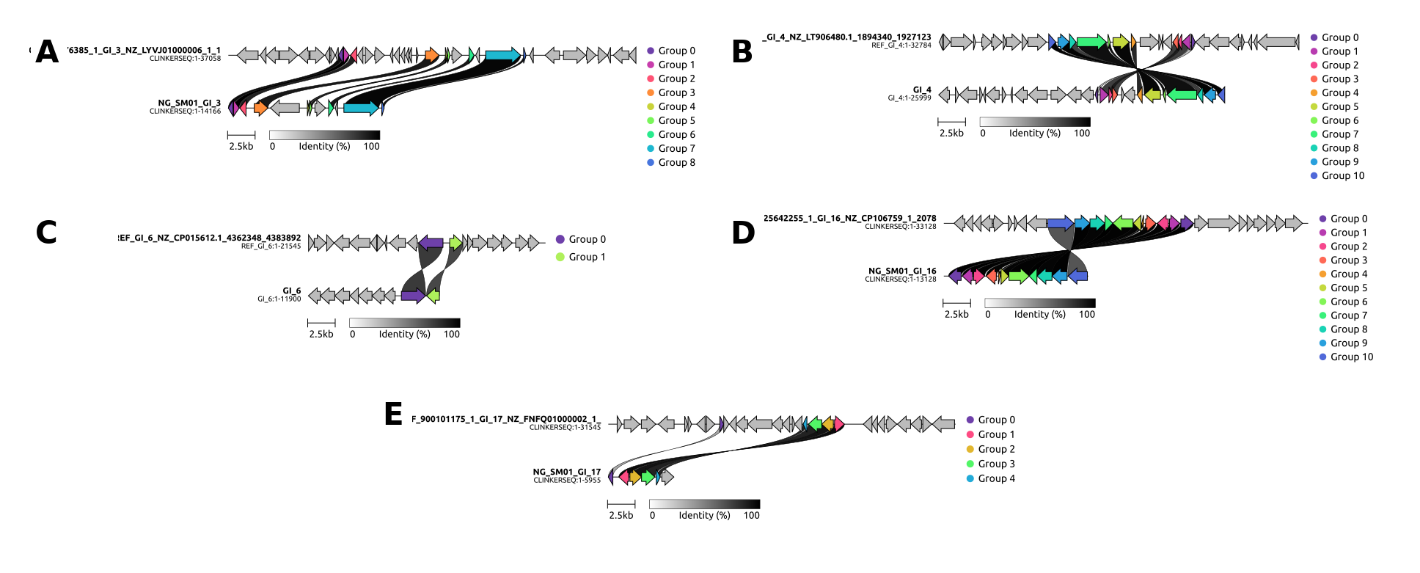
**Supplementary Fig. S15 (A–E). Clinker synteny comparisons of NG-SM01 priority islands against their best reference windows, illustrating locus-level gene order/orientation and the extent of shared gene blocks. (A)** GI_3 vs NZ_LYVJ01000006.1:177086–214143 (from GCF_001676385.1). **(B)** GI_4 vs NZ_LT906480.1:1894340–1927123 (from GCF_900186865.1). **(C)** GI_6 vs NZ_CP015612.1:4362348–4383892 (from GCF_002138415.1). **(D)** GI_16 vs NZ_CP106759.1:2078447–2111574 (from GCF_025642255.1). **(E)** GI_17 vs NZ_FNFQ01000002.1:82772–114316 (from GCF_900101175.1).


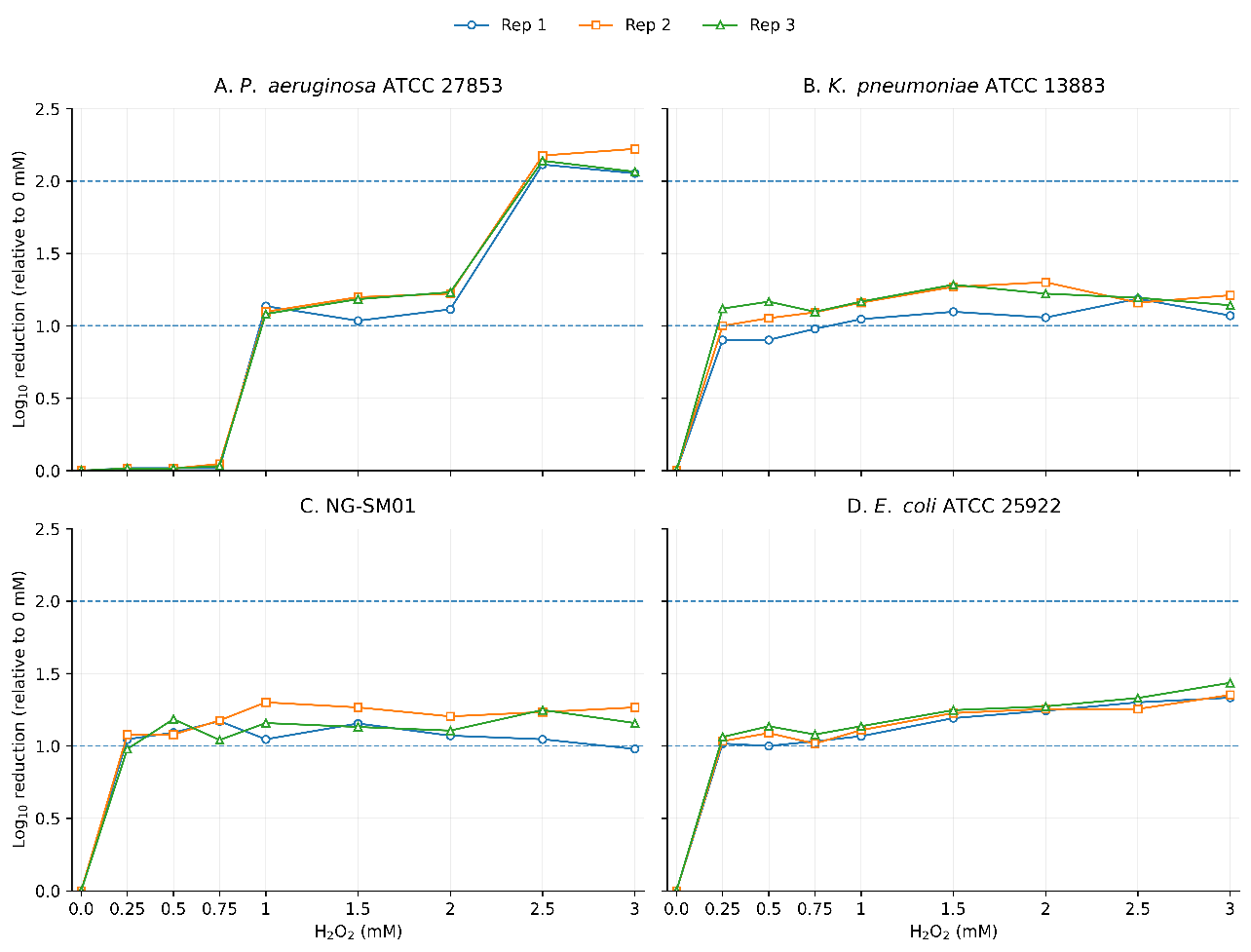


**Supplementary Fig. S16. Replicate-level H₂O₂ killing profiles.** Replicate-resolved log₁₀ reductions (relative to the matched 0 mM control) for each strain across 0–3.0 mM H₂O₂ after 5 min exposure (n = 3 biological replicates per strain).


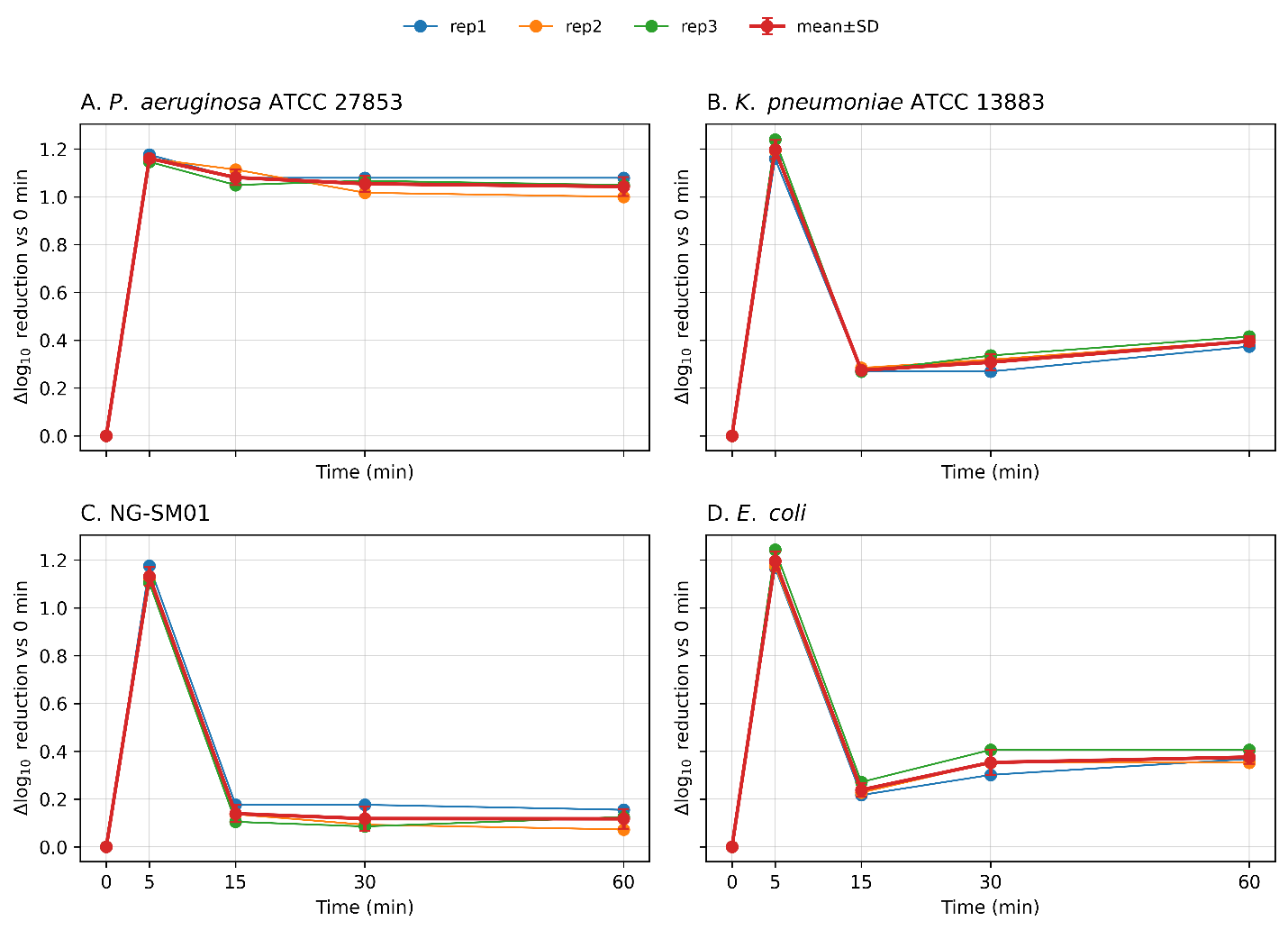
**Supplementary Fig. S17. Replicate-resolved time-kill trajectories in the H₂O₂ proxy assay.** Δlog₁₀ reductions vs 0 min are shown for each strain across 0–60 min using the same calculation as Fig. 12 (Δlog₁₀ = log₁₀[CFU/mL at 0 min; 0 H₂O₂] − log₁₀[CFU/mL at time *t*]). Panels show individual biological replicates (Rep1–Rep3) with mean ± SD overlay at each timepoint: **(A)** *P. aeruginosa* ATCC 27853, **(B)** *K. pneumoniae* ATCC 13883, **(C)** NG-SM01, and **(D)** *E. coli* ATCC 25922. Residual H₂O₂ was quenched with catalase at sampling prior to dilution/plating; n = 3 biological replicates per strain.
